# Plasma fatty acids and attention deficit hyperactivity disorder: a Mendelian randomization investigation

**DOI:** 10.3389/fpsyt.2024.1368942

**Published:** 2024-05-03

**Authors:** Kangning Zhou, Qiang Zhang, Zhenhua Yuan, Yurou Yan, Qian Zhao, Junhong Wang

**Affiliations:** ^1^ Graduate School, Beijing University of Chinese Medicine, Beijing, China; ^2^ Department of Pediatrics, Beijing Children’s Hospital, Capital Medical University, Beijing, China; ^3^ Department of Pediatrics, Dongzhimen Hospital, Beijing University of Chinese Medicine, Beijing, China

**Keywords:** fatty acids, n-3 PUFAs, ADHD, causality, Mendelian randomization

## Abstract

**Background:**

Attention deficit hyperactivity disorder (ADHD) is the most common neurodevelopmental disorder of childhood, and pathogenesis is not fully understood. Observational studies suggest an association between fatty acids abnormalities and ADHD, but there are contradictions and differences between these findings. To address this uncertainty, we employed a two-sample bidirectional Mendelian Randomization (MR) analysis to investigate the causal relationship between fatty acids and ADHD.

**Methods:**

We conducted a two-sample Mendelian Randomization (MR) study, selecting single nucleotide polymorphisms (SNPs) highly correlated with fatty acid levels from the CHARGE Consortium as our instruments. The outcome data were sourced from the Psychiatric Genomics Consortium (PGC) dataset on ADHD, comprising 225,534 individuals, with 162,384 cases and 65,693 controls. Inverse variance weighting, MR-Egger, and weighted median methods were employed to estimate the causal relationship between fatty acids and ADHD. Cochran’s Q-test was used to quantify heterogeneity of instrumental variables. Sensitivity analyses included MR-Egger intercept tests, leave-one-out analyses, and funnel plots.

**Results:**

The MR analysis revealed no significant associations between genetically predicted levels of various saturated, monounsaturated, and polyunsaturated fatty acids (including omega-3 and omega-6) and ADHD risk in the CHARGE and PGC cohorts. Notably, an initial association with Dihomo-gamma-linolenic acid (DGLA) (OR = 1.009, *p* = 0.032 by IVW) did not persist after correction for multiple testing (adjusted *p*-value = 0.286). Sensitivity analysis supported our findings, indicating robustness. Moreover, there was a lack of evidence supporting a causal link from ADHD to fatty acids.

**Conclusion:**

While our study on the basis of genetic data does not provide evidence to support the causal role of fatty acids in ADHD, it does not preclude their potential involvement in reducing the risk of ADHD. Further research is needed to explore this possibility.

## 1 Introduction

Attention deficit hyperactivity disorder (ADHD) is a life span disorder, and recognized as the most common neurodevelopmental disorder of childhood (1). Only a small proportion (15%) of individuals with ADHD achieve complete remission during their early adulthood (2, 3). It is marked by age-inappropriate levels of inattention, hyperactivity, and impulsivity, and it can lead to long term social, academic, and mental health issues (4, 5). According to a systematic review and meta-analysis study, the global incidence of ADHD is 7.6% in children aged 3 to 12 years and 5.6% in teenagers aged 12 to 18 (6). The fifth edition (DSM-5) of the Diagnostic and Statistical Manual of Mental Disorders released by the American Psychological Association provided a definition of ADHD as a consistent pattern of inattention and/or hyperactivity- impulsivity that hinders both development and functioning (7, 8). It is often comorbid with various psychological/mental disorders, such as oppositional defiant disorder (ODD), conduct disorder (CD), anxiety/depression disorder, learning disabilities (LD), and tic disorders (TD) among others (9–11).

Fatty acids are the major metabolic products of lipid metabolism (12). They are divided into three categories based on the number of carbon-carbon double bonds: saturated fatty acids, monounsaturated fatty acids (MUFAs), polyunsaturated fatty acids (PUFAs). Trans fatty acids (TFAs) are a general term of unsaturated fatty acids containing 1 or more trans nonconjugated double bond structure. There are two sources of TFAs: natural source and industrial source. Natural source means that trans fatty acids are present in meat or dairy products of ruminants, as some trans fatty acids are produced during the fermentation process (is a process of biological hydrogenation) in the rumen of ruminants. Industrial source means that trans fatty acids are present in margarine, cocoa butter substitutes, hydrogenated cream, and fried foods, as these trans fatty acids are produced through pathways such as partial hydrogenation of vegetable oils and high-temperature frying (13).

It is universally known that fatty acids composition and metabolism can be altered during diseases, leading to beneficial (14, 15) or adverse effects (16, 17). It has earlier been considered that altered fatty acids composition may be related to ADHD (18, 19). Observational studies found that PUFAs may be pertinent to the development of mental disorders, such as ADHD (20), autism spectrum disorder (ASD) (21), anxiety disorders (15) and Alzheimer’s disease (22). There is also research pointing a possible link between trans fatty acids and ADHD, where children with ADHD have higher levels of trans fatty acids than those without ADHD (23). Some meta-analyses indicate that children and adults with ADHD have elevated ratios of blood omega-6 to omega-3, indicating a disruption in fatty acid metabolism (14).However, the causal relationship between fatty acid abnormalities and the onset of ADHD remains unclear because of the common methodological problems in observation studies, such as residual confounding, reverse causality and misclassification (24, 25). For example, although there are a few observational studies suggesting the presence of fatty acid imbalances in children with ADHD (12, 19, 20, 23), yet conflicting observations regarding fatty acid imbalances in ADHD have been found in different studies. The contradictory result may be caused by residual confounding or reverse causality (26).

Mendelian randomization (MR) is a method that uses genetic variation as instrumental variables (IVs) to examine causal effects (24). Genetic variants are not easily influenced by confounding factors because it randomly assembled at the time of conception (25, 27), and the onset and progression of the disease do not alter the genetic variants. Therefore, MR minimizes biases from residual confounding and reverse causality in observational studies, thereby strengthening the causal inference of exposure-outcome associations (24, 28).

Several Mendelian randomization (MR) studies have investigated the complex relationships between fatty acids and other mental disorders. Some MR studies have revealed that long-chain omega-3 and omega-6 fatty acid levels are associated with a lower risk of schizophrenia (29, 30), while short-chain fatty acids are linked to an increased risk (30). One study has found genetically predicted increases in omega-3 levels were associated with a higher risk of epilepsy (31). Additionally, research on depression identified protective effects of adrenic acid and eicosapentaenoic acid (EPA), while oleic acid (OA) and alpha-linolenic acid (ALA) may be potential risk factors (32). Interestingly, a metabolome-wide MR study identifies dysregulated arachidonic acid synthesis as a potential causal risk factor for bipolar disorder (33). These findings underscore the multifaceted role of various fatty acids in mental disorder.

Given the complex associations and comorbidities among mental disorders (34), along with the shared common risk factors and genetic bases (35), further investigation into the association between fatty acids and ADHD is warranted. Understanding this relationship could provide valuable insights into the prevention and management of ADHD. MR can provide stronger evidence for the causal inference between fatty acids and ADHD. In this study, we applied a two-sample bidirectional MR design to further verify whether fatty acids abnormalities are associated with an increased risk of ADHD.

## 2 Materials and methods

### 2.1 Study design

**Figure 1:**
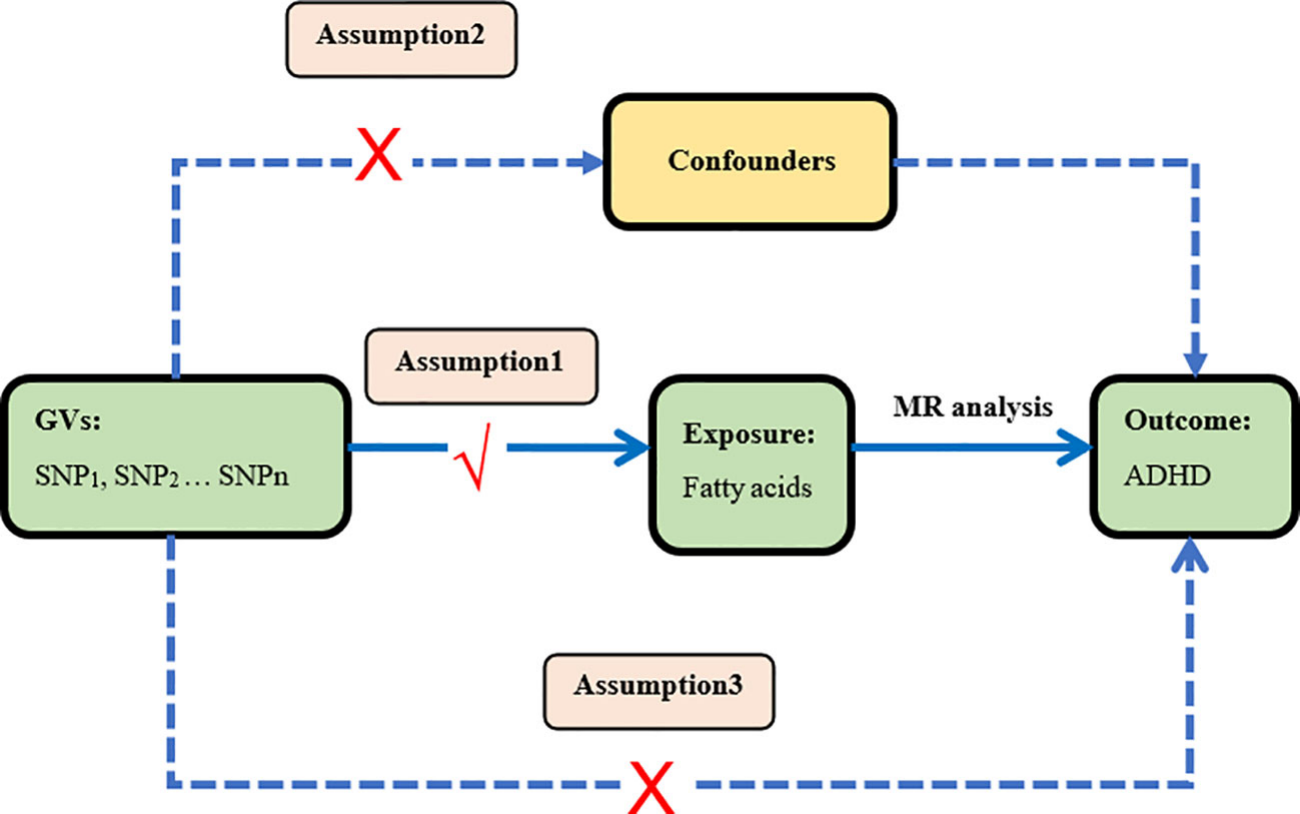
Study design This is the causal directed acyclic graph of MR design. The MR design assumptions are that the genetic variants are associated with fatty acids, but not with confounders, and the genetic variants are associated with the risk of ADHD only through fatty acids.

In this study, we performed a two-sample Mendelian randomization analyses using summary statistics from a genome-wide association study (GWAS) to investigate whether fatty acids would have a causal effect on ADHD. Genetic variants were used as instrument variables (IVs) to evaluate the causal effect of the exposure (fatty acids) on the outcome (ADHD). The validity of MR design hinges on three important assumptions that serve as the criteria for screening IVs (24, 28). Assumption 1: IVs are strongly associated with the exposure factors (fatty acids). Assumption 2: There is no correlation between IVs and any potential confounding factors. In short, IVs should be dependent of confounding factors. Assumption 3: IVs can affect outcomes only through exposure factors, not themselves or confounding factors. The study design of our experiment is shown in **Figure 1**.

### 2.2 Data source

#### 2.2.1 GWAS data of fatty acids (exposure)

We selected single nucleotide polymorphisms (SNPs) from fatty acid-related datasets to serve as IVs. The GWAS data on fatty acids were derived from three large-scale meta-analyses involving individuals of European ancestry, conducted by the Cohort for Heart and Aging Research in Genomic Epidemiology (CHARGE) consortium [n = 8916 individuals for saturated fatty acids (SFAs) or monounsaturated fatty acids (MUFAs), n = 8631 individuals for n-6 polyunsaturated fatty acids (PUFAs), and n=8866 individuals for n-3 PUFAs] (29–31). These data included two SFAs, palmitic acid (16:0) and stearic acid (18:0); two MUFAs, palmitoleic acid (16:1n7) and oleic acid (18:1n9) (36); four omega-3 PUFAs, alpha-linolenic acid (ALA) (18:3n3), eicosapentaenoic acid (EPA)(20:5n3), docosapentaenoic acid (DPA)(22:5n3), and docosahexaenoic acid (DHA)(22:6n3) (37); and three omega-6 PUFAs, adrenic acid (AdrA)(22:4n6), gamma-linolenic acid (GLA)(18:3n6), and dihomo-gamma-linolenic acid (DGLA)(20:3n6) (38).

#### 2.2.2 GWAS data of ADHD (outcome)

Data sources for attention-deficit/hyperactivity disorder (ADHD) were obtained from a genome-wide association study (GWAS) meta-analysis of 38,691 individuals with ADHD and 186,843 controls, published by the Psychiatric Genomics Consortium (PGC) (39). These data were combined from the extended Danish Integrative Psychiatric Research (iPSYCH) cohort (25,895 cases; 37,148 controls), the Icelandic deCODE cohort (8,281 cases; 137,993 controls) and 10 European cohorts aggregated by the PGC (4,515 cases; 11,702 controls). The iPSYCH cases were diagnosed with ADHD based on the ICD10 diagnosis codes (F90.0, F90.1, F90.8) and were identified in the Danish Psychiatric Central Research Register and the National Patient Register. The deCODE cases were clinically diagnosed with ADHD according to the ICD10 criteria (ICD10-F90, F90.1, F98.8) or were prescribed medication specific for/to ADHD symptoms. The iPSYCH and deCODE controls were individuals without ADHD. The PGC cases were derived from10 PGC cohorts with European ancestry as a part of a previous GWAS meta-analysis of ADHD. All participants who donated samples provide informed consent. The study identified 27 genome-wide significant loci. The data sources and sample information used in our study are detailed in **Table 1**.

**Table 1:** Information on genetic instruments and outcome source.

Category	Trait	Participant	Population	Consortium
Exposures	SFA	8,916	European	CHARGE
	MUFA	8,916	European	CHARGE
	Omega-3 PUFA	8,866	European	CHARGE
	Omega-6 PUFA	8,631	European	CHARGE
Outcome	ADHD	225,534 (38691 cases and 186,843 controls)	European	PGC

### 2.3 Selection of instrumental variables

**Figure 2:**
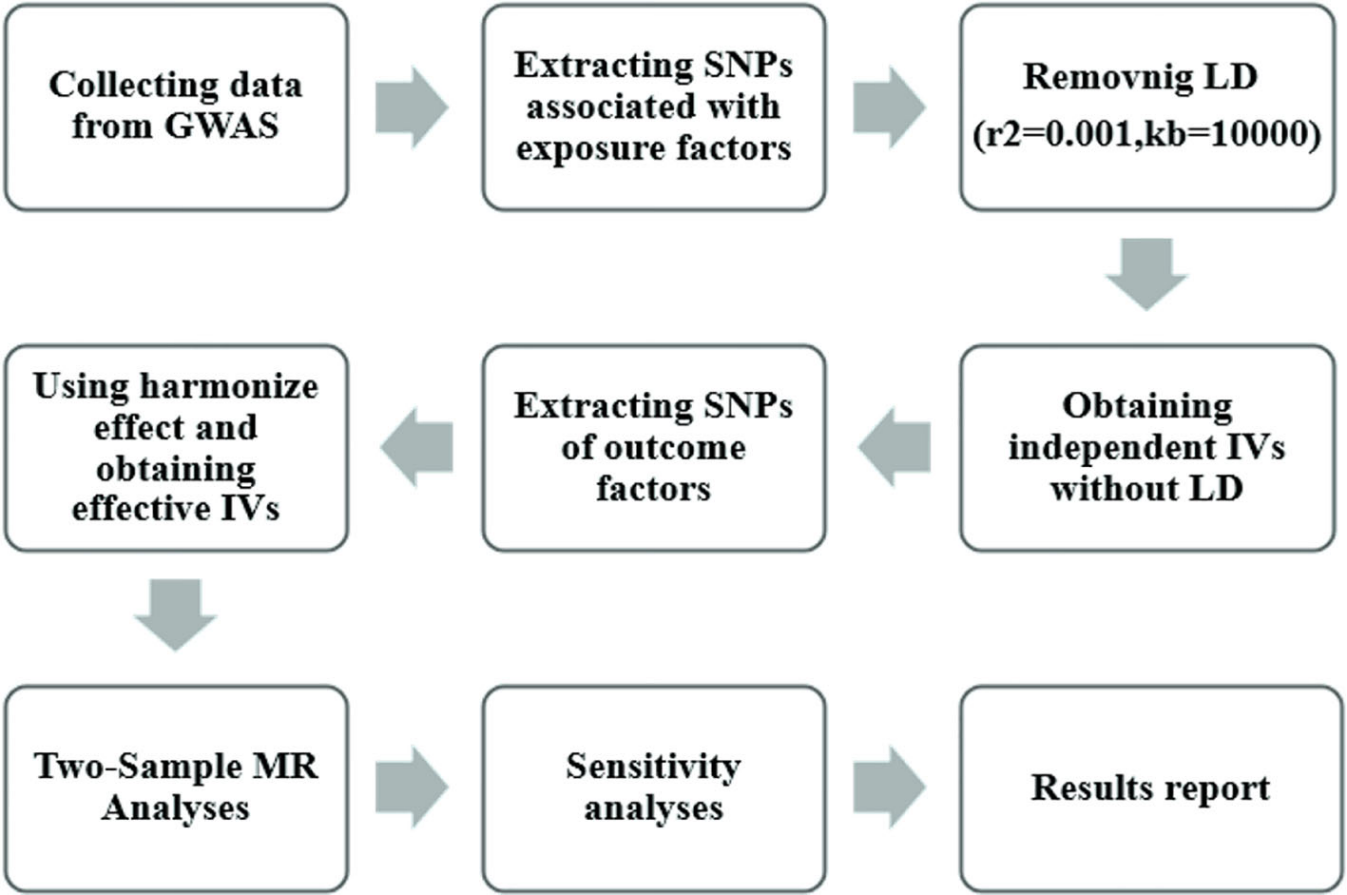
Mendelian randomization study flowchart. The gray boxes denote research steps, while the gray arrows signify the general direction. GWAS, genome-wide association study; IV, instrumental variable; LD, linkage disequilibria.

**Figure 2** illustrates the research workflow. We initially selected instrumental variables (IV) from the 11 exposures, requiring single-nucleotide polymorphisms (SNPs) with genome-wide significant associations with exposure *p* < 5×10^-8. However, for all 9 exposures except n-6 PUFA DGLA and n-6 PUFA GLA, only 1-4 SNPs meet this criterion. To ensure a sufficient number of IVs for sensitivity analyses and potentially identify more causal association, the threshold for these 9 exposures was loosened to a threshold of 5×10^-5.[18].

Secondly, we eliminated linkage disequilibrium among the screened SNPs by applying thresholds of r2 < 0.001 and kb > 10,000, resulting in independent IVs free from linkage disequilibrium. Subsequently, we utilized the online database PhenoScannerV2 (http://www.phenoscanner.medschl.cam.ac.uk/) to identify potentially related phenotypes. SNPs associated with ADHD outcomes and confounding factors were then filtered out using criteria of r2 ≥ 0.8, none proxies and p-value < 0.001. In the context of the relationship between fatty acids and ADHD, factors such as genetics, brain structure and function, premature birth and low birth weight, exposure to tobacco smoke and alcohol during pregnancy, and lead exposure are potential and significant confounding factors. All outlier and palindromic SNPs were removed.

Subsequently, we extracted the effect estimates of the selected instrumental variables (IVs) from the ‘‘ADHD outcome’ dataset and excluded SNPs with palindrome structures. To adhere to the Mendelian first hypothesis, we employed R2 as a genetic tool to elucidate the proportion of trait variance. The R2-value, representing the proportion of phenotypic variations explained by each SNP, was calculated using the formula (40, 41):


$$\;{\text{R}}^{2}=\sum^{\;}\left[2\times \left(1-\text{MAF}\right)\times \text{MAF}\times {\text{β}}_{2}÷\left(\text{S}{\text{E}}^{2}\times \text{N}\right)\right]$$

where SE and β represent the standard error and β coefficient for effect size, MAF is the minor allele frequency for each SNP, and N is the sample size. Next, we calculated an F-statistic to assess the overall strength of the selected SNPs in explaining phenotypic variations using the formula (42–44):


$$\text{F}=\left[\left(\text{N}-\text{K}-1\right)/\text{K}\right]\times \left[{\text{R}}^{2}/\left(1-{\text{R}}^{2}\right)\right]$$

where N is the sample size, k is the total number of SNPs selected for MR analysis, and R2 is the total proportion of phenotypic variations explained by all the SNPs. An F-statistic > 10 indicates that a SNP is a strong genetic instrument that can elucidate phenotypic variations and effectively reduce potential bias (42). Strong genetic instruments were chosen as the IVs of exposure phenotype for MR analysis. Additionally, we assessed the statistical power to estimate the genetically causal effects of fatty acids on ADHD risk using a web-based application, the mRnd power calculator (https://shiny.cnsgenomics.com/mRnd/) (45).

### 2.4 Statistical analysis

All our statistical analyses were conducted using the “Two Sample MR (version 0.5.8)” “data. Table (version 1.14.8)” and “MR-PRESSO (Mendelian Randomization Pleiotropy RESidual Sum and Outlier)” “LDlinkR” packages in R (2023 The R Foundation for Statistical Computing) (version 4.3.1). Reserved IVs were used to perform two-sample MR analyses.

Five MR methods, including inverse variance weighted (IVW), MR Egger, weighted median, simple mode, and weighted mode, were employed to analyze the causal influence of fatty acids on ADHD outcomes. Sensitivity analyses were conducted using established approaches such as leave-one-out analysis, PRESSO test, pleiotropy test, and heterogeneity test. The IVW method served as the primary analytical tool due to its demonstrated greater statistical power (46). This method assumes the validity and lack of horizontal pleiotropy for all instrumental variables, leading to more stable estimates. Consequently, IVW results were considered the main findings, while MR Egger, weighted median, simple mode, and weighted mode served as supplementary analyses.

Pleiotropy includes horizontal pleiotropy and vertical pleiotropy. Vertical pleiotropy implies that a genetic variant affects only a specific phenotype or feature without influencing others. On the other hand, horizontal pleiotropy suggests that a genetic variant affects multiple different phenotypes or features simultaneously. If horizontal pleiotropy exists, it implies that the genetic variant can influence other phenotypes besides the exposure, which are unrelated to the outcome. This would lead to a violation of the “no horizontal pleiotropy” assumption for the instrumental variable in MR analysis. This assumption essentially requires that the genetic variant only influences the outcome variable through its effect on the exposure. If a genetic variant with horizontal pleiotropy is used as an instrumental variable (IV), it can lead to biased estimates of the causal relationship between the exposure and outcome (47). Despite excluding known confounding SNPs, unknown confounding factors may still exist, leading to genetic polymorphism and biased effect size estimates. To satisfy the second and third hypotheses of MR, we employed MR-Egger for testing horizontal pleiotropy. The regression intercept reflects the magnitude of pleiotropy, with an intercept closer to 0 indicating a lower likelihood of pleiotropy. The P-value from the pleiotropy test signifies directional pleiotropy, and if *P* > 0.05, it indicates nonsignificant pleiotropy, suggesting that exposure is unlikely to affect the outcome through confounding factors or its own effects (48).

Heterogeneity indicates significant differences in the effects of different IVs on the outcome, affecting the stability of results. We utilized IVW and MR-Egger regression to test heterogeneity, evaluating it through the Cochran Q test’s Cochran Q value. *P* > 0.05 suggests the absence of heterogeneity (49, 50).

MR-PRESSO was employed to detect outliers and assess differences in estimated values before and after outlier removal, reducing the impact of outliers and enhancing study reliability. Additionally, it evaluates horizontal pleiotropy (*P* > 0.05 is considered indicative of no pleiotropy) (51).

Furthermore, leave-one-out test was applied for sensitivity analysis to demonstrate that the causal effect of fatty acids on ADHD outcomes is not influenced by individual SNP. Effect sizes in MR analysis were presented as odds ratios (OR) with 95% confidence intervals (CI).

## 3 Results

### 3.1 Causal effects of fatty acids on ADHD

After IVs selection, we conducted a two-sample Mendelian randomization study using the valid IVs and obtained results (Palmitic acid = 4, R2 = 0.650%, F = 14.421; Stearic acid = 4, R2 = 1.120%, F = 25.561; Palmitoleic acid = 5, R2 = 0.924%, F = 16.498; Oleic acid = 3, R2 = 0.975%, F = 29.145; Alpha-linolenic acid = 2, R2 = 1.745%, F = 78.540; Eicosapentaenoic acid = 1, R2 = 1.299%, F = 116.671; Docosapentaenoic acid = 5, R2 = 5.223%, F = 95.310; Adrenic acid = 5, R2 = 3.977%, F = 70.783; Dihomo-gamma-linolenic acid = 12, R2 = 8.277%, F = 60.866; Gamma-linolenic acid = 48, R2 = 16.831%, F = 30.435); (**Supplementary Tables 1–10**). We only obtained 1 usable IV for DHA after filtering on F-statistic. Therefore, we were unable to conduct MR analysis on n-3 DHA and ADHD.

**Figure 3:**
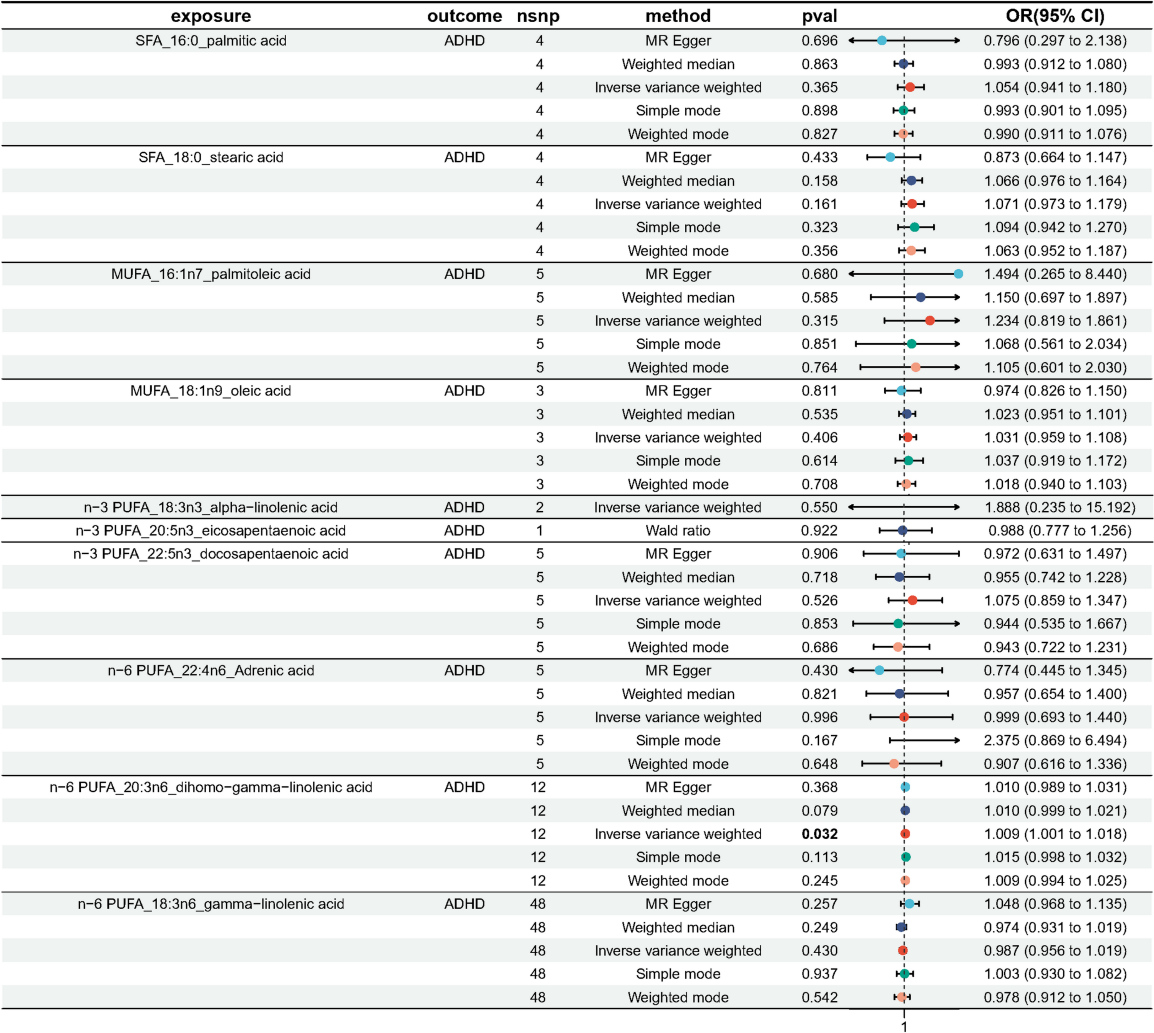
Odds ratio plot for genetic associations between 10 fatty acids and ADHD.

As plotted in **Figure 3**, the results of the IVW analysis revealed results for two types of SFA (16:0 (OR = 1.054, 95% CI 0.941 - 1.180, *p* = 0.365), 18:0 (OR = 1.071, 95% CI 0.973 - 1.180, *p* = 0.161)), and two types of MUFA (161:n7 (OR = 1.234, 95% CI 0.819 - 1.861, *p* = 0.315), 18:1n9 (OR = 1.031, 95% CI 0.959 - 1.108, *p* = 0.406)), two types of n-3 PUFA (ALA (OR =1.888, 95% CI 0.253 - 15.192, *p* =0.550), DPA (OR =1.075, 95% CI 0.859 - 1.347, *p* =0.526)) and two types of n-6 PUFA (AdrA(OR =0.999, 95% CI 0.693 - 1.440, *p* =0.996), GLA (OR =0.987, 95% CI 0.956 - 1.019, *p* = 0.430)), showing no causal relationship with ADHD. Genetically predicted EPA was also showed no causal association between EPA and ADHD (OR = 0.988, 95% CI 0.777 - 1.265, *p* = 0.922 by Wald ratio). MR analyses indicated a causal relationship between DGLA (OR =1.009, 95% CI 1.001 - 1.018, *p* = 0.032). Since we had multiple exposures, we performed FDR correction on this result to prevent the probability of false positives (using the Benjamini-Hochberg method). The adjusted P-value was 0.286, which not reached significance after adjustment. This suggests that DGLA is unlikely to be a risk factor for ADHD. Consistent conclusions were also provided by MR-Egger, MW, and four other methods **Figure 3**), indicating no association between genetically predicted fatty acid increase and increased risk of ADHD. MR-Egger intercept and MR-PRESSO did not reveal horizontal pleiotropy (P > 0.05) among all analyses, and no outliers were identified through MR-PRESSO. Except for palmitic acid (16:0) (*p* = 0.023), Cochran’s Q-test yielded P-values greater than 0.05 for the remaining fatty acids, suggesting no significant heterogeneity was observed. Despite the detection of heterogeneity in palmitic acid (16:0), utilizing the random-effects IVW method allowed for balancing the combined heterogeneity, making it acceptable (**Table 2**). Due to the limited number of available IVs for ALA and EPA, the tests for horizontal pleiotropy and heterogeneity could not be completed. To assess the robustness of our findings, we conducted a leave-one-out sensitivity analysis. In this analysis, we removed each SNP one at a time and re-estimated the causal effects. We observed no substantial changes in the overall effect estimates (**Supplementary Figures 1–3**). This suggests that our MR results are robust and reliable.

**Table 2:** Pleiotropy and heterogeneity test of fatty acids IVs in ADHD GWAS.

Fatty acids	nSNP	Heterogeneity test				Pleiotropy test		
		IVW		MR-Egger		MR-Egger intercept	*p*	MR-PRESSO Global test *p*
		Cochran's Q	*p*	Cochran's Q	*p*			
SFA 16:0	4	9.546	0.023	8.251	0.016	0.042	0.632	0.087
SFA 18:0	5	6.111	0.106	2.807	0.246	0.029	0.265	0.228
MUFA 16:1n7	5	1.826	0.768	1.777	0.620	-0.004	0.837	0.772
MUFA 18:1n9	3	0.786	0.672	0.242	0.623	0.009	0.593	–
n-3 ALA	2	3.318	0.069	–	–	–	–	–
n-3 EPA	1	–	–	–	–	–	–	–
n-3 DPA	5	4.444	0.349	4.024	0.259	0.004	0.615	0.458
n-6 AdrA	5	2.871	0.579	1.414	0.702	0.007	0.314	0.397
n-6 DGLA	12	15.40	0.165	15.397	0.118	-0.0005	0.961	0.173
n-6 GLA	48	50.784	0.327	48.137	0.386	-0.008	0.119	0.328

**Figure 4:**
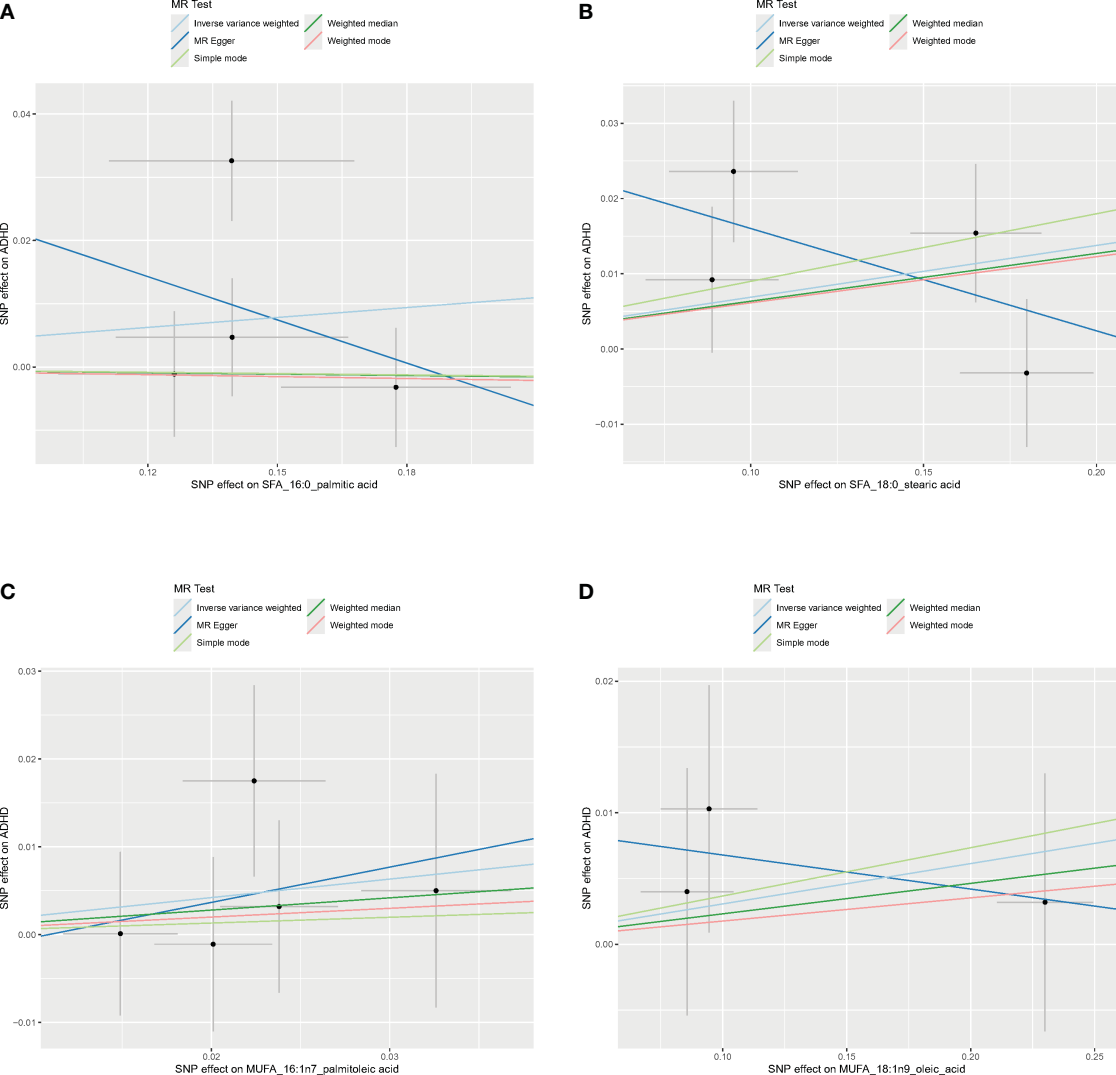
Scatter plots of SFA and MUFA. **(A)** Scatter plots of 16:0. **(B)** Scatter plots of 18:0. **(C)** Scatter plots of 16:1n7. **(D)** Scatter plots of 18:1n9. Scatter plots of the five MR results from the two SFAs and two MUFAs related to ADHD. Each point in the scatter plot represents an IV. The line on each point reflects the 95% CI, and the horizontal coordinate is the effect of SNPs on 16:0, 16:1n7, 18:0, 18:1n9. The vertical coordinate is the effect of SNPs on ADHD. SNP effects were plotted into lines for the inverse-variance weighted test (light blue line), MR-Egger regression (dark blue line), simple mode (light green line), weighted median (dark green line), and weighted mode (pink line).

**Figure 5:**
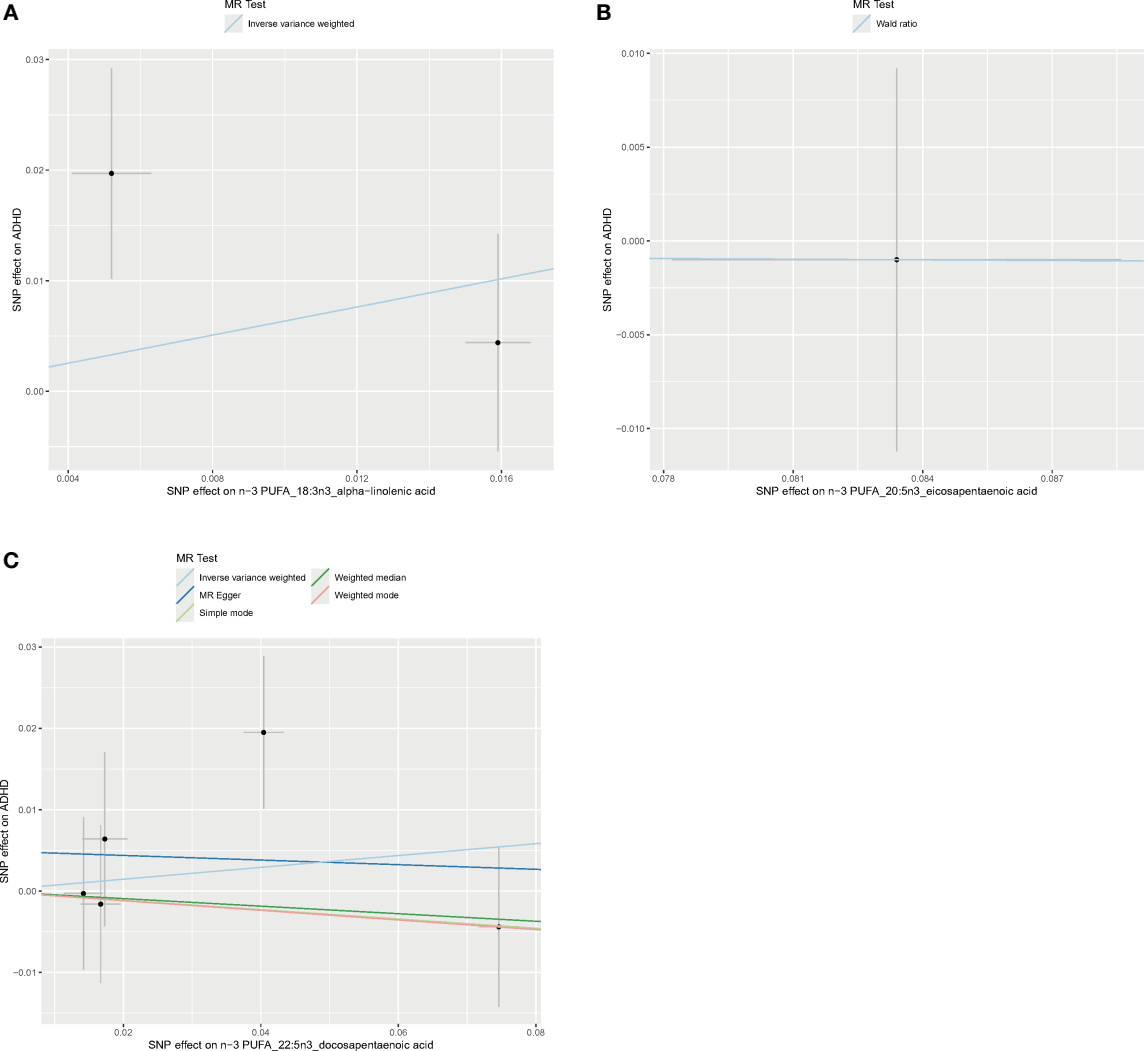
Scatter plots of n-3 PUFA. **(A)** Scatter plots of ALA. **(B)** Scatter plots of EPA. **(C)** Scatter plots of DPA. Each point in the scatter plot represents an IV. The line on each point reflects the 95% CI, and the horizontal coordinate is the effect of SNPs on ALA, DPA, EPA. The vertical coordinate is the effect of SNPs on ADHD. SNP effects were plotted into lines for the inverse-variance weighted test (light blue line), MR-Egger regression (dark blue line), simple mode (light green line), weighted median (dark green line), and weighted mode (pink line).

**Figure 6:**
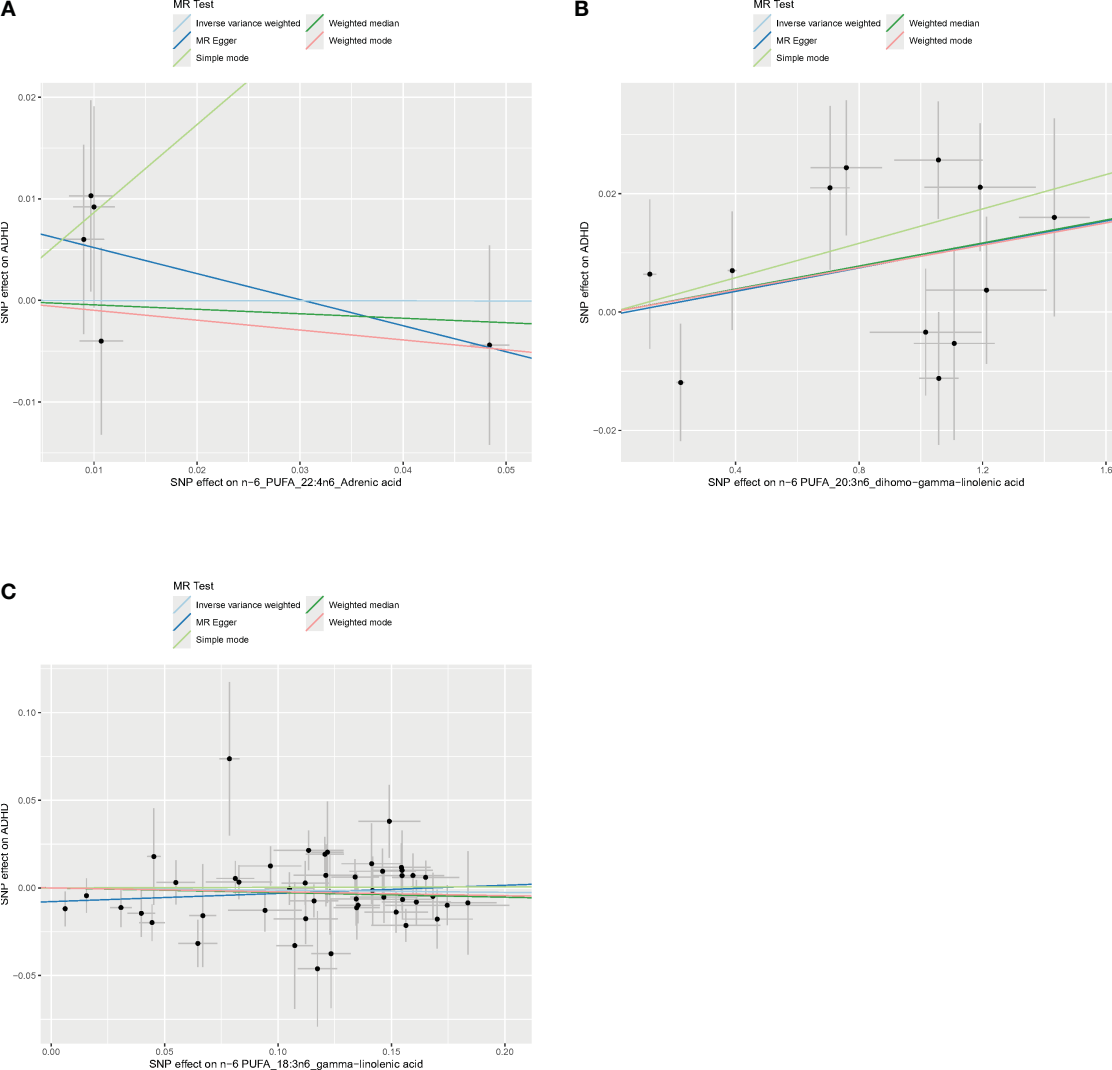
Scatter plots of n-6 PUFA. **(A)** Scatter plots of AdrA. **(B)** Scatter plots of DGLA. **(C)** Scatter plots of GLA. Scatter plots of the five MR results from the three n-6 PUFAs related to ADHD. Each point in the scatter plot represents an IV. The line on each point reflects the 95% CI, and the horizontal coordinate is the effect of SNPs on AdrA, DGLA, GLA. The vertical coordinate is the effect of SNPs on ADHD. SNP effects were plotted into lines for the inverse-variance weighted test (light blue line), MR-Egger regression (dark blue line), simple mode (light green line), weighted median (dark green line), and weighted mode (pink line).

**Figures 4**–**6** shows scatter plots of three types of n-3PUFAs, three types of n-6PUFAs, two types of MUFAs, and two types of SFAs with ADHD under different methods. Each point in the scatter plot represents an IV, and the line on each point represents a 95% confidence interval. The x-axis represents the SNP’s impact on the exposure factor (fatty acids), the y-axis represents the SNP’s impact on the outcome ADHD, and the colored lines indicate the MR fitting results. Forest plots and funnel plots of the individual SNP effects of fatty acids on ADHD are presented in **Supplementary Figures 4–9**.

### 3.2 Causal effects of ADHD on fatty acids

**Figure 7:**
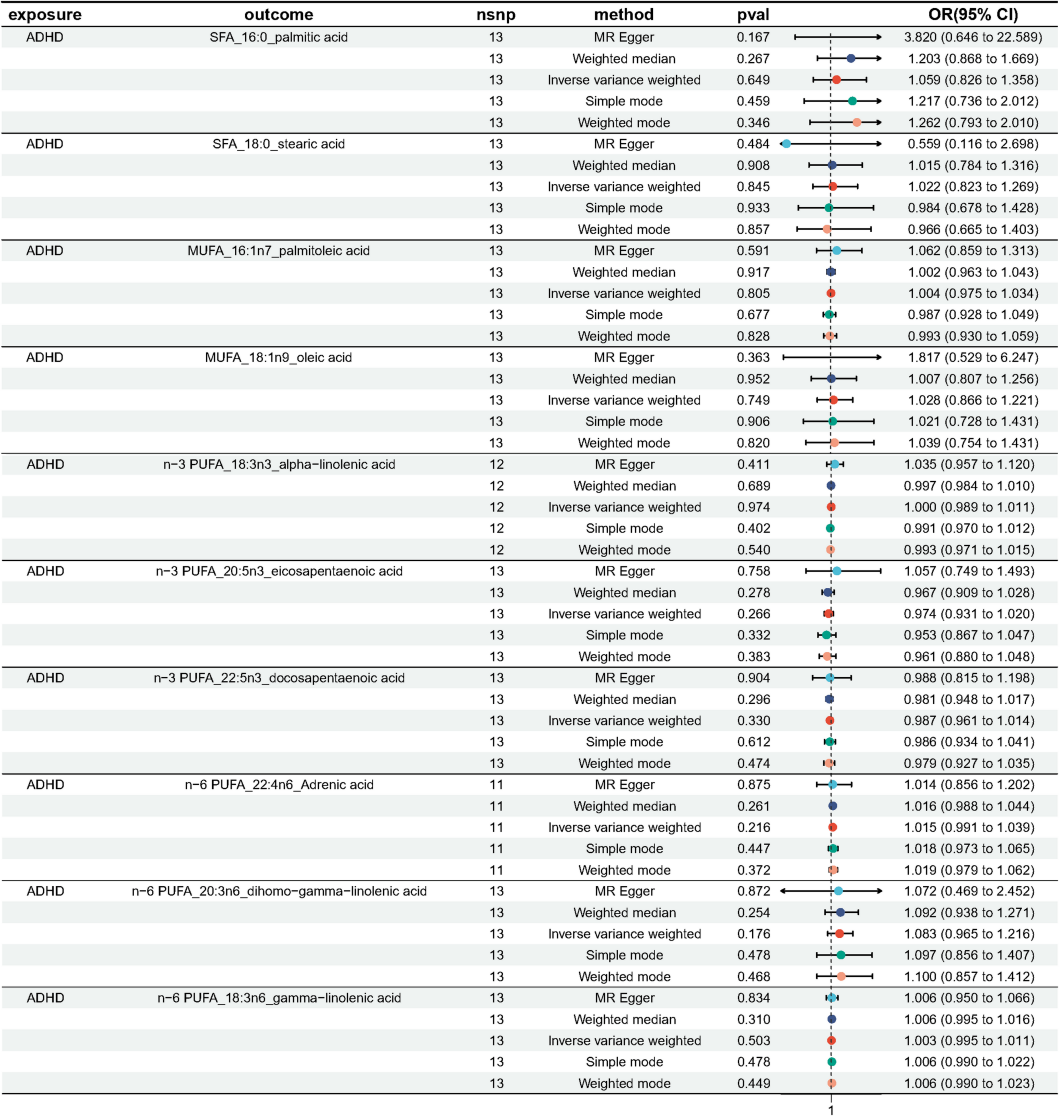
Odds ratio plot for genetic associations between ADHD and 10 fatty acids.

We performed MR analysis with ADHD as exposure to explore the possible reverse causality on fatty acids. As shown in **Figure 7**, genetically predicted ADHD was not associated with any fatty acid traits (Palmitic acid: OR = 1.059, 95% CI 0.826 - 1.358, *p* = 0.365; Stearic acid: OR = 1.022, 95%CI 0.823 - 1.269, *p* = 0.845; Palmitoleic acid: OR = 1.004, 95% CI 0.975 - 1.034, *p* = 0.805; Oleic acid: OR = 1.028, 95% CI 0.866 - 1.221, *p* = 0.749; ALA: OR = 1.000, 95% CI 0.989 - 1.011, *p* = 0.974; EPA: OR = 0.974, 95% CI 0.931 - 1.020, *p* = 0.266; DPA: OR = 0.987, 95% CI 0.961 - 1.014, *p* = 0.330; DHA: OR = 0.916, 95% CI 0.781 - 1.073, *p* = 0.275; AdrA: OR = 1.015, 95% CI 0.991 - 1.039, *p* = 0.216; DGLA: OR = 1.003, 95% CI 0.995 - 1.011, *p* = 0.503; GLA: OR = 1.054, 95% CI 0.941 - 1.180, *p* = 0.365). Neither heterogeneity nor pleiotropy was detected in the reverse directional MR analysis (**Table 3**). The scatter plots, forest plots, funnel plots and leave-one-out of the genetic variance are presented in **Supplementary Figures 10–21**.

**Table 3:** Pleiotropy and heterogeneity test of ADHD IVs in fatty acids GWAS.

Fatty acids	nSNP	Heterogeneity test				Pleiotropy test		
		IVW		MR-Egger		MR-Egger intercept	*p*	MR-PRESSO Global test *p*
		Cochran's Q	*p*	Cochran's Q	*p*			
SFA 16:0	13	5.932	0.919	3.891	0.973	-0.082	0.181	0.917
SFA 18:0	13	18.916	0.091	17.977	0.821	0.039	0.465	0.113
MUFA 16:1n7	13	8.862	0.715	8.588	0.660	-0.004	0.611	0.719
MUFA 18:1n9	13	8.353	0.757	7.520	0.756	-0.037	0.381	0.758
n-3 ALA	12	17.886	0.084	16.625	0.083	-0.002	0.404	0.082
n-3 DHA	13	18.367	0.105	18.135	0.078	0.014	0.715	0.119
n-3 EPA	13	12.014	0.445	11.779	0.380	-0.005	0.649	0.496
n-3 DPA	13	6.941	0.861	6.941	0.804	-7.688e-05	0.990	0.880
n-6 AdrA	11	14.261	0.161	14.260	0.113	4.00099e-05	0.994	0.178
n-6 DGLA	13	5.551	0.937	5.550	0.902	0.0007	0.981	0.934
n-6 GLA	13	7.245	0.841	7.230	0.780	-0.0002	0.119	0.907

## 4 Discussion

ADHD, one of the most prevalent neurodevelopmental disorders in children and adolescents, is typically diagnosed during childhood and persists into adulthood. The symptoms of ADHD can disrupt individuals’ learning, daily life, family, and employment, placing a significant burden on families. The causes of ADHD are multifaceted, involving factors such as genetics, environment, preterm birth, preeclampsia, hypoxia events, and maternal prenatal smoking exposure (52, 53). Extensive evidence from numerous cohort studies and meta-analyses published in recent decades supports the evaluation of pharmacological, non-pharmacological, and combined treatment options for managing ADHD (54–56). Multiple studies have shown that supplementing PUFA, especially n-3 PUFA, has a positive impact on improving ADHD symptoms and cognitive function (57–59). Due to the side effects of commonly used drugs for treating ADHD, many families are seeking alternative therapies for ADHD, such as supplementing with fatty acids.

We utilized MR to strengthen the inferences that can be drawn about the effect of SFA, MUFA, n-3 PUFA and n-6 PUFA on ADHD risk. Our study did not reveal a significant association between ADHD risk and levels of SFA (16:0 PA and 18:0 SA), MUFA (161: n7 PA and 181: n9 OA), n-3 PUFA (ALA, DPA, EPA) and n-6 PUFA (AdrA, DGLA, GLA). Specifically, we did not find any evidence that the 10 fatty acids examined in our study were associated with a reduced risk of ADHD. This finding is inconsistent with some previous observational studies, which reported protective effects of certain fatty acids against ADHD. However, other studies have also found limited efficacy PUFA in the treatment of ADHD, aligning with our results (60–64). A recent meta-analysis further supports this notion, indicating no improvement in core ADHD symptoms with n-3 PUFA supplementation (65). Nevertheless, it is undeniable that some studies have shown potential benefits of fatty acid supplementation, such as improved sleep (66). Additionally, MR studies have suggested protective effects of certain fatty acids against diseases such as schizophrenia and depression (29, 30, 32). Intriguingly, two recent MR studies examining the relationship between LA, DHA, and ADHD reached opposing conclusions (67, 68), highlighting the need for further research in this area.

One study has indicated a positive correlation between essential fatty acid deficiency and ADHD symptoms (69). Children with ADHD show more severe essential fatty acid deficiency, and the n-3 PUFA levels in ADHD patients are significantly lower compared to those in healthy control children (70). Our reverse Mendelian randomization study, designed to explore the potential impact of ADHD on fatty acids, found no genetic indication that ADHD leads to abnormal fatty acid levels.

The mechanisms underlying the therapeutic effects of fatty acids on ADHD remain unclear, although several potential pathways have been explored. Studies suggest that imbalances in omega-3 and omega-6 fatty acid levels in the blood of ADHD patients might contribute to the disorder, possibly due to disrupted fatty acid metabolism or increased inflammation (71, 72). Fatty acids also play a critical role in early brain development, influencing neuronal growth, communication between brain cells (synaptic function), and neurotransmitter signaling. Disruptions in fatty acid metabolism may hinder proper brain development and lead to some ADHD symptoms (73). Some studies propose that essential fatty acids can regulate brain cell signaling through monoamine modulation, signal transduction activation, and modulation of lipid rafts on cell membranes (74). Additionally, DPA and EPA have been shown to enhance anti-inflammatory effects by inhibiting free radical production and oxidative stress (75). Animal experiments suggest that EPA and DHA can restore a normal Firmicutes/Bacteroidetes ratio and improve stress-related inflammation by increasing the abundance of bacteria producing butyrate salts and reducing the levels of pro-inflammatory bacterial genera (76, 77). Moreover, DHA deficiency is associated with disturbances in the transmission of serotonin (5-hydroxytryptamine, 5-HT), norepinephrine, and dopamine, which may be related to cognitive impairments in ADHD (78). These findings, although suggestive of potential mechanisms, do not align with our study’s conclusion that there is no causal relationship between fatty acids and ADHD. However, this does not negate the possibility that other PUFA subtypes or a broader assessment of fatty acid metabolism may be relevant to ADHD. These findings highlight the need for further research to comprehensively understand the potential role of PUFA subtypes and broader fatty acid metabolism in ADHD risk.

In the present research, we employed an MR design to minimize residual confounding and reverse causation, improving causal inference regarding the correlation between fatty acids and ADHD. Utilizing ADHD data from the newly released PGC consortium, providing a large sample size for more robust evidence than observational studies. All analyses were confined within populations of European ancestry and genome-association tests adjusted for population stratification bias. Moreover, our dataset was obtained from the CHARGE consortium and PGC consortium, ensuring no overlap in samples. The consistency of effect sizes across different methods, the strength of evidence, and our secondary analyses indicate that our findings are consistent with an effect of fatty acids on ADHD, although the estimate of ALA and EPA are likely to be underpowered, given the small number of instruments used in these two exposures. Our MR-Egger model and MR-PRESSO analyses revealed no outliers, indicating no horizontal pleiotropy, thereby minimizing the potential bias in causal inference. Additionally, our research provides valuable insights for the health management of ADHD patients. While our study does not definitively establish a causal relationship between fatty acids and ADHD, it is crucial to remain vigilant about the risk factors associated with fatty acid deficiency in individuals with ADHD.

Our study also has inevitable limitations. First, the limitation to individuals of European descent, restricting the generalizability of our study to non-European populations. Second, we did not include fatty acid data from other databases, considering that the quality control standards for genome-wide association analysis vary among different databases, and this difference may lead to heterogeneity, which also resulted in a limited number of available instrumental variables for our partial exposure (ALA, EPA) that could not be used for heterogeneity and pleiotropy analysis, or even unavailable (DHA). Third, ADHD has a male predominance, and our data were not stratified by gender, making it impossible to assess the effect of fatty acids on ADHD risk in different genders, potentially introducing bias. Due to the lack of publicly available dataset, our study could not conduct a stratified analysis on the progression and severity of ADHD, as well as different clinical subtypes. Finally, we must pay attention to the diversity of ADHD population and fatty acid types, and in the future, comprehensive research on ADHD subgroups and multiple fatty acids should be considered.

## 5 Conclusion

We found no genetic evidence supporting the causal relationship between n-3 PUFAs, n-6 PUFAs, SFA, and MUFAs in the risk of ADHD. From a public health perspective, our study challenges the notion that supplementing PUFAs can reduce the risk of ADHD. Given the inconsistent evidence from trial data, further MR studies targeting different populations and larger-scale epidemiological research are still needed to validate this conclusion.

## Data availability statement

The original contributions presented in the study are included in the article/**Supplementary Material**. Further inquiries can be directed to the corresponding author.

## Ethics statement

All data are publicly available and are approved by the institutional review committees in their respective studies. Therefore, no further sanction was required. The studies were conducted in accordance with the local legislation and institutional requirements. The participants provided their written informed consent to participate in this study.

## Author contributions

KZ: Conceptualization, Data curation, Formal analysis, Investigation, Methodology, Software, Writing – original draft, Writing – review & editing. QZhang: Investigation, Visualization, Writing – original draft. ZY: Validation, Visualization, Writing – review & editing. YY: Formal analysis, Investigation, Writing – original draft. QZhao: Project administration, Supervision, Writing – review & editing. JW: Funding acquisition, Project administration, Resources, Writing – review & editing.
