# Supplementary material for: Plasma fatty acids and attention deficit hyperactivity disorder: a Mendelian randomization investigation
Source: Front Psychiatry. 2024 May 3;15:1368942. doi: 10.3389/fpsyt.2024.1368942 (PMC11099612; doi:10.3389/fpsyt.2024.1368942)
Supplement: Supplementary file 1 [file DataSheet_1.pdf]

Supplementary Material

Plasma fatty acids and attention deficit hyperactivity disorder: a Mendelian randomization investigation

Kangning Zhou*, Qiang Zhang, Zhenhua Yuan, Yurou Yan, Qiang He, Junhong Wang

*** Correspondence:** drjhwang@bucm.edu.cn

Content

1 Supplementary Figures and Tables 1

1.1 Supplementary Figures 1

Figure S1. Leave-one-out graph for SFAs and MUFAs on ADHD. 2

Figure S2. Leave-one-out graph for n-3 PUFAs on ADHD. 3

Figure S3. Leave-one-out graph for n-6 PUFAs on ADHD. 5

Figure S4. Forest plot for SFAs and MUFAs on ADHD. 7

Figure S5. Forest plot for n-3 PUFAs on ADHD. 8

Figure S6. Forest plot for n-6 PUFAs on ADHD. 9

Figure S7. Funnel plot for SFAs and MUFAs on ADHD. 11

Figure S8. Funnel plot for n-3 PUFAs on ADHD. 12

Figure S9. Funnel plot for n-6 PUFAs on ADHD. 13

Figure S10. Leave-one-out graph for ADHD on SFAs and MUFAs. 15

Figure S11. Leave-one-out graph for ADHD on n-3 PUFAs. 17

Figure S12. Leave-one-out graph for ADHD on n-6 PUFAs. 19

Figure S13. Forest plot for ADHD on SFAs and MUFAs. 21

Figure S14. Forest plot for ADHD on n-3 PUFAs. 23

Figure S15. Forest plot for ADHD on n-6 PUFAs. 24

Figure S16. Funnel plot for ADHD on SFAs and MUFAs. 26

Figure S17. Funnel plot for ADHD on n-3 PUFAs. 28

Figure S18. Funnel plot for ADHD on n-6 PUFAs. 30

Figure S19. Scatter plots for ADHD on SFA and MUFA. 32

Figure S20. Scatter plots for ADHD on n-3PUFA. 34

Figure S21. Scatter plots for ADHD on n-6PUFA. 35
1.2 Supplementary Tables 36
Table S1. 4 valid IVs used for MR analysis of SFA 16:0 on ADHD 36
Table S2. 4 valid IVs used for MR analysis of SFA 18:0 on ADHD 36
Table S3. 5 valid IVs used for MR analysis of MUFA 16:1n7 on ADHD 37
Table S4. 3 valid IVs used for MR analysis of MUFA 18:1n9 on ADHD 37
Table S5. 2 valid IVs used for MR analysis of n-3 ALA on ADHD 37
Table S6. 1 valid IVs used for MR analysis of n-3 EPA on ADHD 38
Table S7. 5 valid IVs used for MR analysis of n-3 DPA on ADHD 38
Table S8. 5 valid IVs used for MR analysis of n-6 Adrenic A on ADHD....................................39
Table S9. 12 valid IVs used for MR analysis of n-6 DGLA on ADHD 40
Table S10. 48 valid IVs used for MR analysis of n-6 GLA on ADHD 41
Table S11. 13 valid IVs used for MR analysis of ADHD on SFA, MUFA, n-3 DPA, n-3 .EPA, n-6DGLA, n-6GLA…………………………………………………………………..……44
Table S12. 12 valid IVs used for MR analysis of ADHD on n-3ALA 45
Table S13. 11 valid IVs used for MR analysis of ADHD on n-6 Adrenic acid 46

1 Supplementary Figures and Tables

1.1 Supplementary Figures

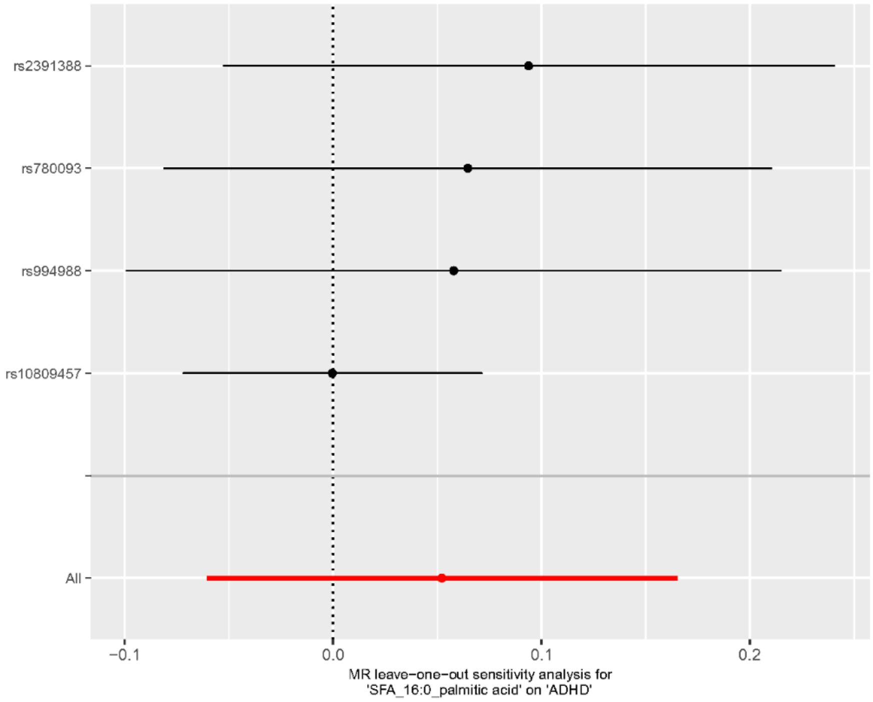

Figure S1a.

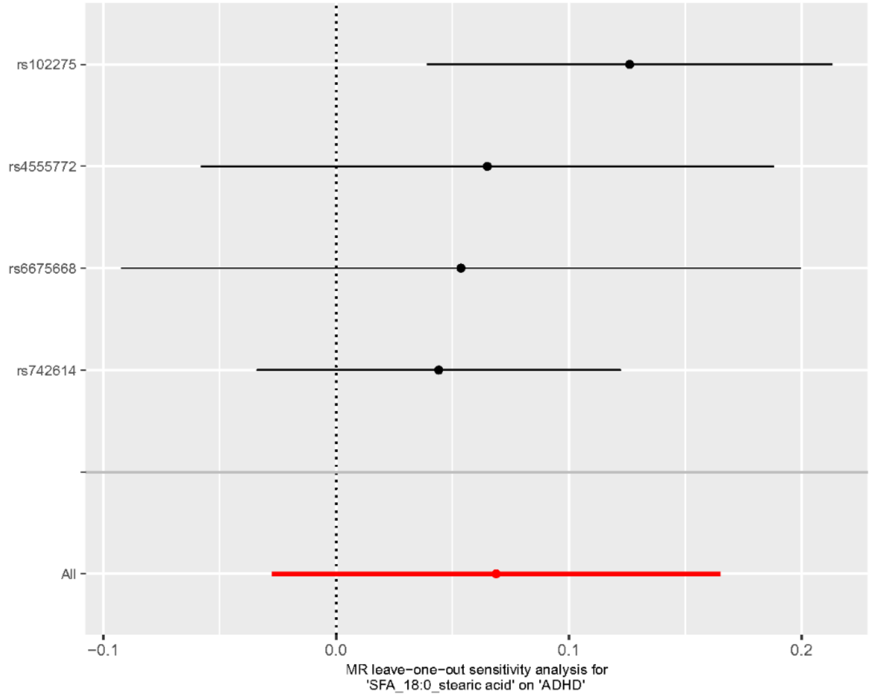

Figure S2b.

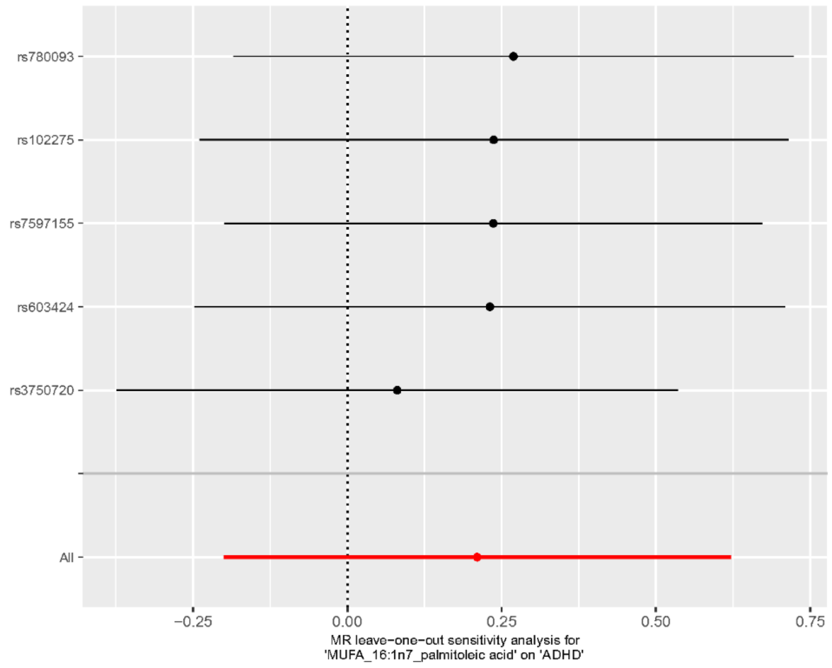

Figure S3c.

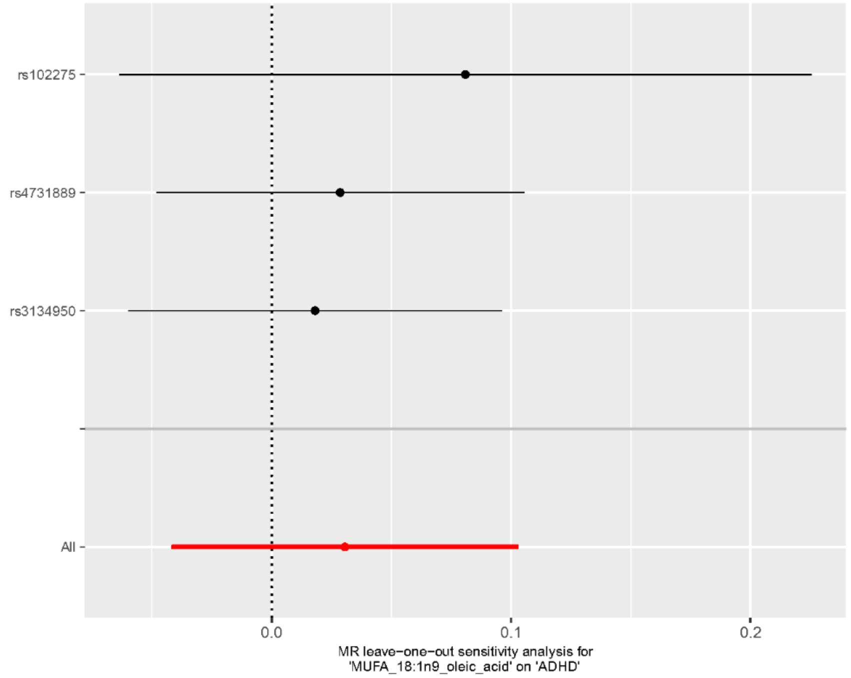

Figure S4d.

Figure S1. Leave-one-out graph for SFAs and MUFAs on ADHD.

(A) Leave-one-out graph for SFA 16:0 on ADHD. (B) Leave-one-out graph for SFA 18:0 on ADHD. (C) Leave-one-out graph for MUFA 16:1n7 on ADHD (D) Leave-one-out graph for MUFA 18:1n9 on ADHD.

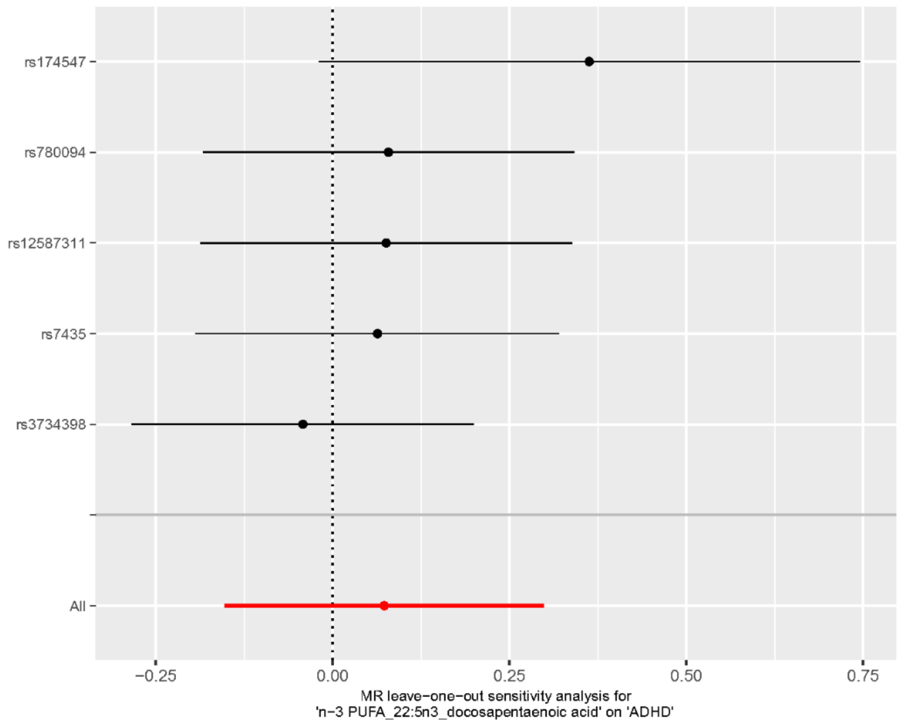

Figure S2a.

Figure S2. Leave-one-out graph for n-3 PUFAs on ADHD.

(A) Leave-one-out graph for n-3 DPA on ADHD.

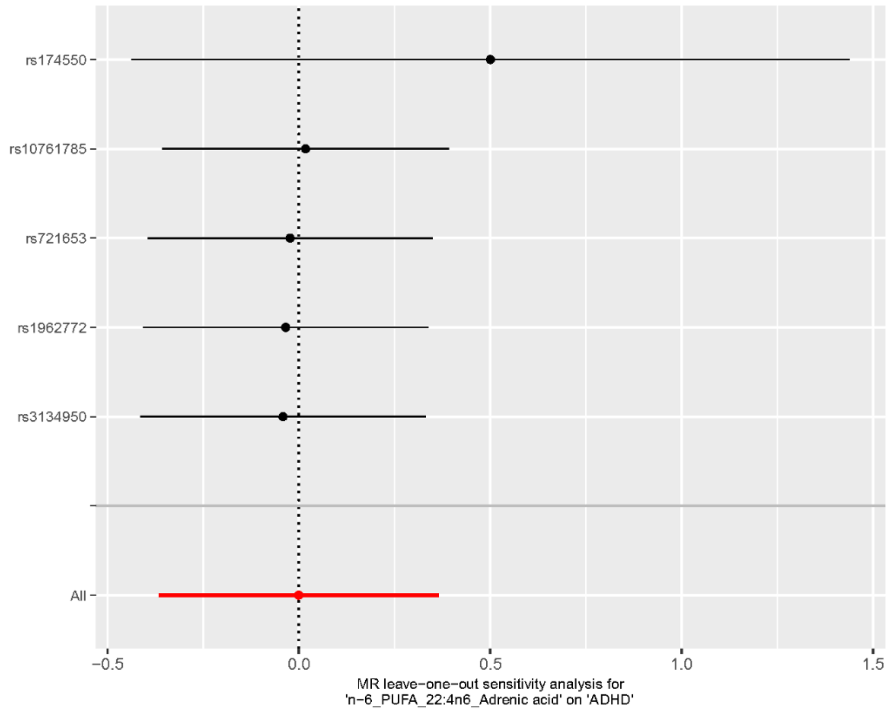

Figure S3a.

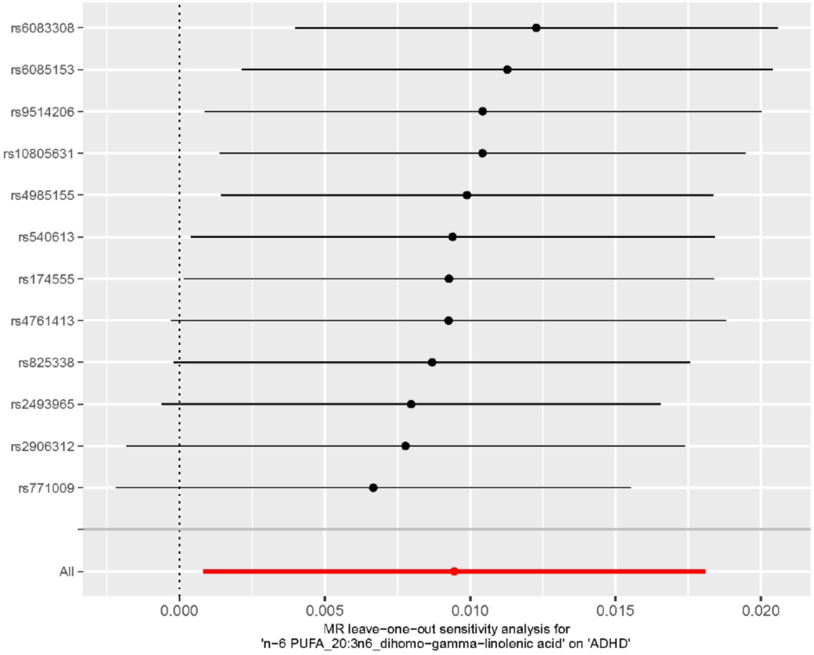

Figure S3b.

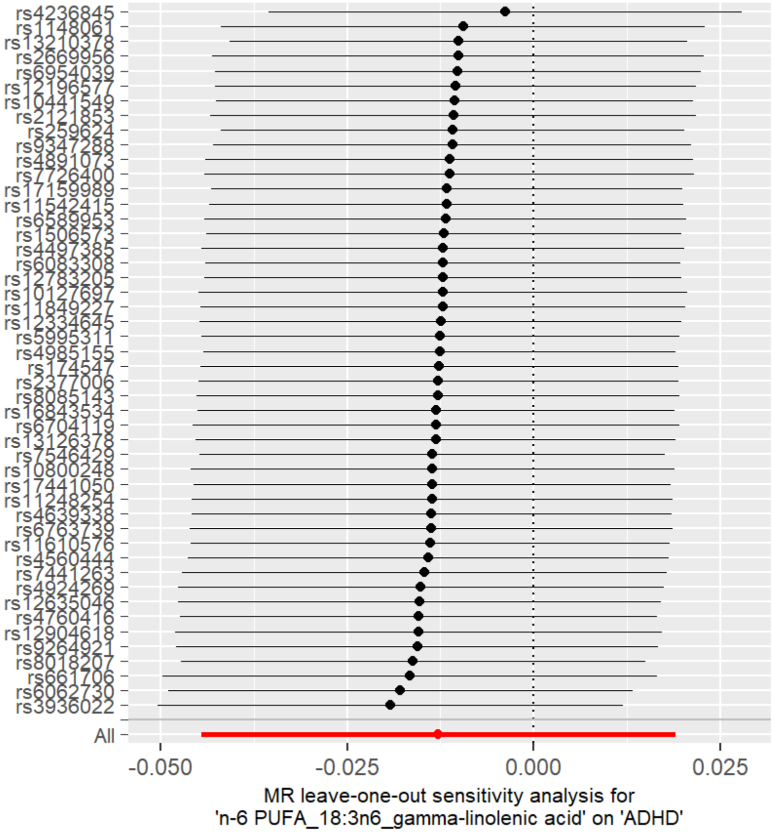

Figure S3c.

Figure S3. Leave-one-out graph for n-6 PUFAs on ADHD.

(A) Leave-one-out graph for n-6 Adrenic A on ADHD. (B) Leave-one-out graph for n-6 DGLA on ADHD. (C) Leave-one-out graph for n-6 GLA on ADHD.

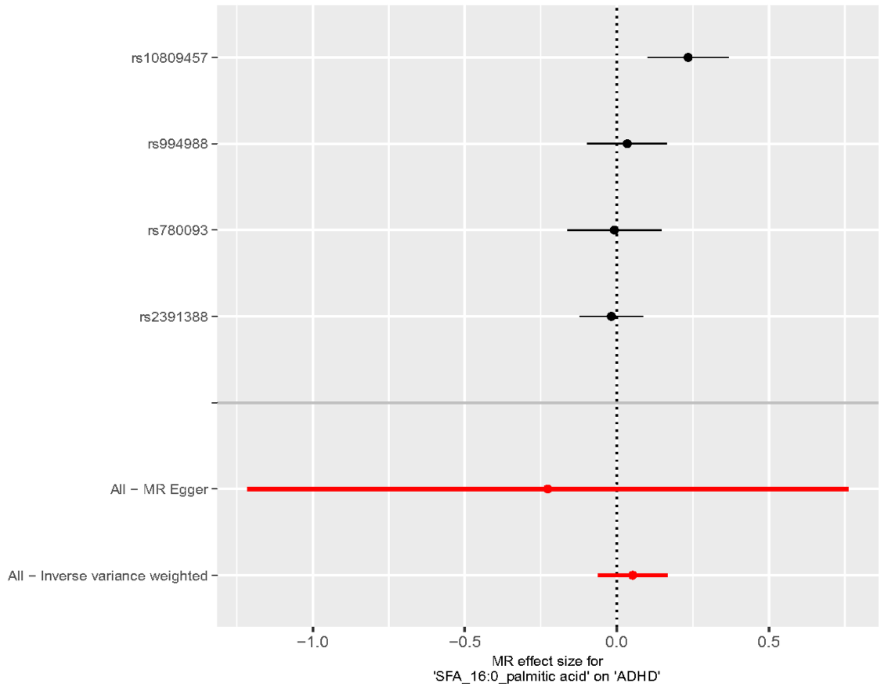

Figure S4a.

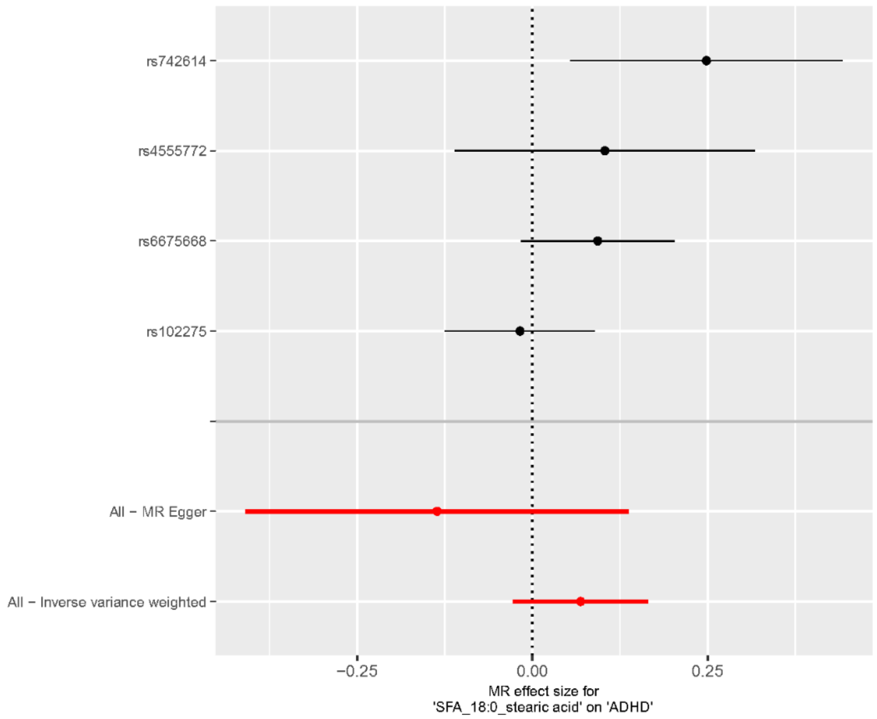

Figure S4b.

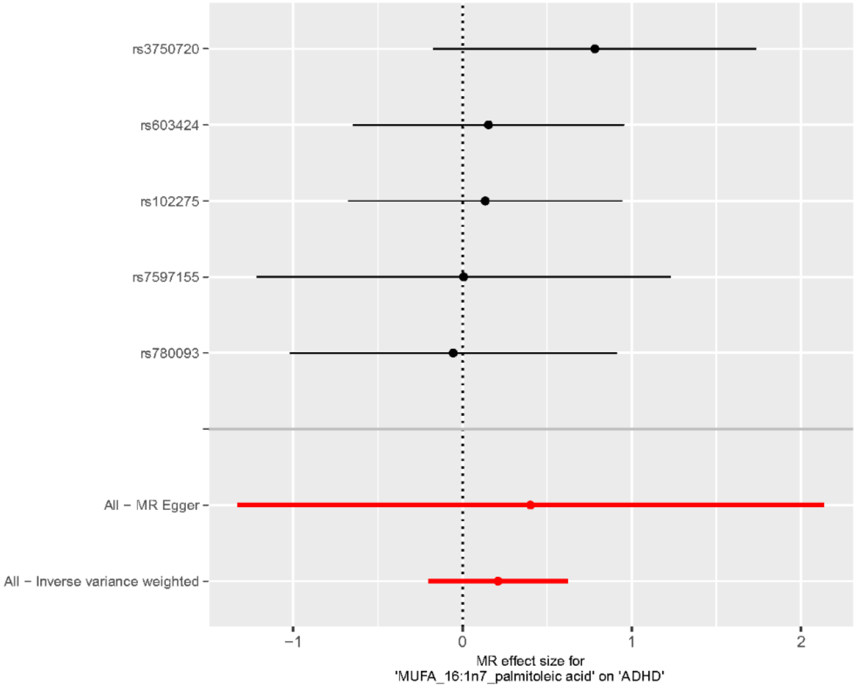

Figure S4c.

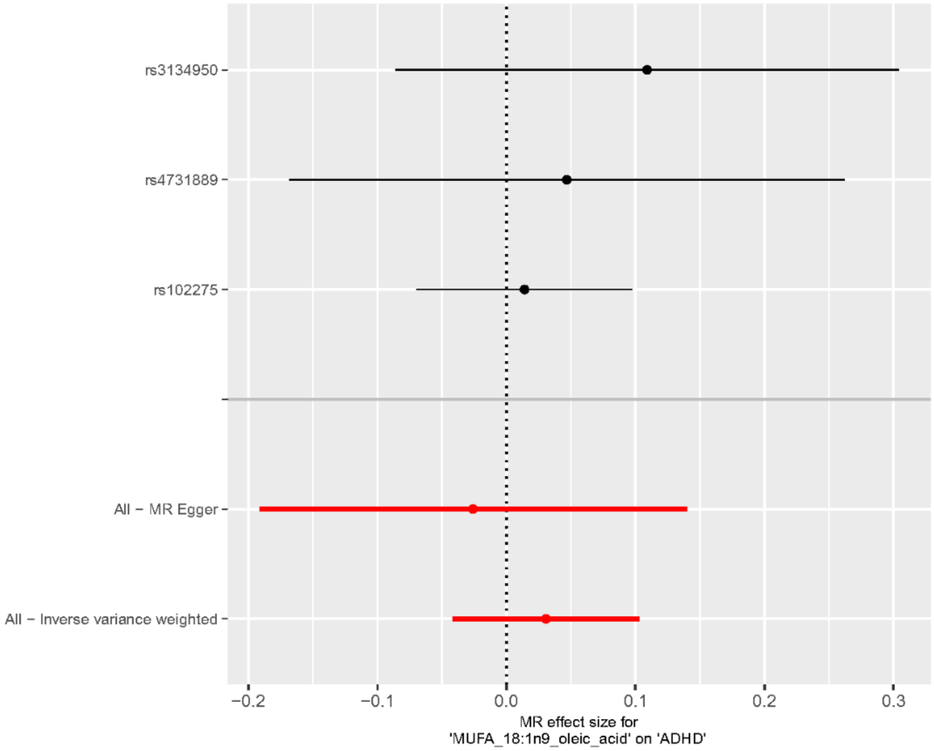

Figure S4d.

Figure S4. Forest plot for SFAs and MUFAs on ADHD.

(A) Forest plot for SFA 16:0 on ADHD. (B) Forest plot for SFA 16:1n7 on ADHD. (C) Forest plot for MUFA 18:0 on ADHD (D) Forest plot for MUFA 18:1n9 on ADHD.

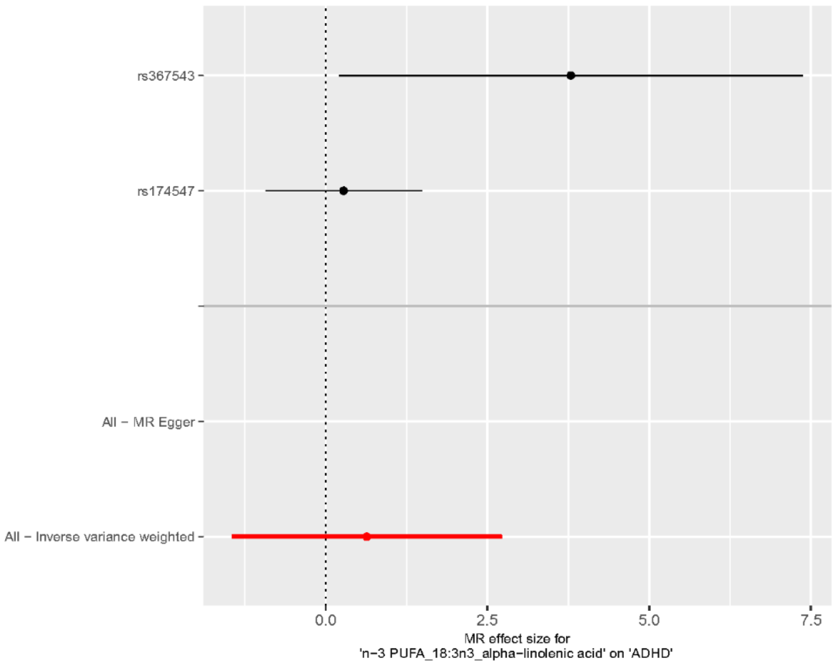

Figure S5a.

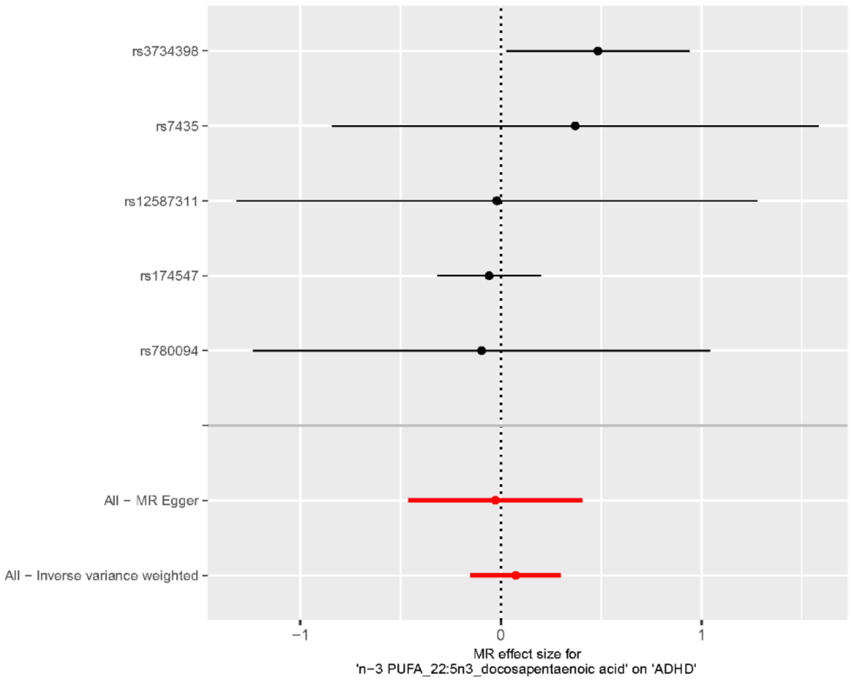

Figure S5b.

Figure S5. Forest plot for n-3 PUFAs on ADHD.

(A) Forest plot for ALA on ADHD. (B) Forest plot for DPA on ADHD.

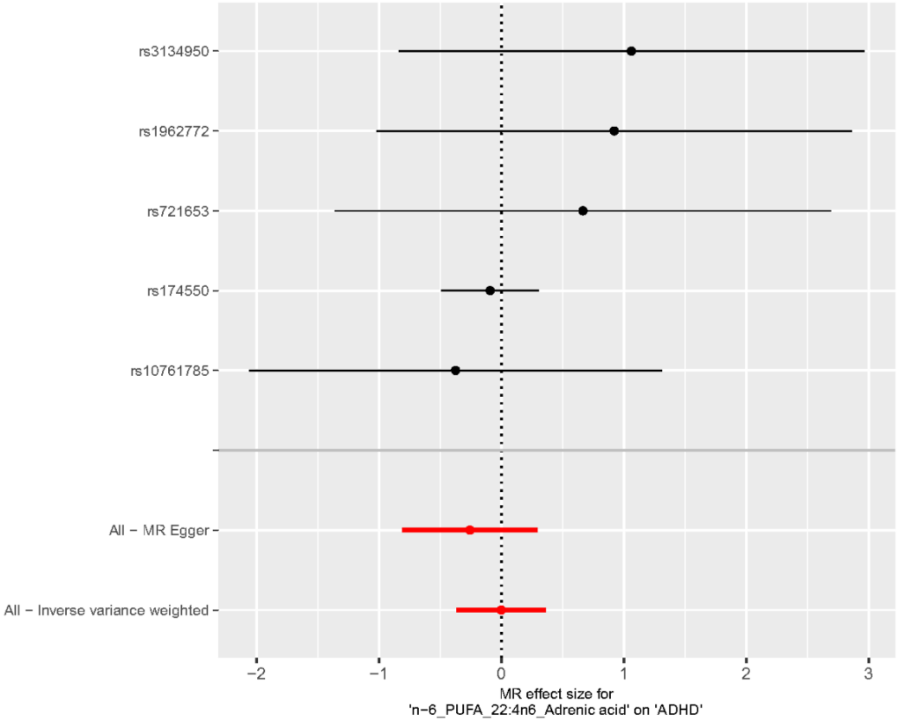

Figure S6a.

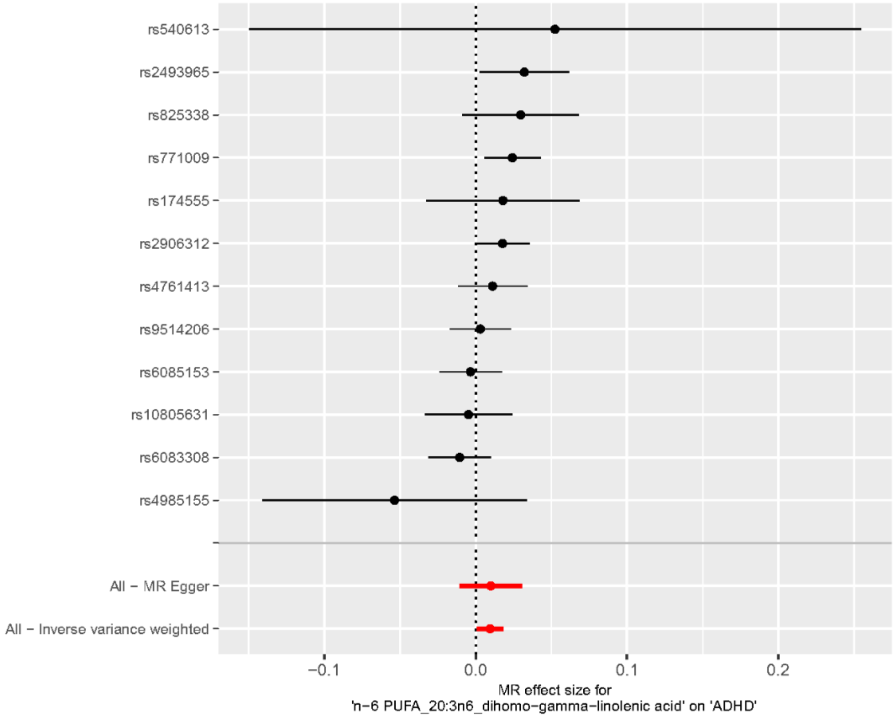

Figure S6b.

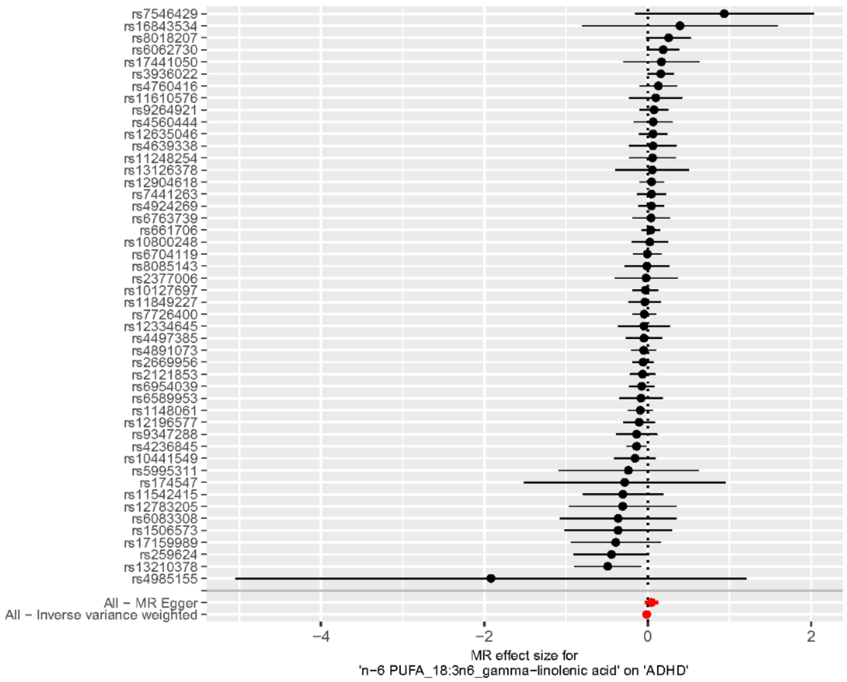

Figure S6c.

Figure S6. Forest plot for n-6 PUFAs on ADHD.

(A) Forest plot for n-6 Adrenic A on ADHD. (B) Forest plot for n-6 DGLA on ADHD. (C) Forest plot for n-6 GLA on ADHD.

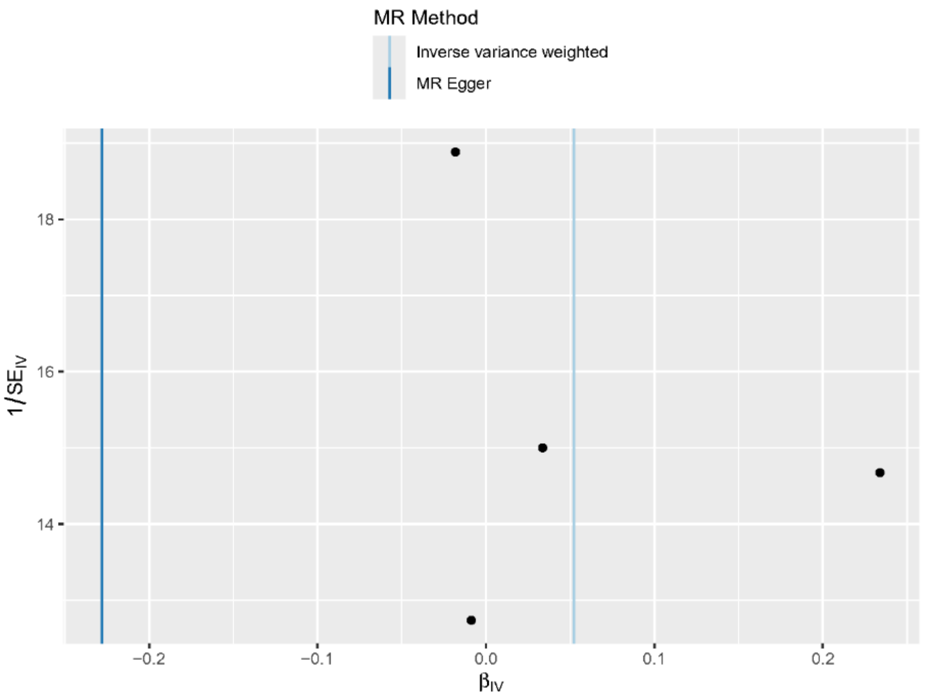

Figure S7a.

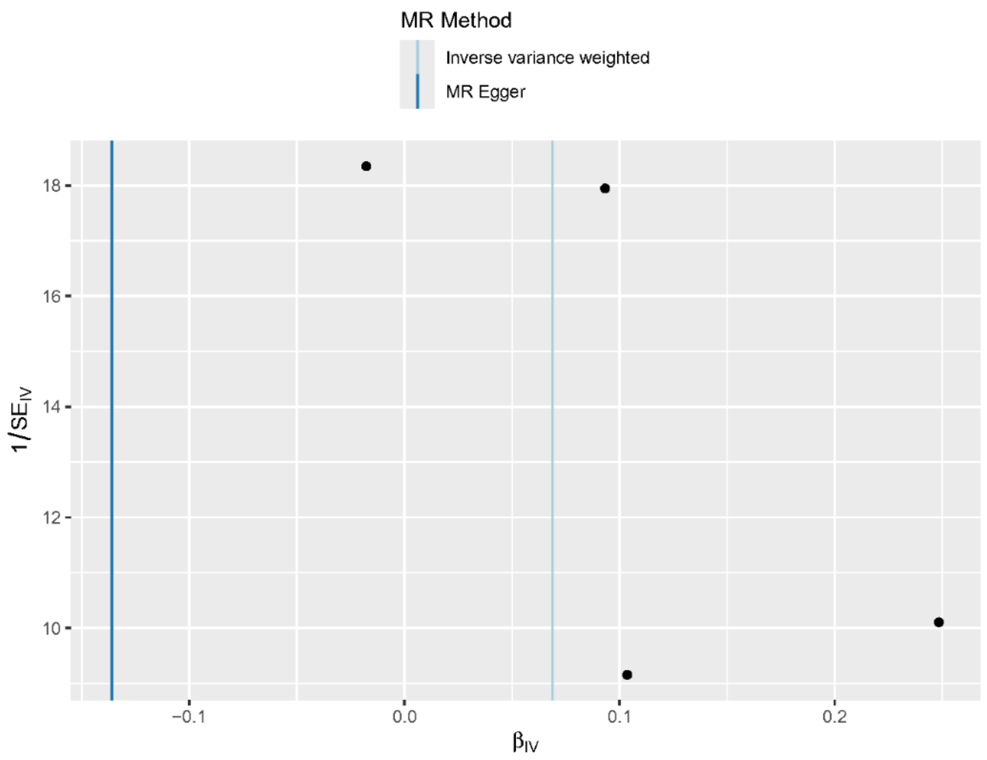

Figure S7b.

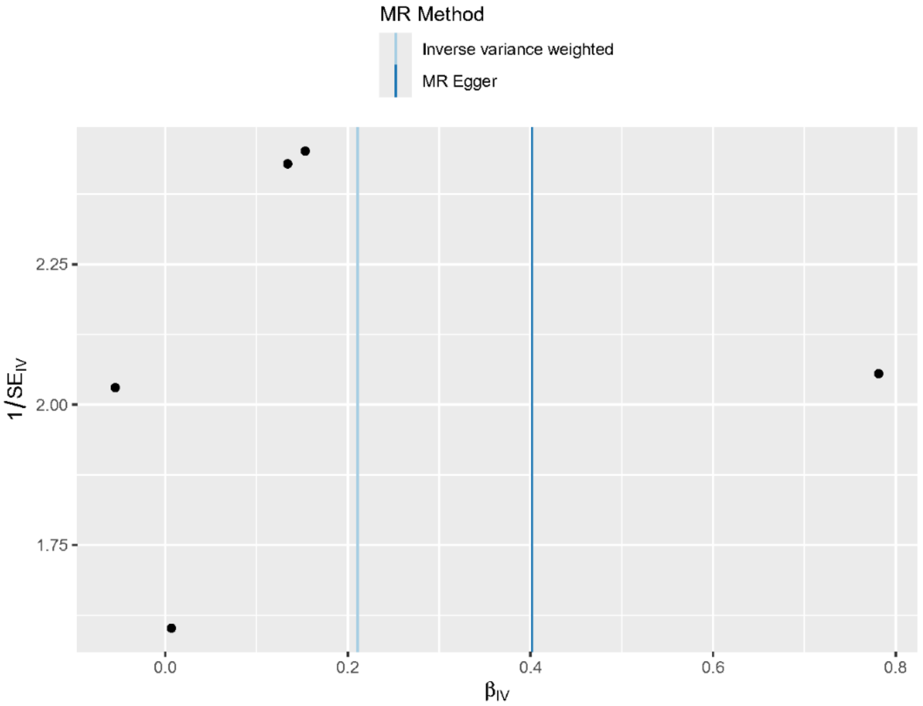

Figure S7c.

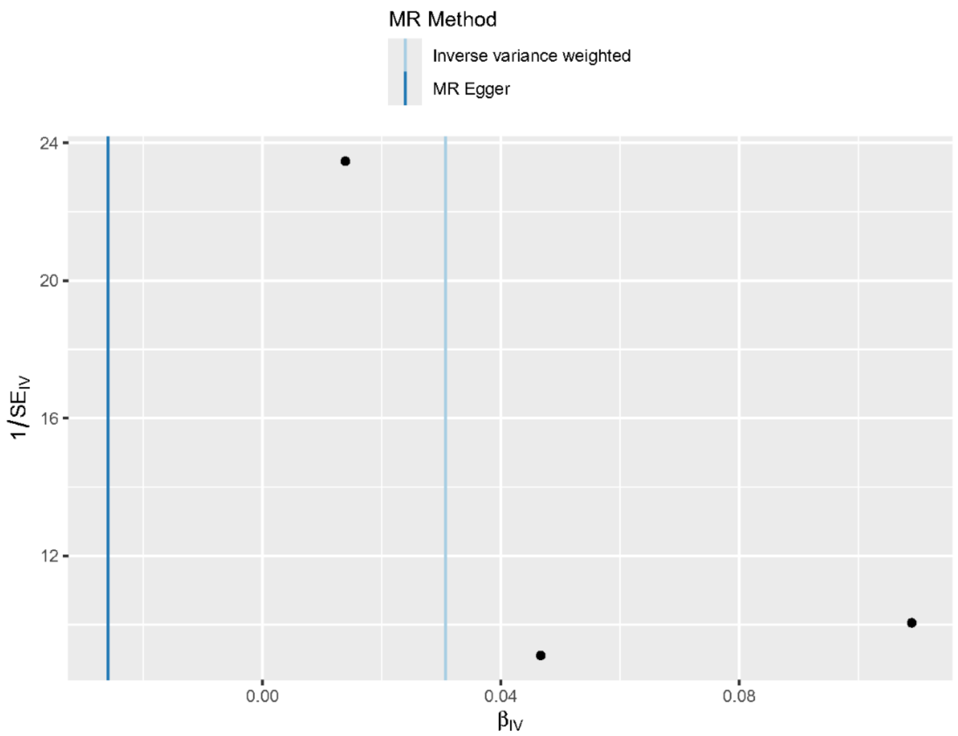

Figure S7d.

Figure S7. Funnel plot for SFAs and MUFAs on ADHD.

(A) Funnel plot for SFA 16:0 on ADHD. (B) Funnel plot for SFA 16:1n7 on ADHD. (C) Funnel plot for MUFA 18:0 on ADHD (D) Funnel plot for MUFA 18:1n9 on ADHD.

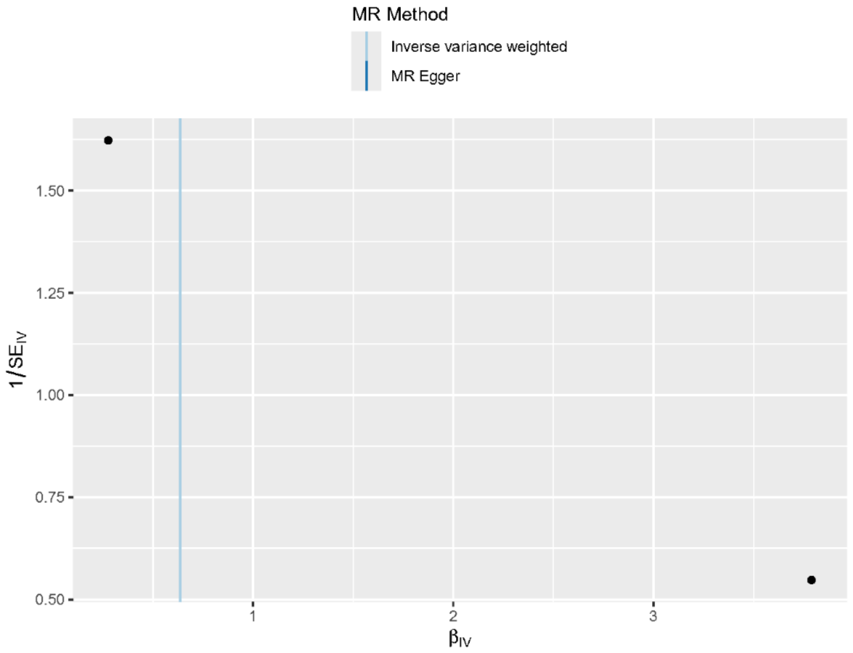

Figure S8a.

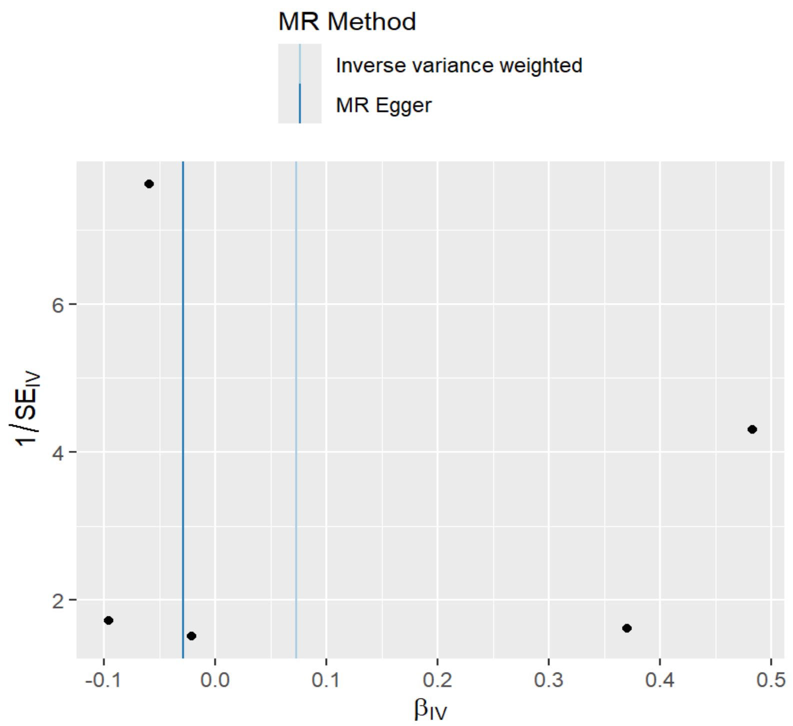

Figure S8b.

Figure S8. Funnel plot for n-3 PUFAs on ADHD.

(A) Funnel plot for ALA on ADHD. (B) Funnel plot for DPA on ADHD.

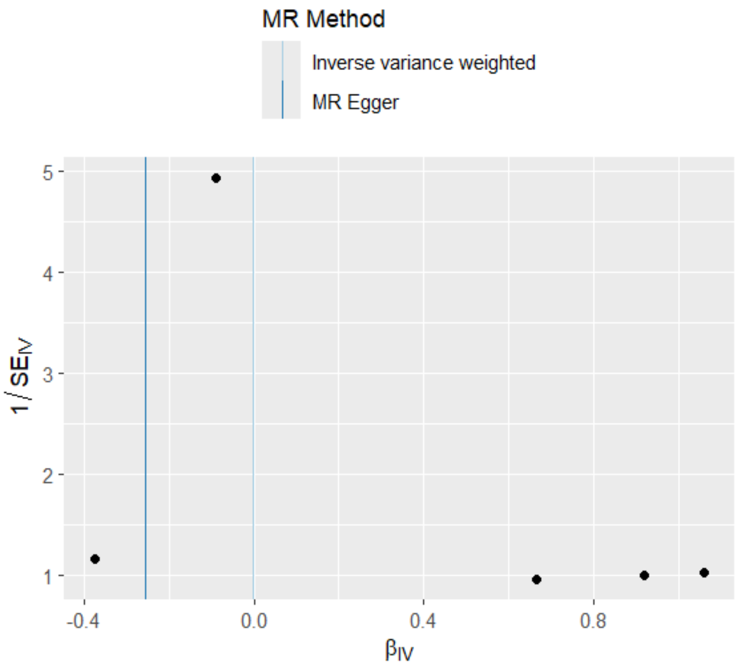

Figure S9a.

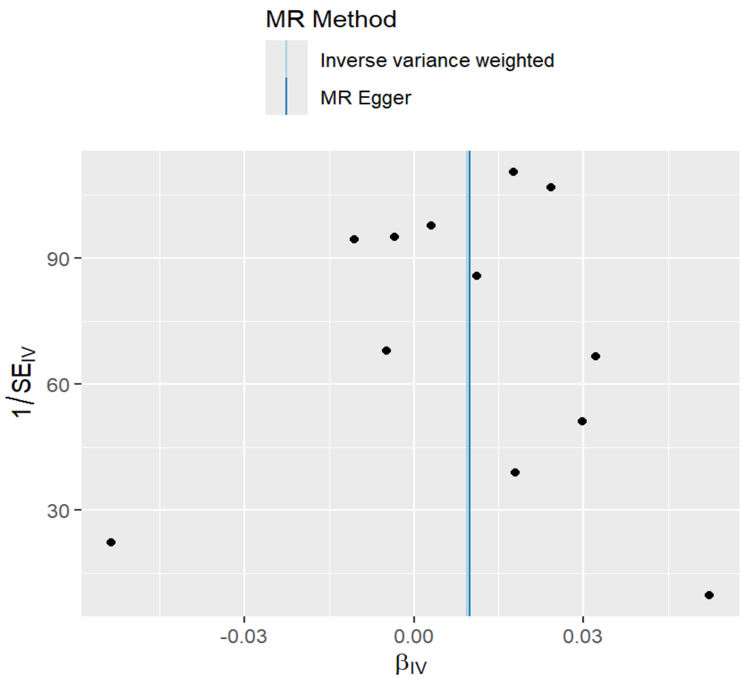

Figure S9b.

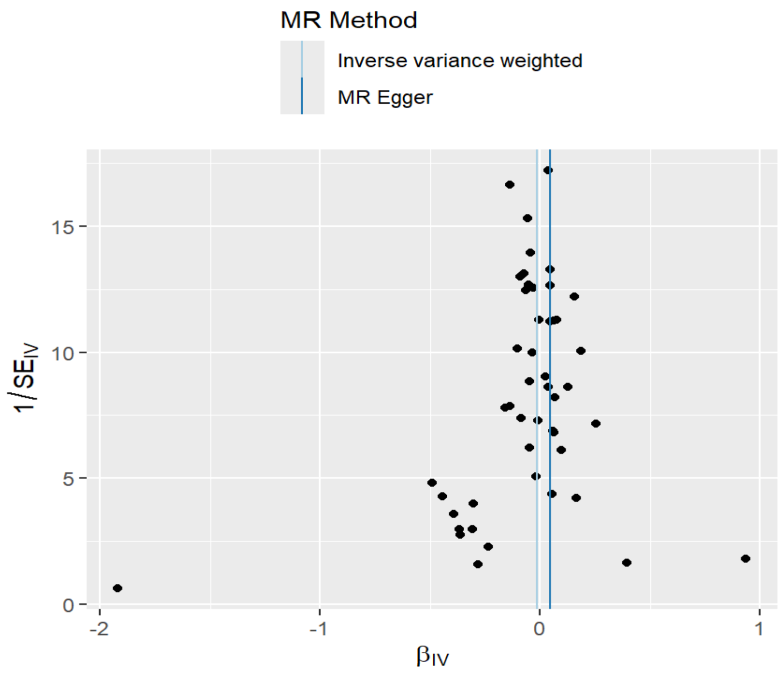

Figure S9c.

Figure S9. Funnel plot for n-6 PUFAs on ADHD.

(A) Funnel plot for n-6 Adrenic A on ADHD. (B) Funnel plot for n-6 DGLA on ADHD. (C) Funnel plot for n-6 GLA on ADHD.

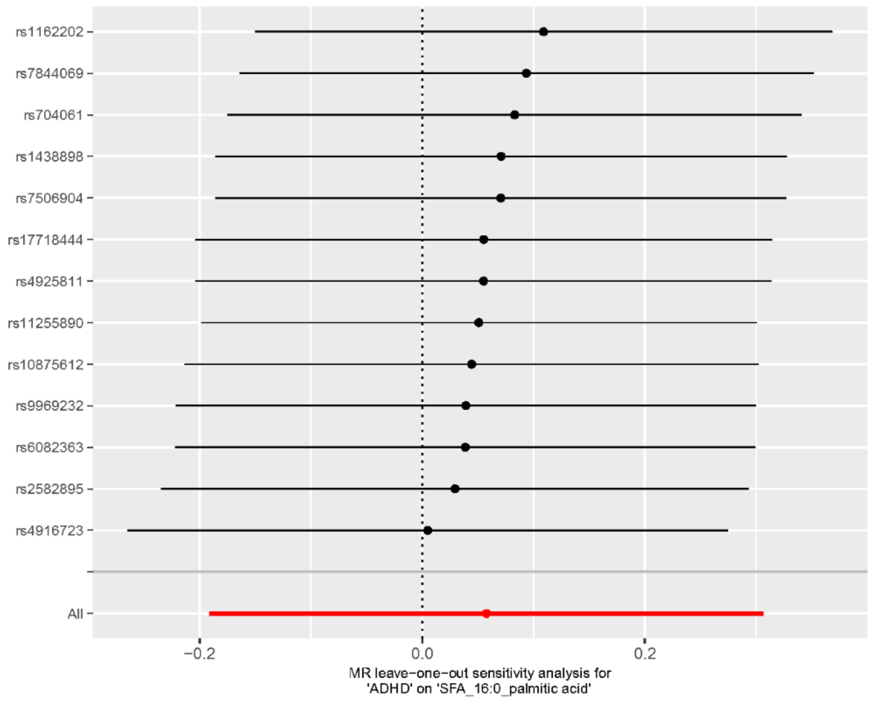

Figure S50a.

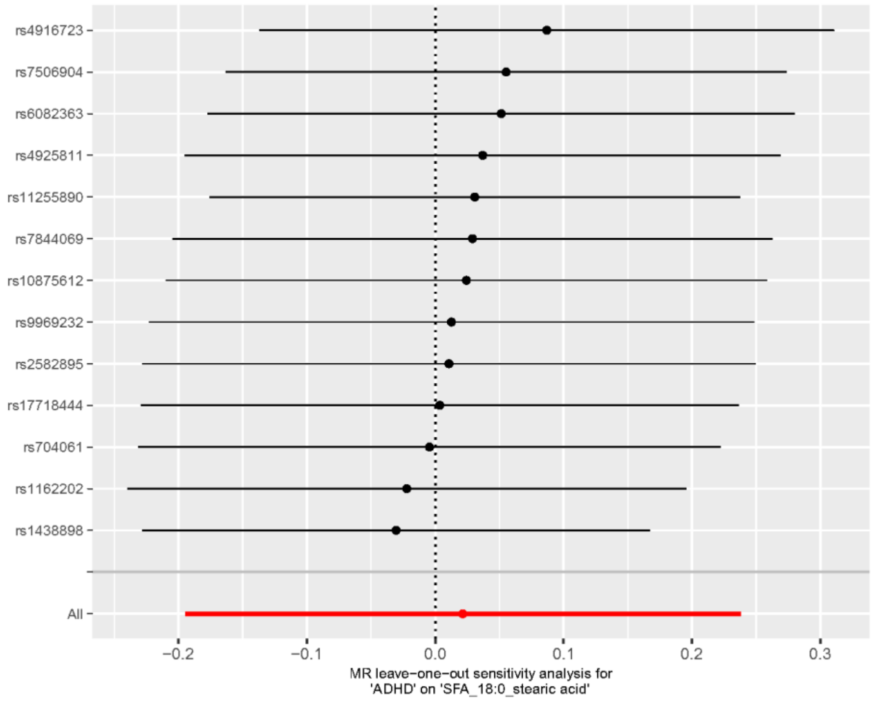

Figure S60b.

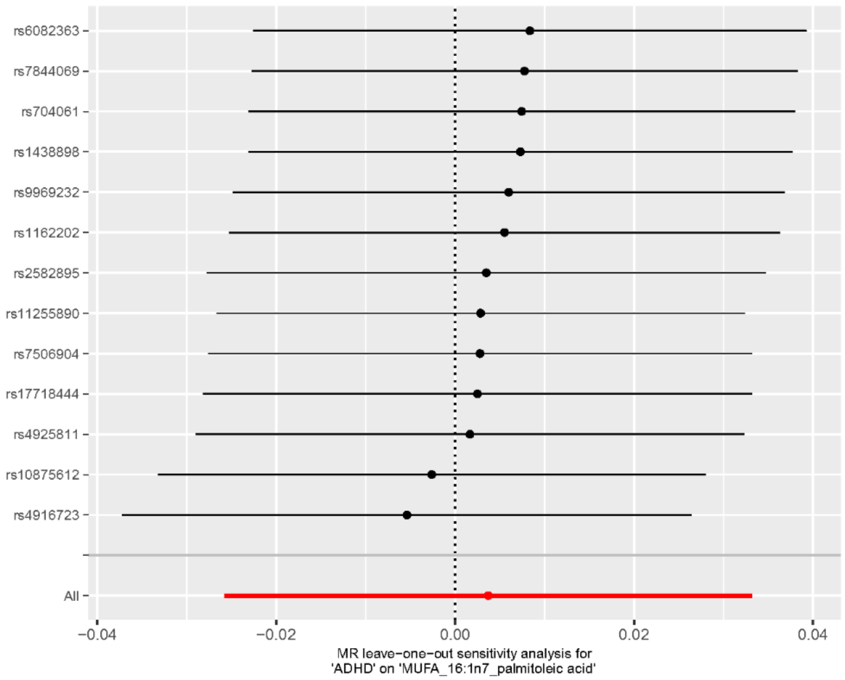

Figure S70c.

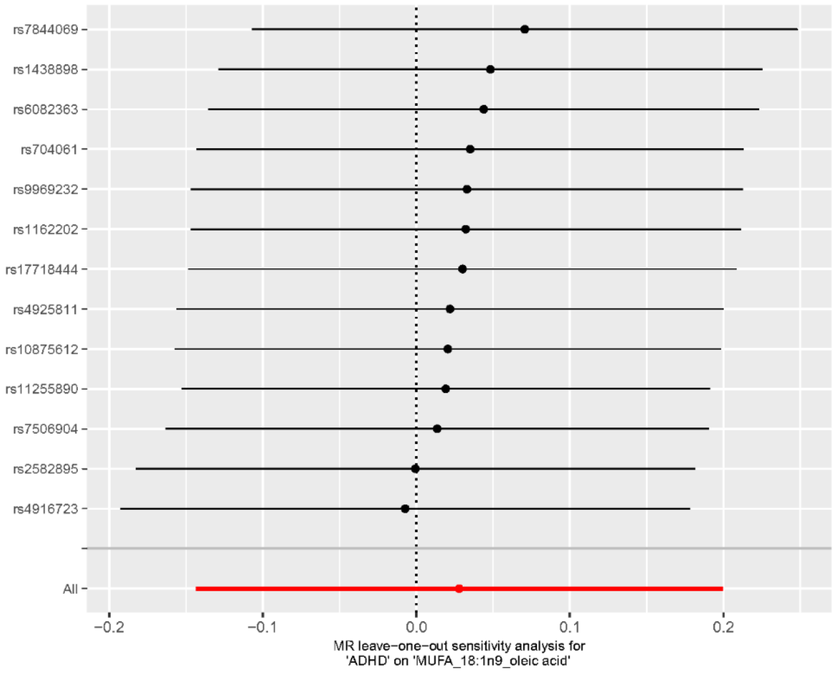

Figure S80d.

Figure S10. Leave-one-out graph for ADHD on SFAs and MUFAs.

(A) Leave-one-out graph for SFA 16:0 on ADHD. (B) Leave-one-out graph for SFA 18:0 on ADHD. (C) Leave-one-out graph for MUFA 16:1n7 on ADHD. (D) Leave-one-out graph for MUFA 18:1n9 on ADHD.

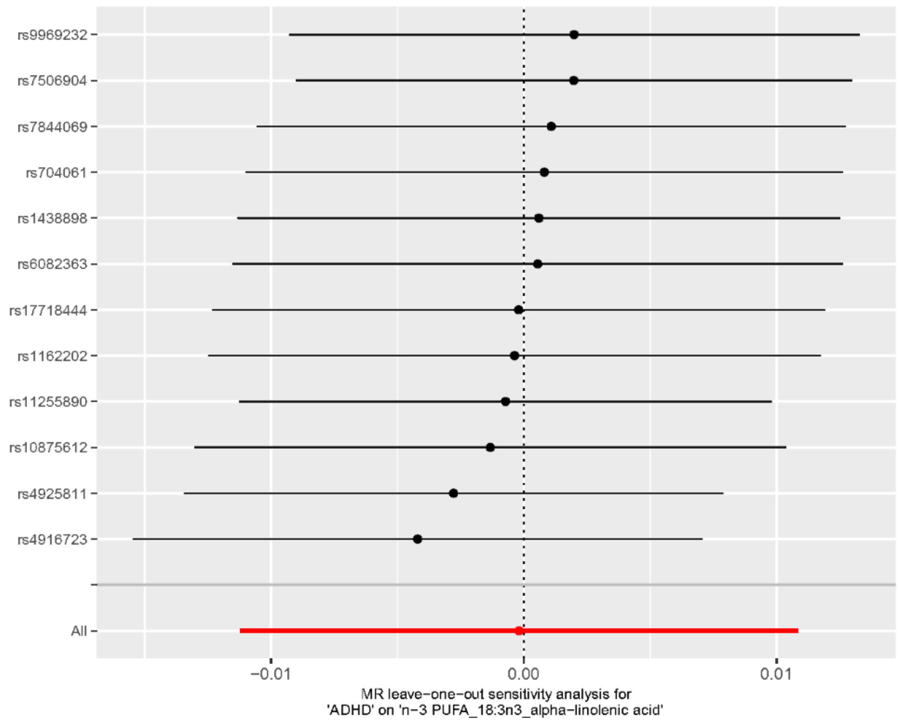

Figure S11a.

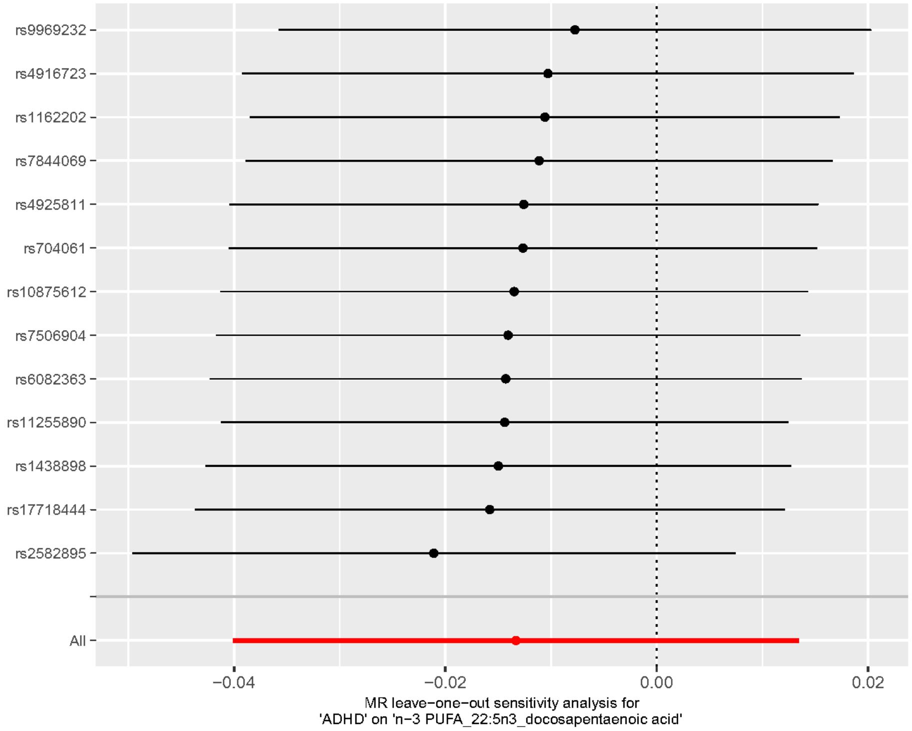

Figure S11b.

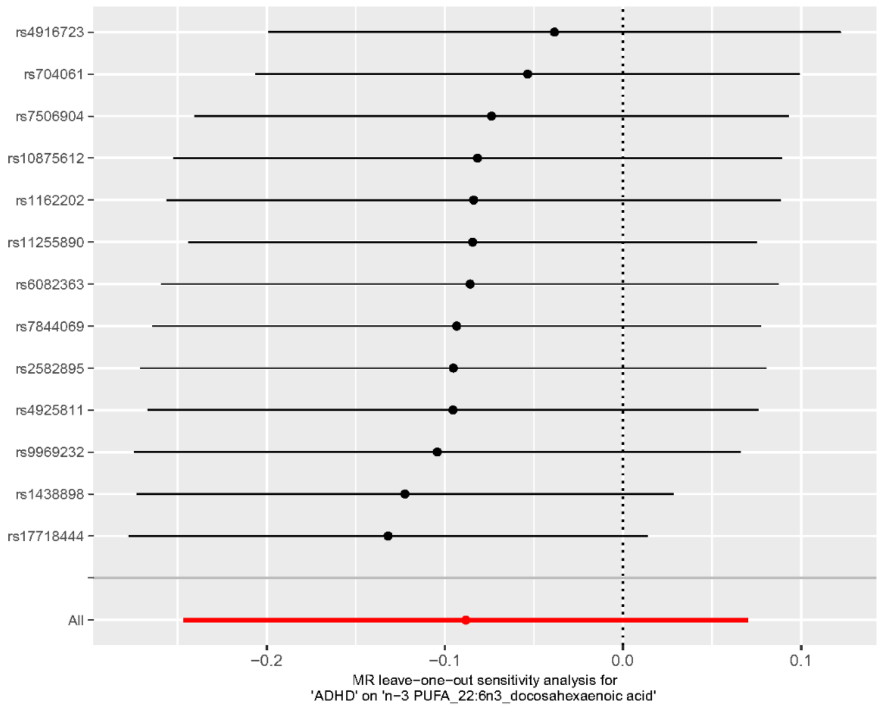

Figure S11c.

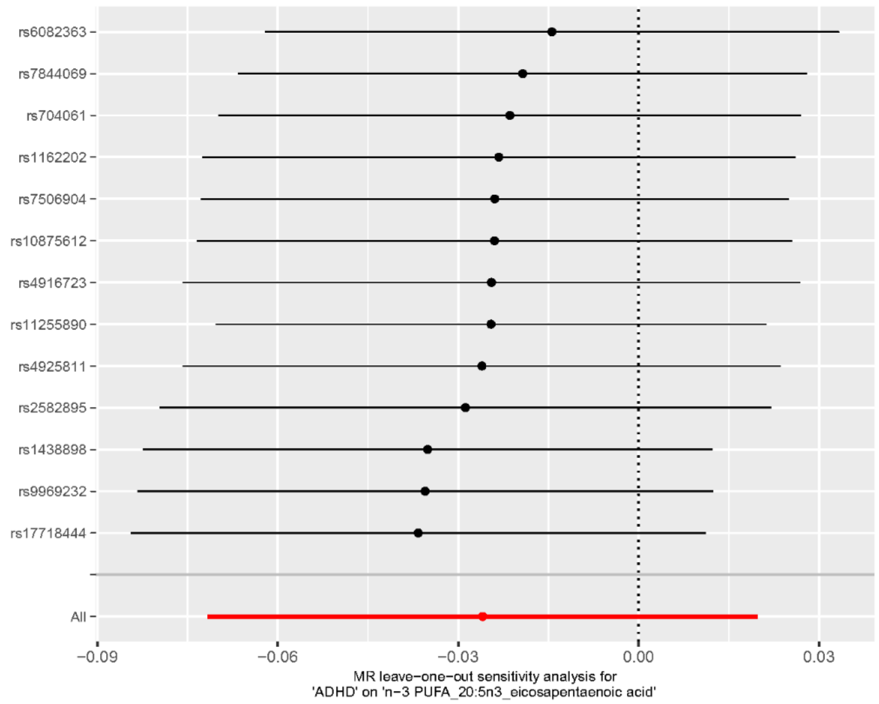

Figure S11d.

Figure S11. Leave-one-out graph for ADHD on n-3 PUFAs.

(A) Leave-one-out graph for n-3 ALA on ADHD. (B) Leave-one-out graph for n-3 DPA on ADHD. (C) Leave-one-out graph for n-3 DHA on ADHD. (D) Leave-one-out graph for n-3 EPA on ADHD.

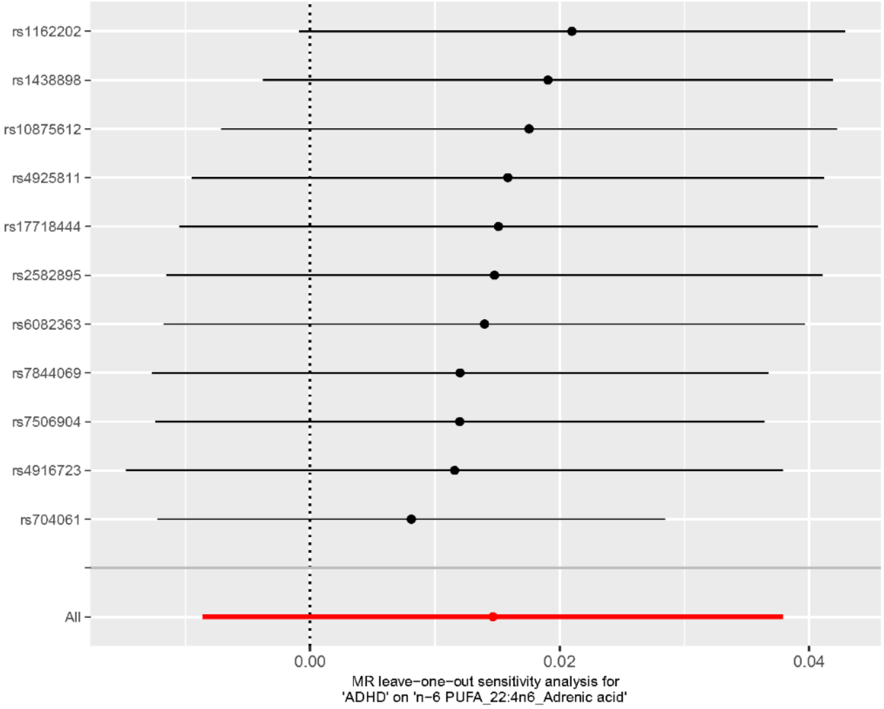

Figure S12a.

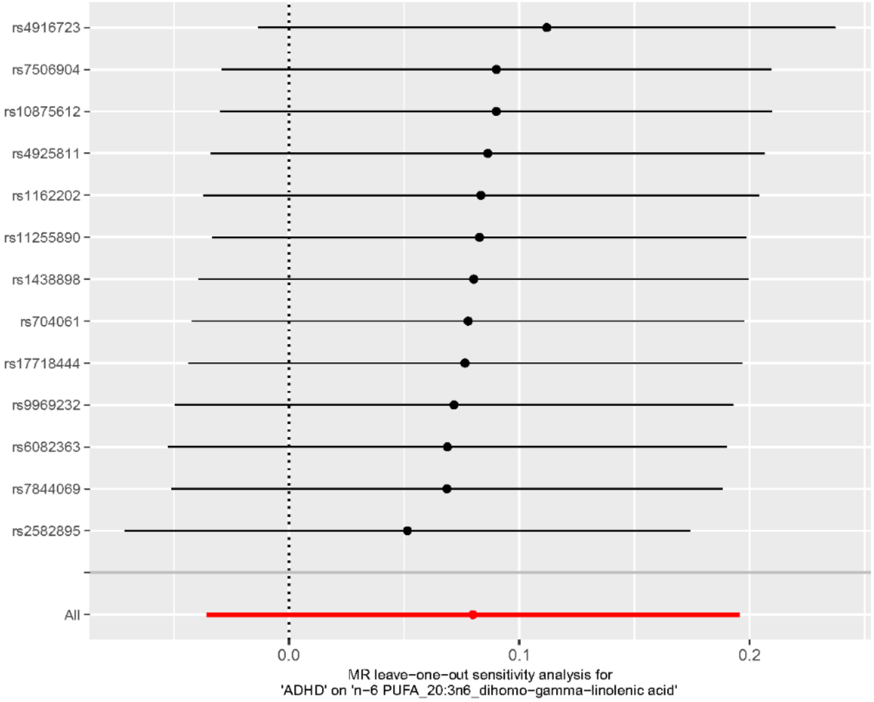

Figure S12b.

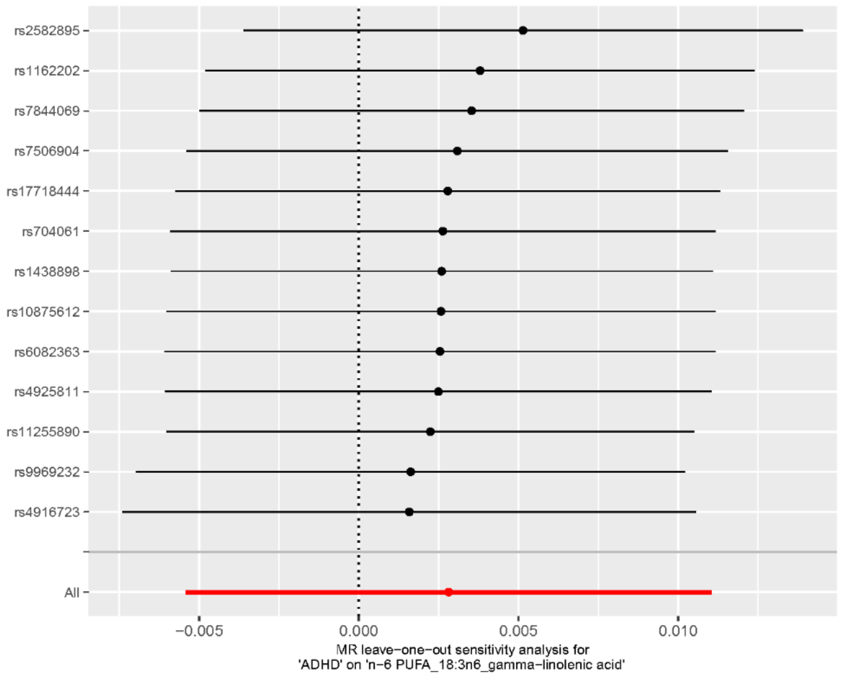

Figure S12c.

Figure S12. Leave-one-out graph for ADHD on n-6 PUFAs.

(A) Leave-one-out graph for n-6 Adrenic A on ADHD. (B) Leave-one-out graph for n-6 DGLA on ADHD. (C) Leave-one-out graph for n-6 GLA on ADHD.

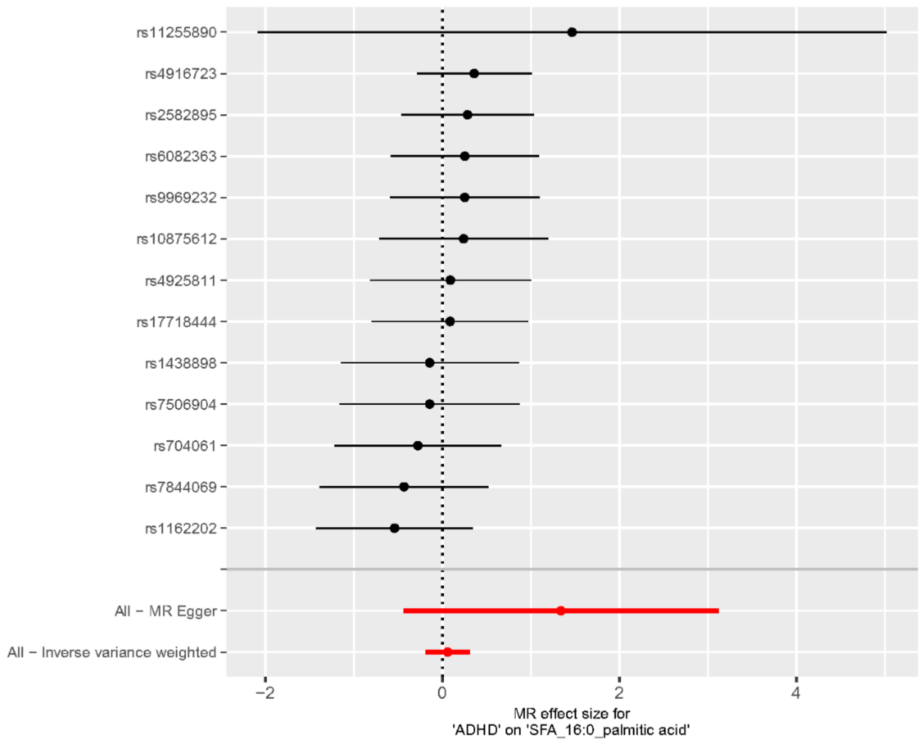

Figure S13a.

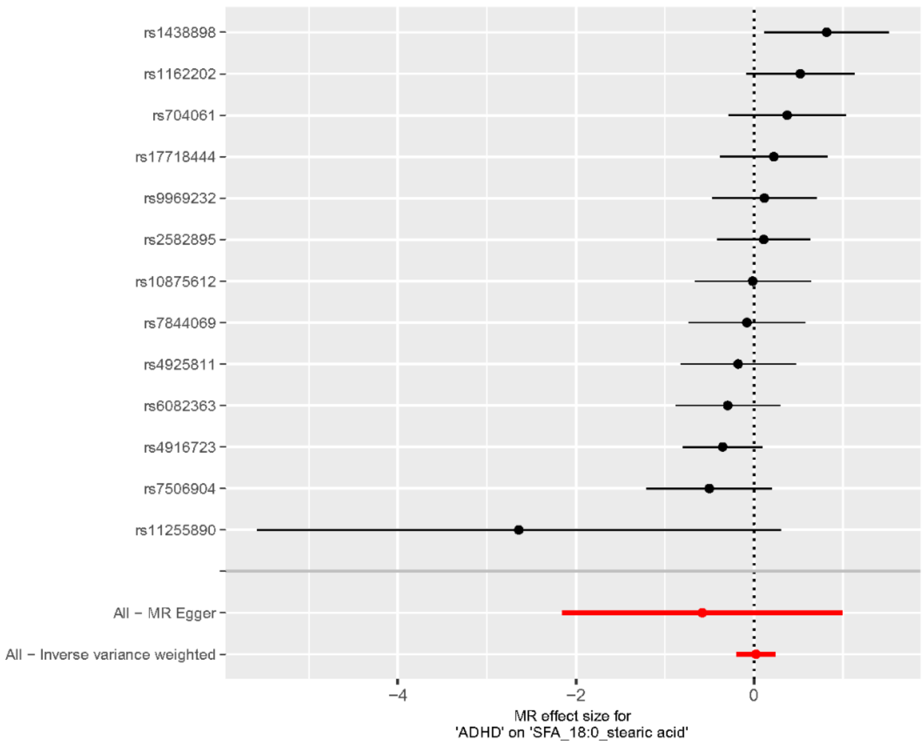

Figure S13b.

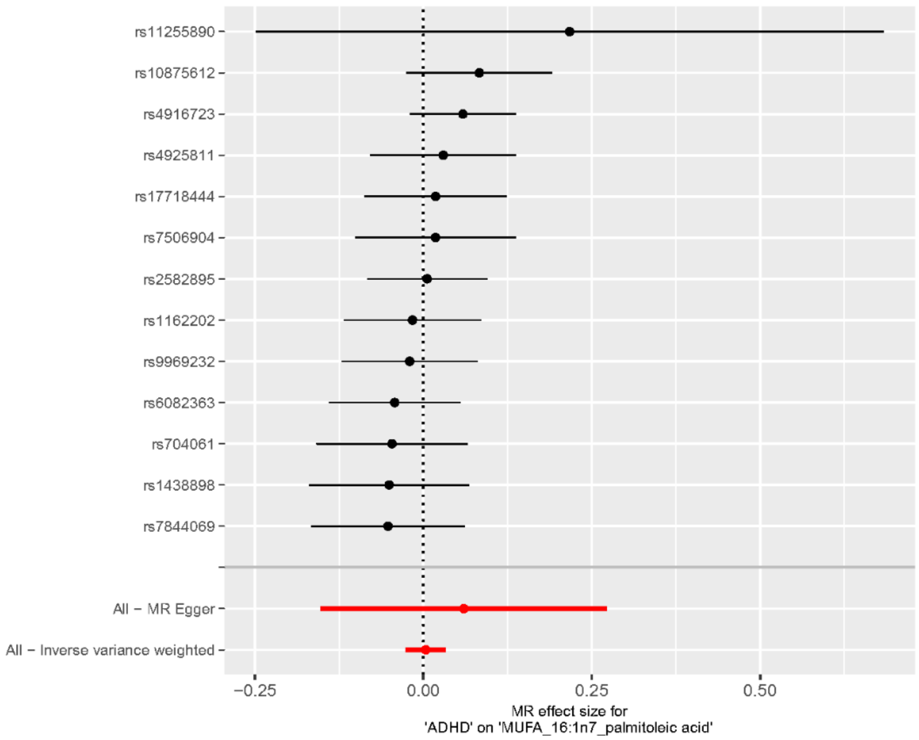

Figure S13c.

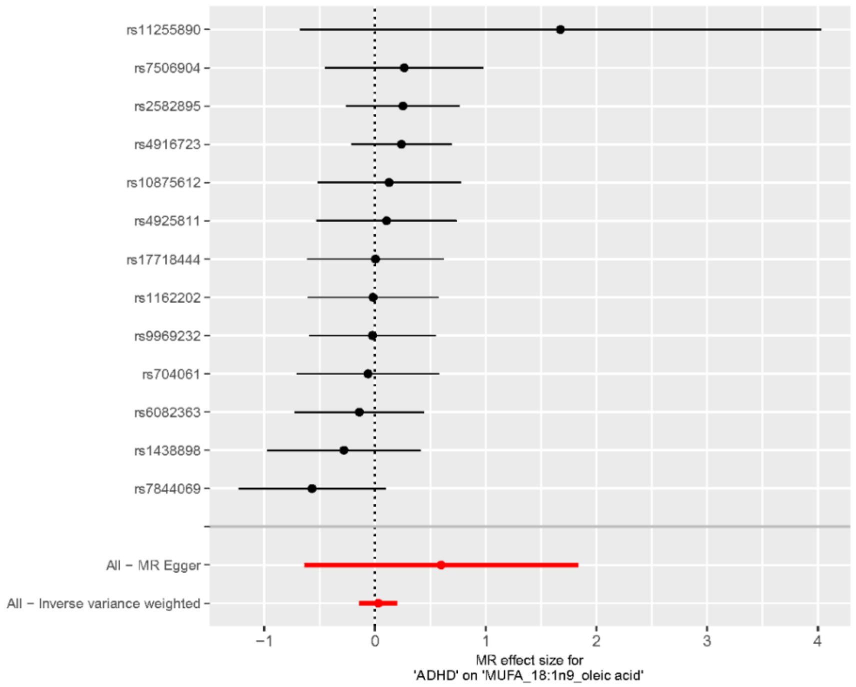

Figure S13d.

Figure S13. Forest plot for ADHD on SFAs and MUFAs.

(A) Forest plot for SFA 16:0 on ADHD. (B) Forest plot for SFA 16:1n7 on ADHD. (C) Forest plot for MUFA 18:0 on ADHD (D) Forest plot for MUFA 18:1n9 on ADHD.

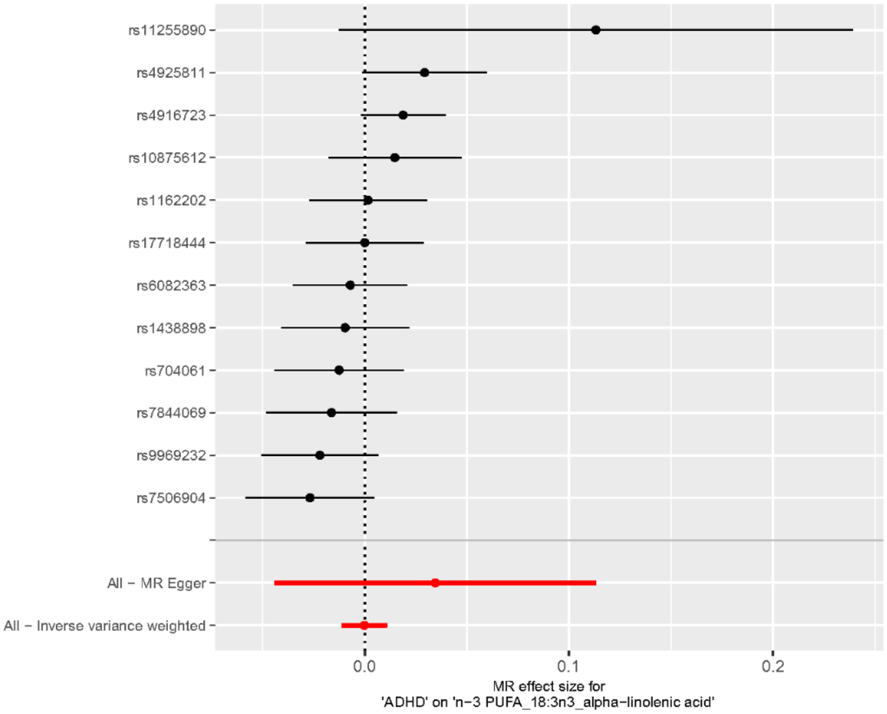

Figure S14a.

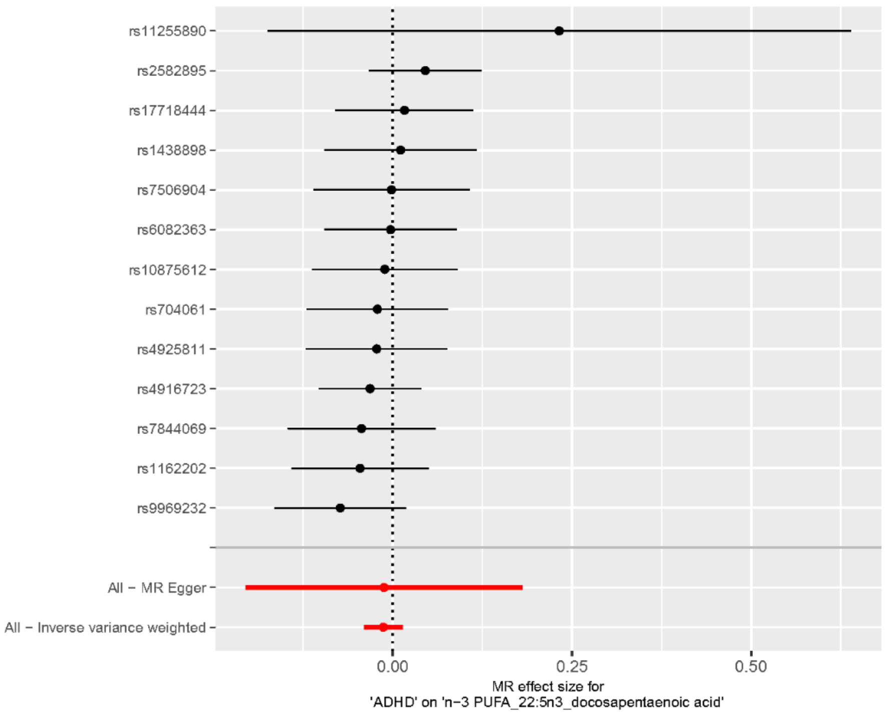

Figure S14b.

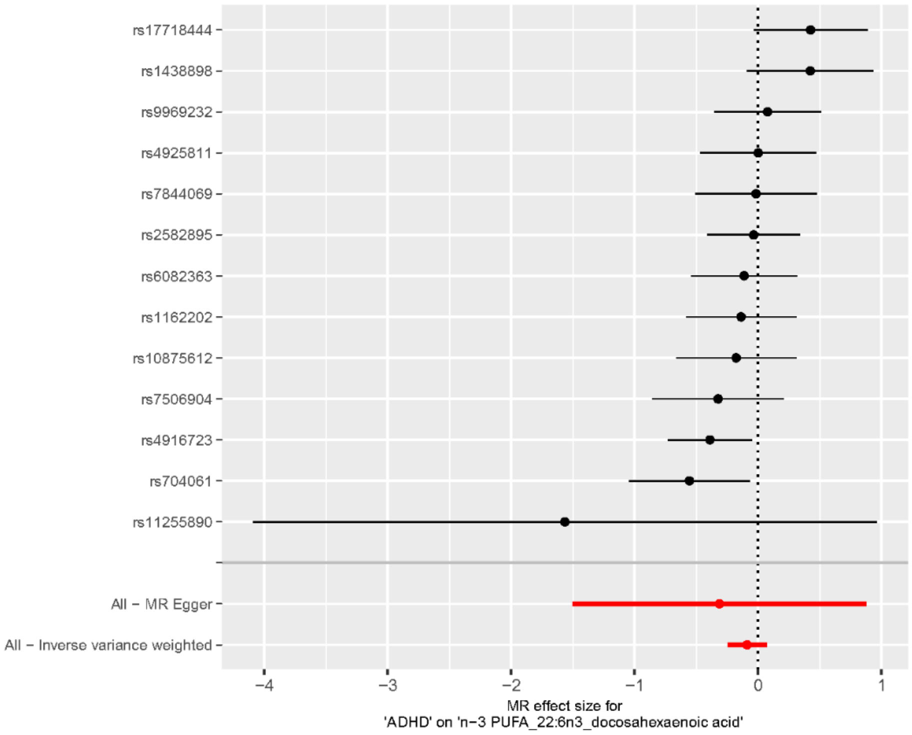

Figure S14c.

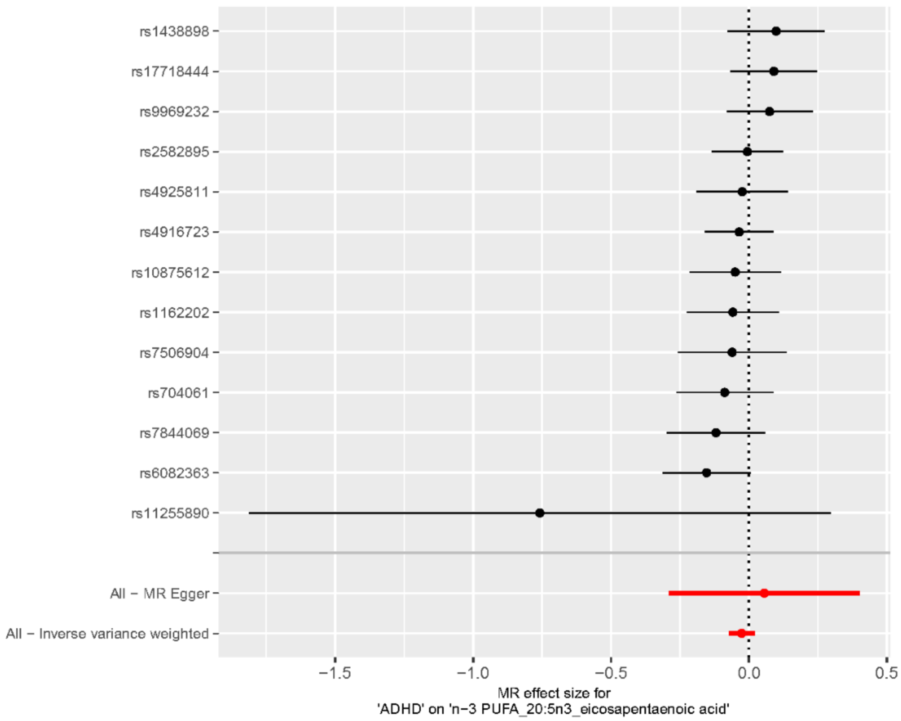

Figure S14d.

Figure S14. Forest plot for ADHD on n-3 PUFAs.

(A) Leave-one-out graph for n-3 ALA on ADHD. (B) Leave-one-out graph for n-3 DPA on ADHD. (C) Leave-one-out graph for n-3 DHA on ADHD. (D) Leave-one-out graph for n-3 EPA on ADHD.

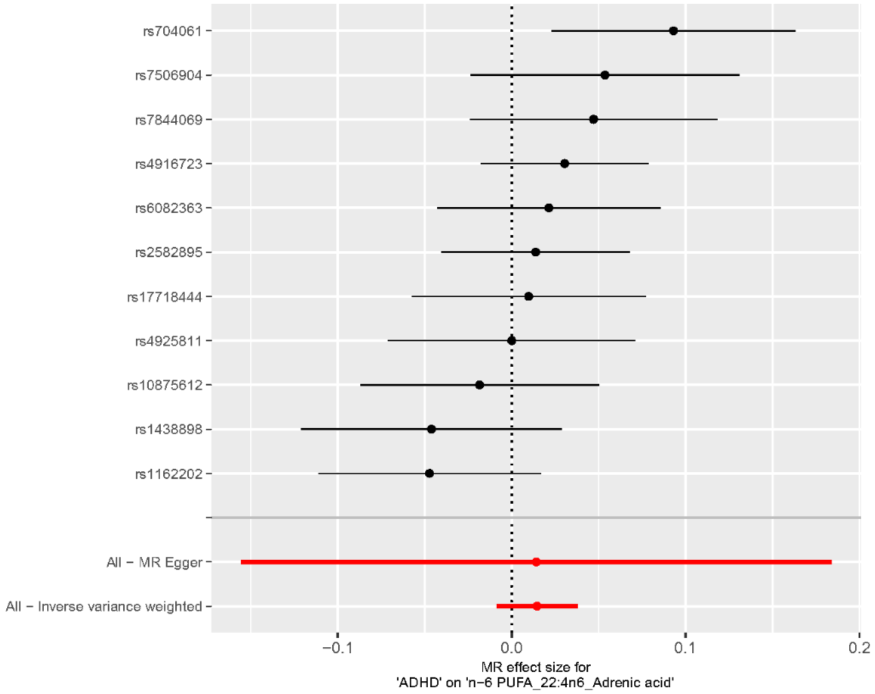

Figure S15a.

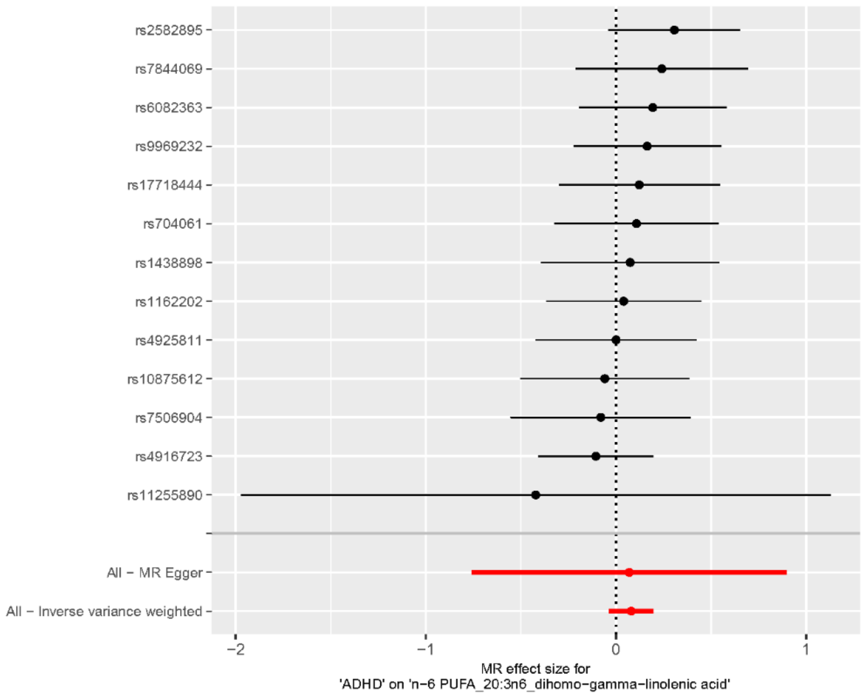

Figure S15b.

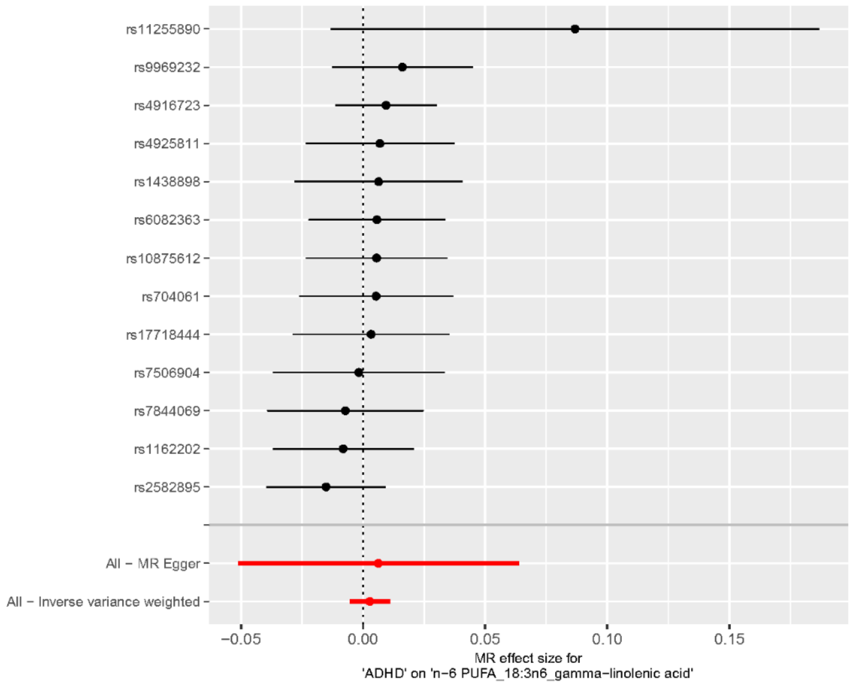

Figure S15c.

Figure S15. Forest plot for ADHD on n-6 PUFAs.

(B) Forest plot for n-6 Adrenic A on ADHD. (B) Forest plot for n-6 DGLA on ADHD. (C) Forest plot for n-6 GLA on ADHD.

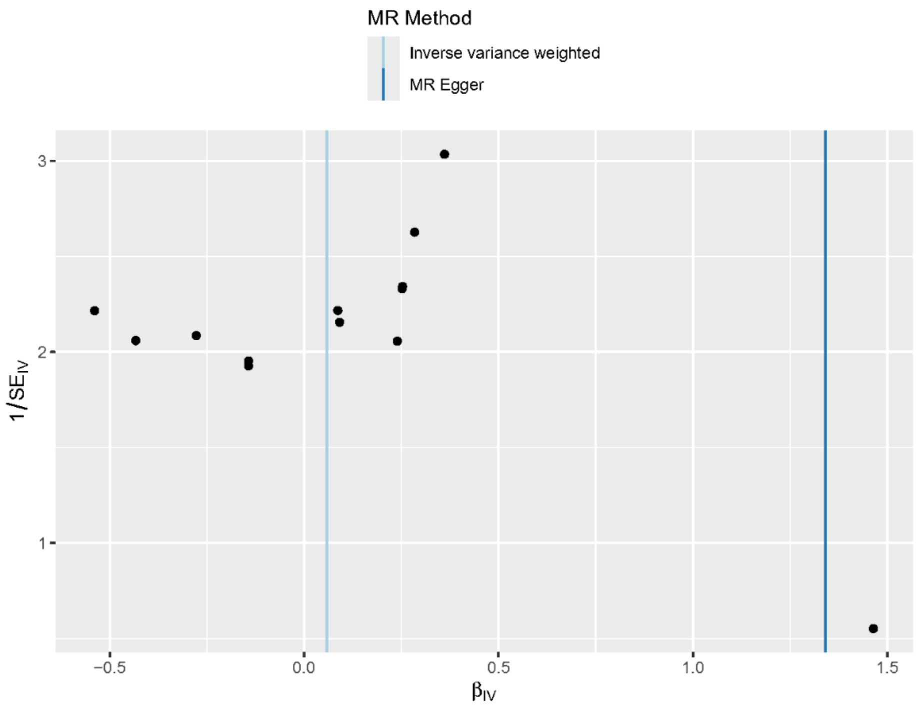

Figure S16a.

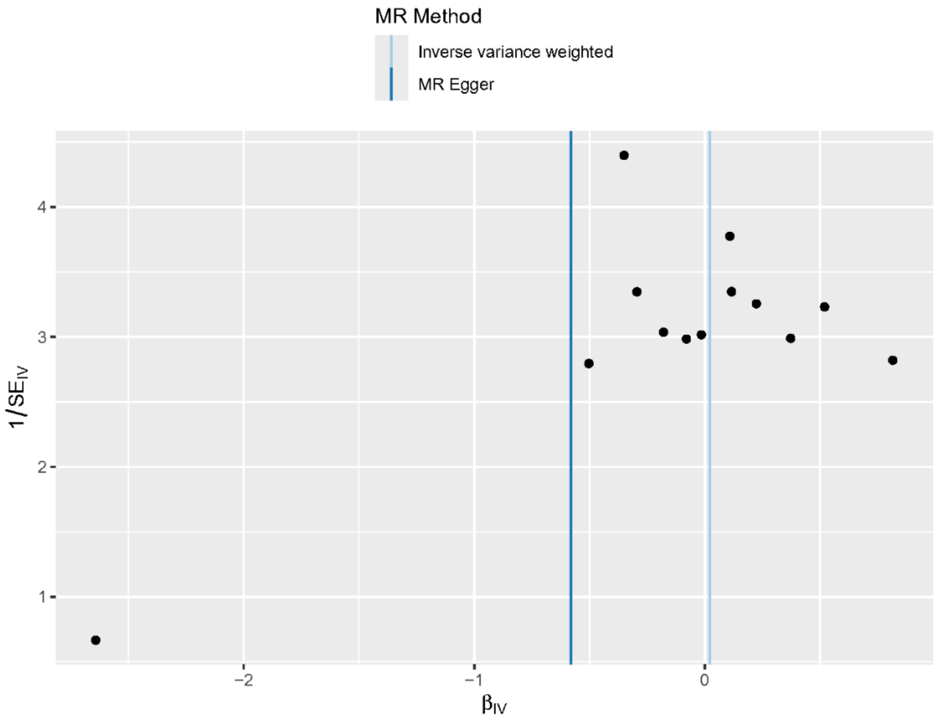

Figure S16b.

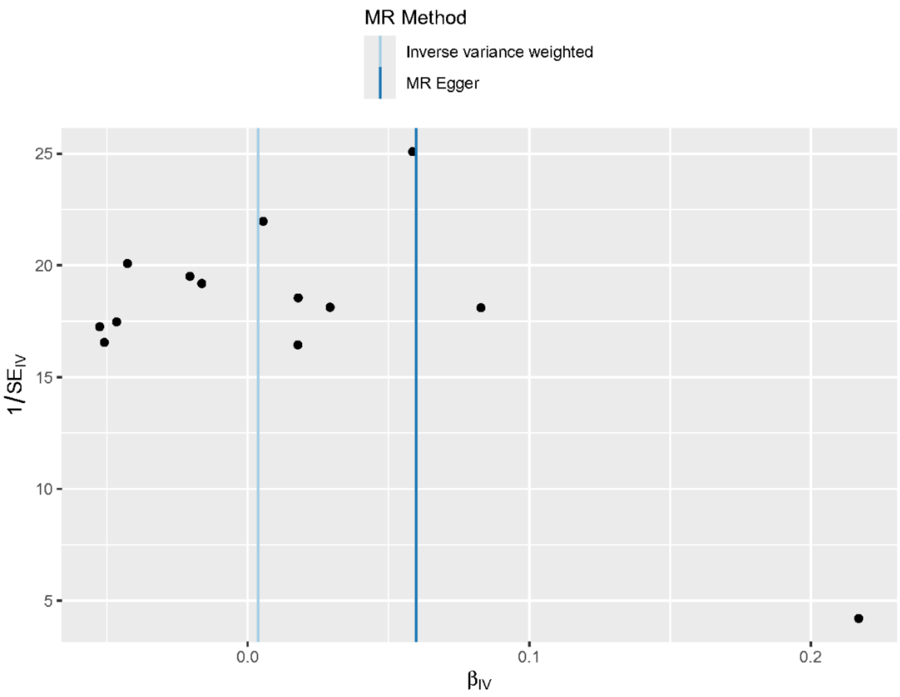

Figure S16c.

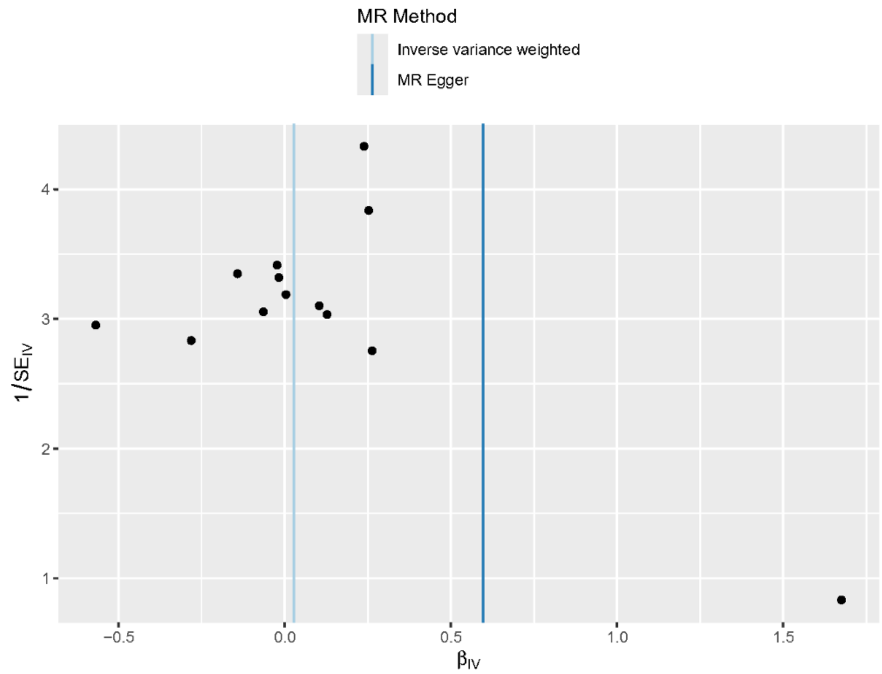

Figure S16d.

Figure S16. Funnel plot for ADHD on SFAs and MUFAs.

(A) Funnel plot for SFA 16:0 on ADHD. (B) Funnel plot for SFA 16:1n7 on ADHD. (C) Funnel plot for MUFA 18:0 on ADHD (D) Funnel plot for MUFA 18:1n9 on ADHD.

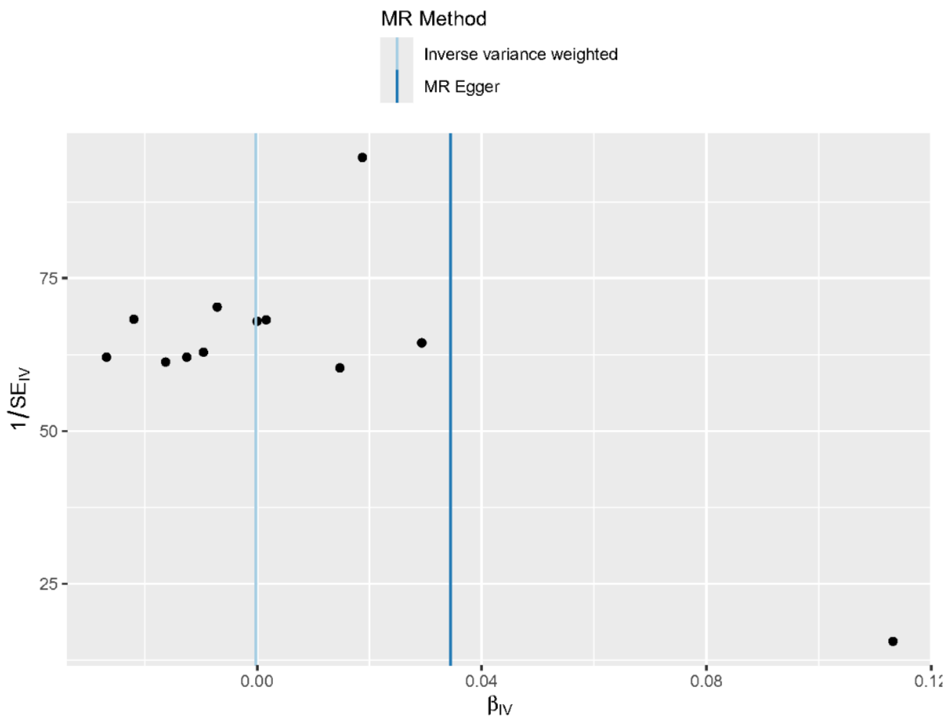

Figure S17a.

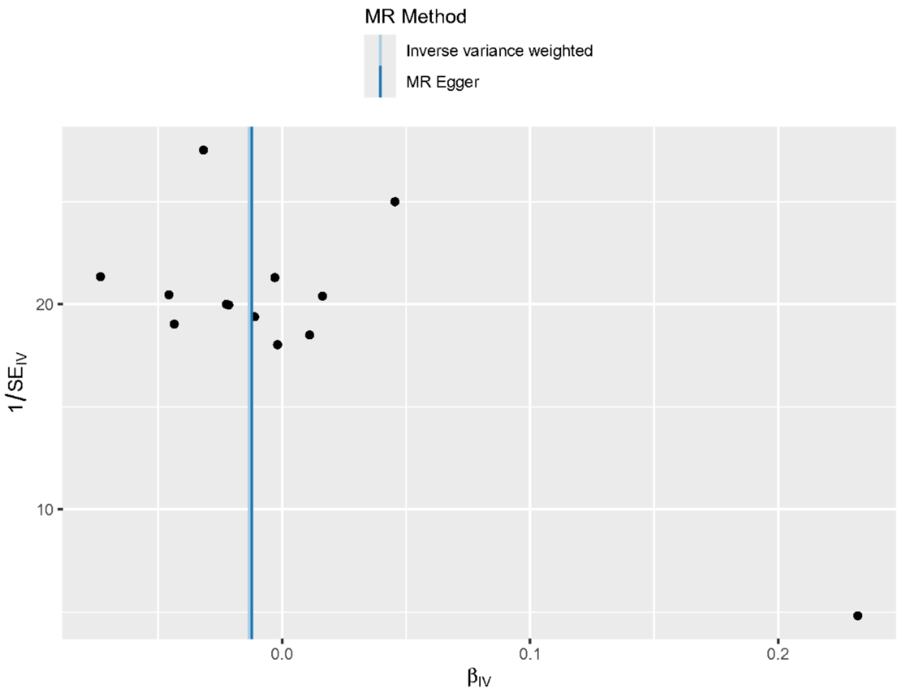

Figure S17b.

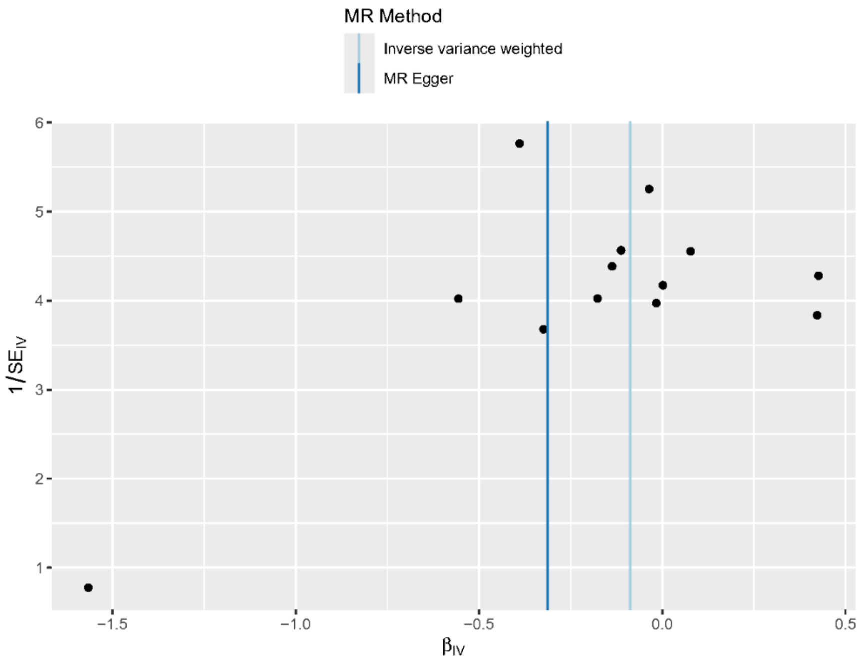

Figure S17c.

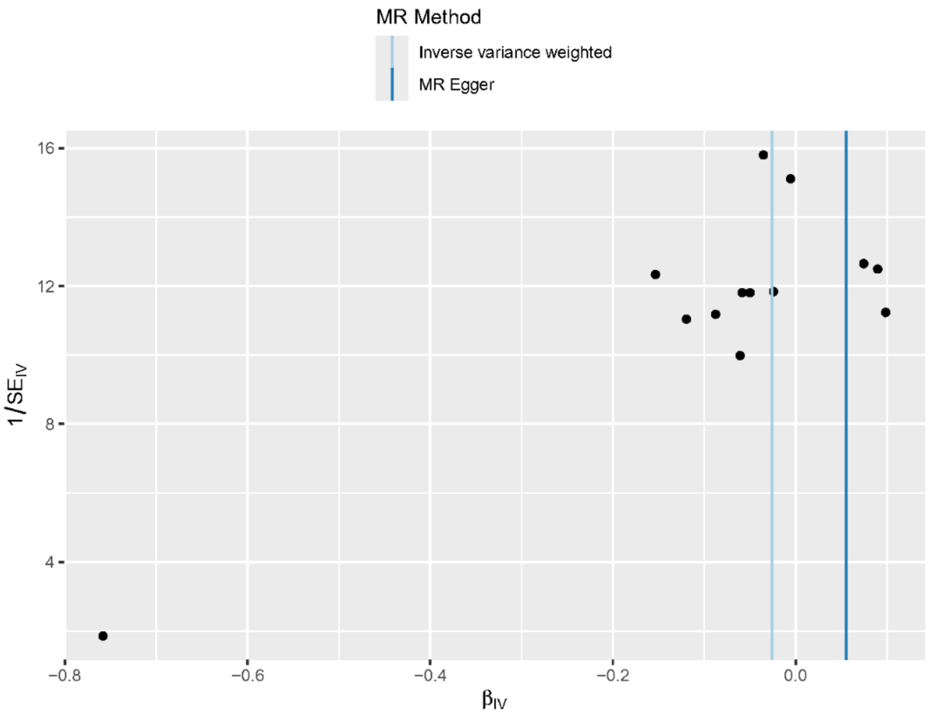

Figure S17d.

Figure S17. Funnel plot for ADHD on n-3 PUFAs.

(A) Leave-one-out graph for n-3 ALA on ADHD. (B) Leave-one-out graph for n-3 DPA on ADHD. (C) Leave-one-out graph for n-3 DHA on ADHD. (D) Leave-one-out graph for n-3 EPA on ADHD.

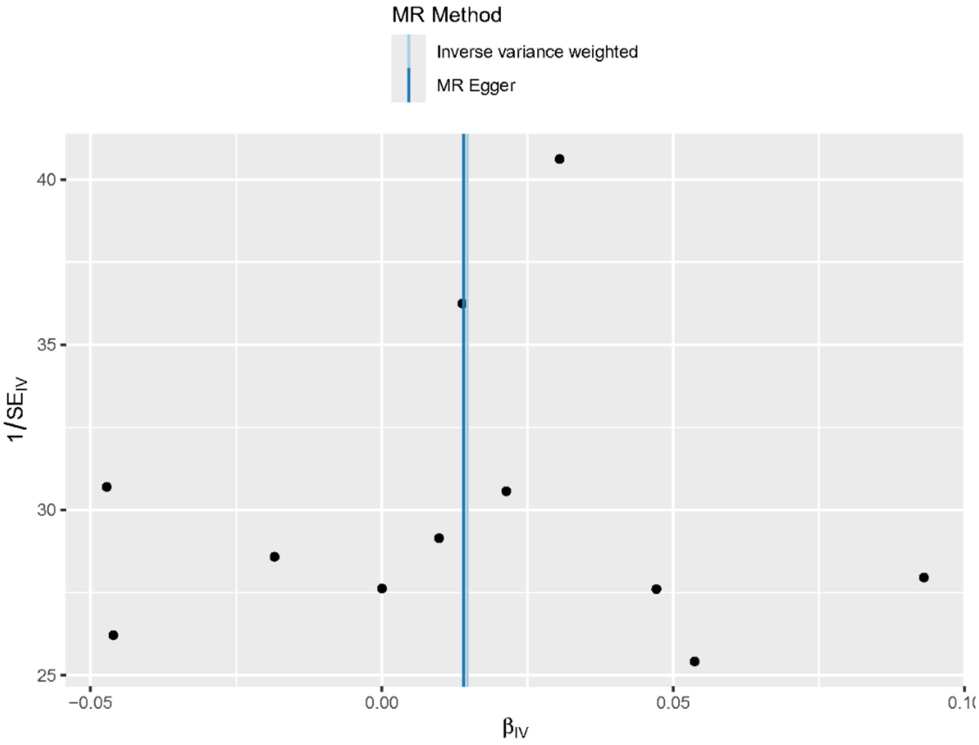

Figure S18a.

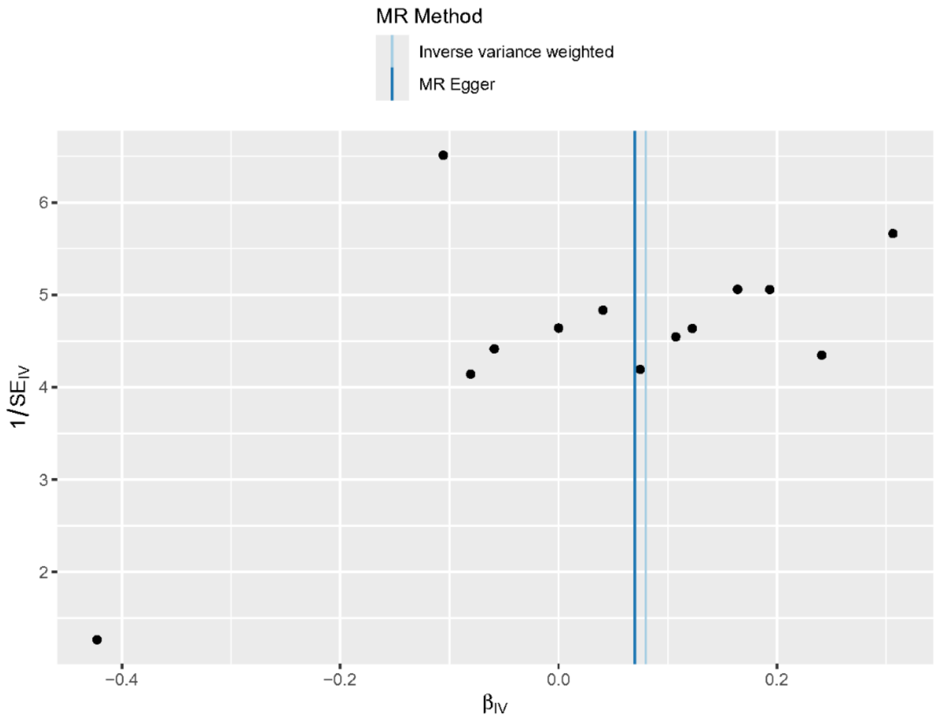

Figure S18b.

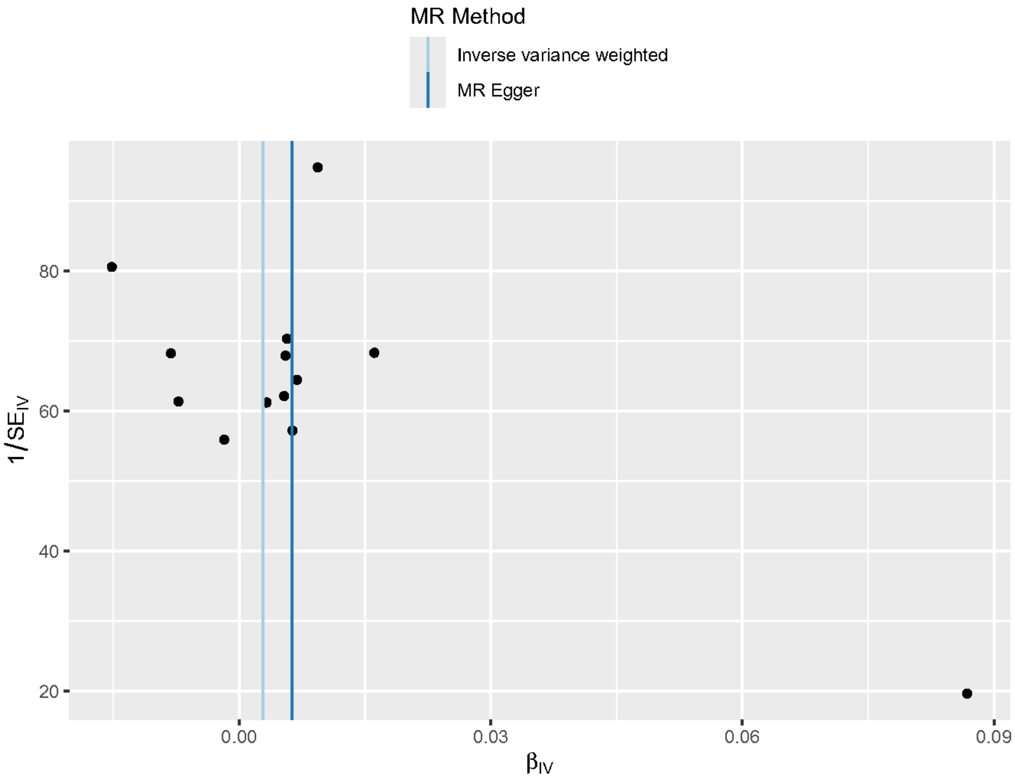

Figure S18c.

Figure S18. Funnel plot for ADHD on n-6 PUFAs.

(A) Funnel plot for n-6 Adrenic A on ADHD. (B) Funnel plot for n-6 DGLA on ADHD. (C) Funnel plot for n-6 GLA on ADHD.

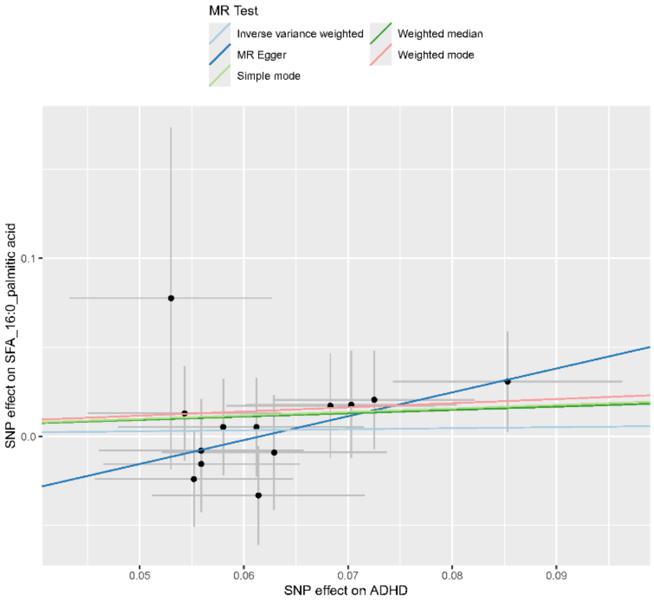

Figure S19a.

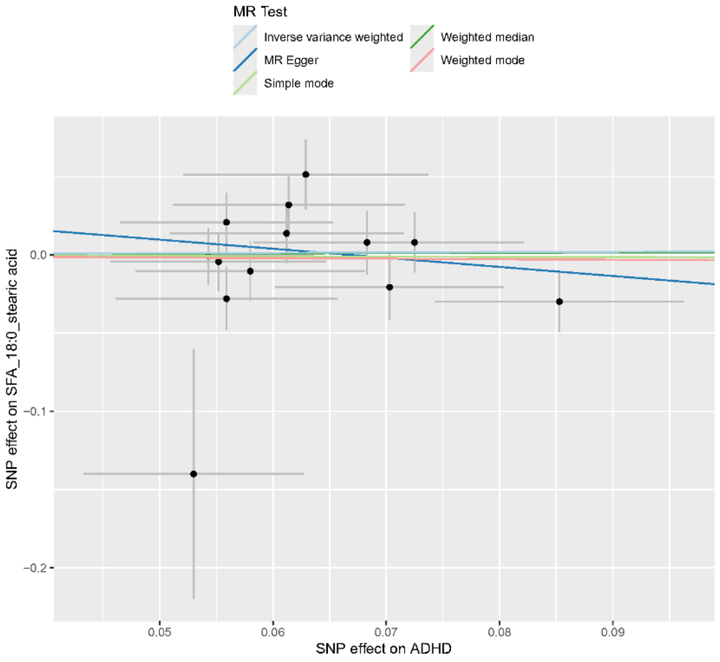

Figure S19b.

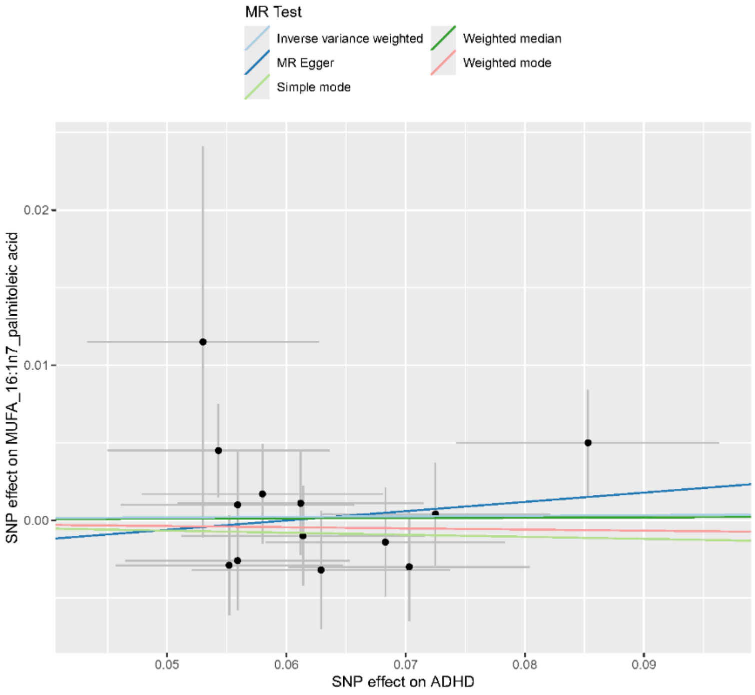

Figure S19c.

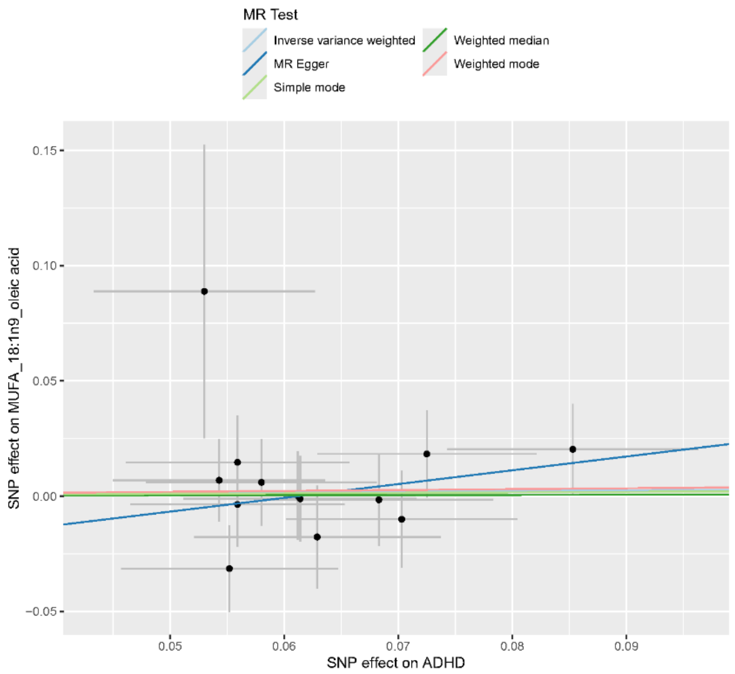

Figure S19d.

Figure S19. Scatter plots for ADHD on SFA and MUFA.

(A) Scatter plots for ADHD on16:0. (B) Scatter plots for ADHD on18:0. (C) Scatter plots for ADHD on 16:1n7. (D) Scatter plots for ADHD on 18:1n9.

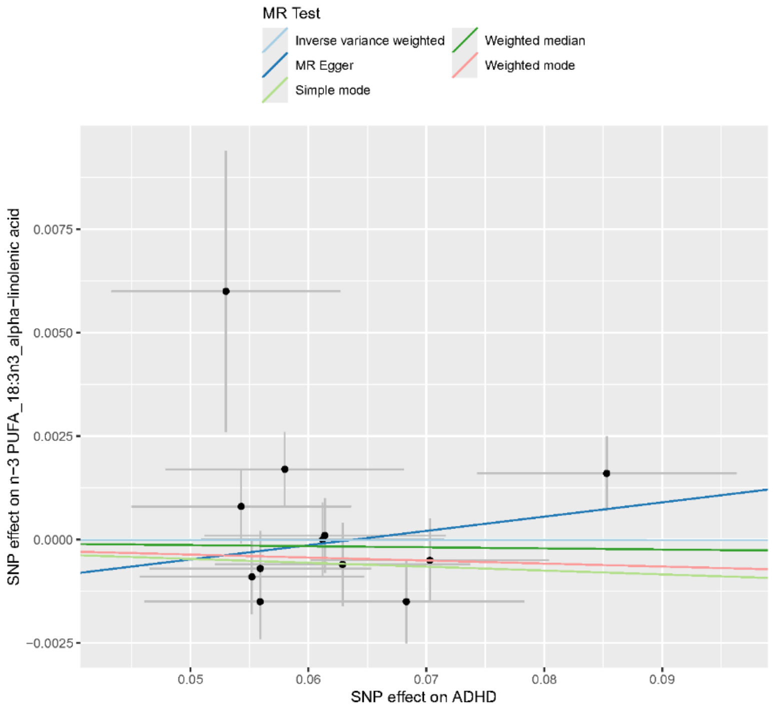

Figure S20a.

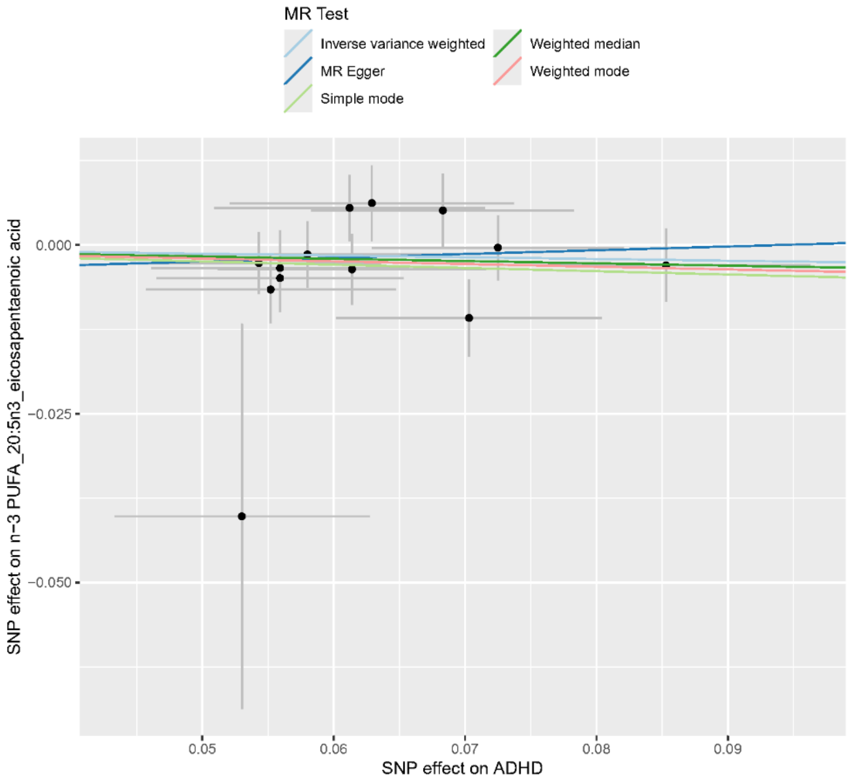

Figure S20b.

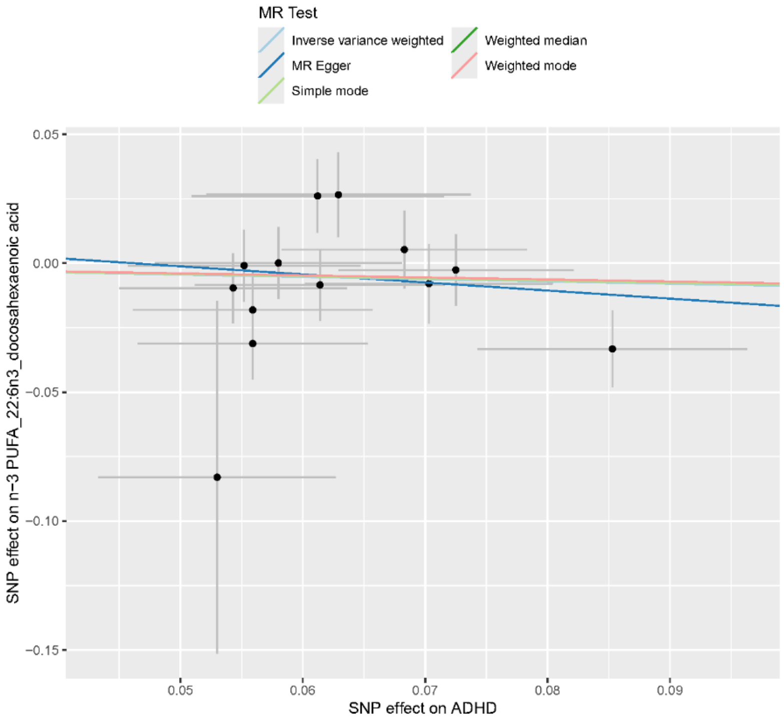

Figure S20c.

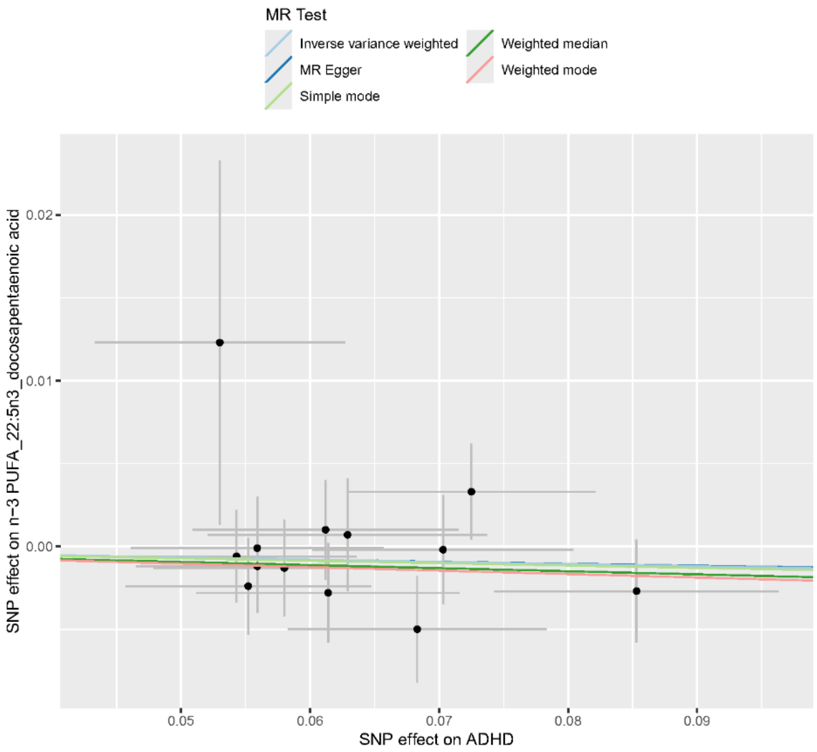

Figure S20d.

Figure S20. Scatter plots for ADHD on n-3PUFA.

(A) Scatter plots for ADHD on ALA. (B) Scatter plots for ADHD on EPA. (C) Scatter plots for ADHD on DHA. (D) Scatter plots for ADHD on DPA.

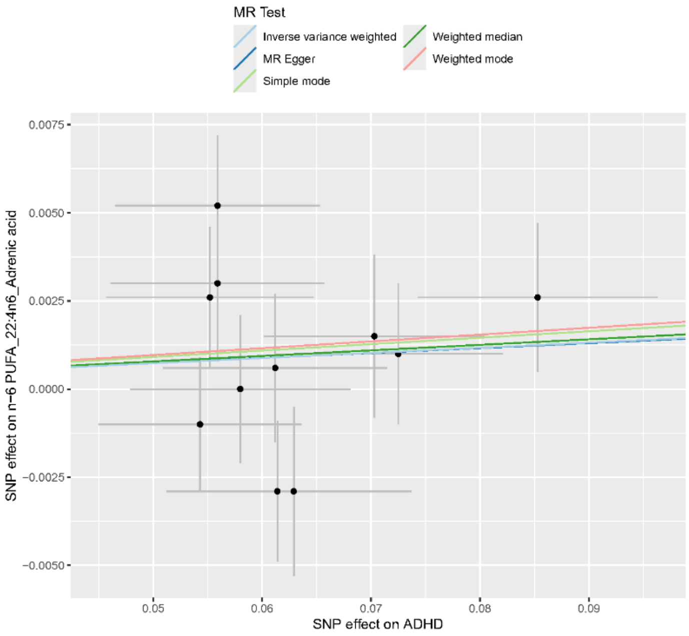

Figure S21a.

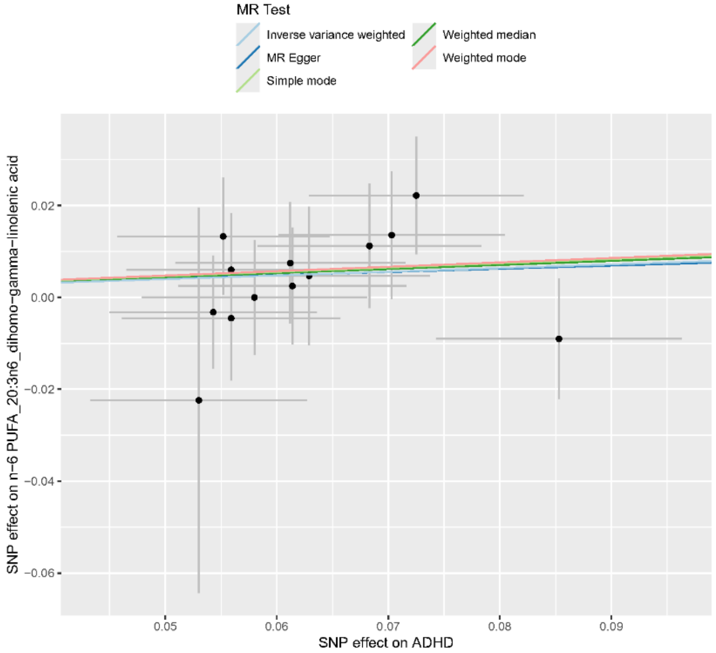

Figure S21b.

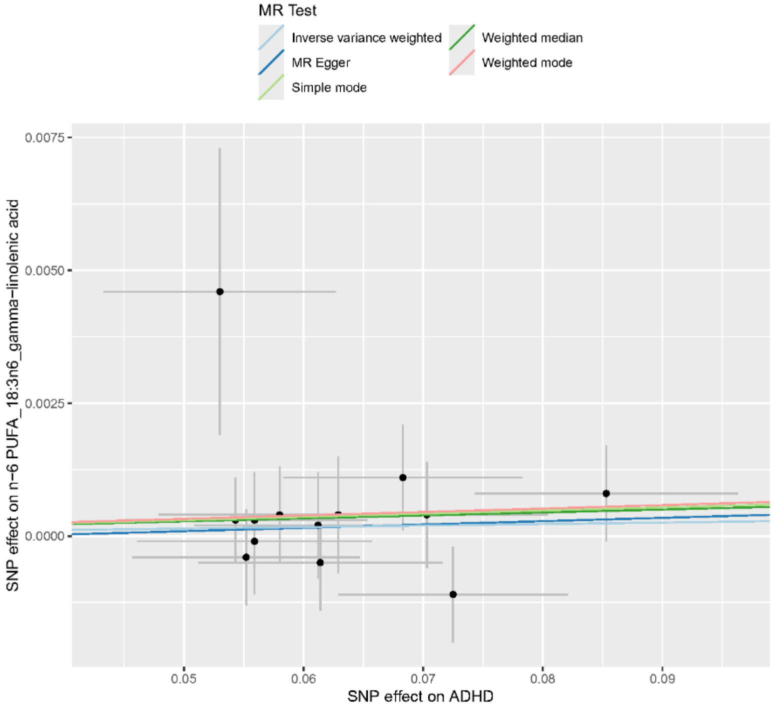

Figure S21c.

Figure S21. Scatter plots for ADHD on n-6PUFA.

(A) Scatter plots for ADHD on AdrA. (B) Scatter plots for ADHD on DGLA. (C) Scatter plots for ADHD on GLA.

1.2 Supplementary Tables

Table S1. 4 valid IVs used for MR analysis of SFA 16:0 on ADHD

SNP	Effect allele	Other allele	Effect allele frequency	Beta	SE	*P* value	R2	F
rs10809457	T	G	0.4077	-0.1394	0.0284	9.30E-07	0.0013	11.4638
rs2391388	A	C	0.5494	-0.1775	0.0266	2.72E-11	0.0025	22.2326
rs780093	T	C	0.4145	0.1261	0.0269	2.65E-06	0.0012	10.6346
rs994988	T	C	0.5333	-0.1395	0.0269	2.10E-07	0.0015	13.3523

Table S2. 4 valid IVs used for MR analysis of SFA 18:0 on ADHD

SNP	Effect allele	Other allele	Effect allele frequency	Beta	SE	*P* value	R2	F
rs102275	T	C	0.3636	0.1798	0.0193	1.33E-20	0.0045	40.3387
rs742614	A	G	0.4646	-0.095	0.0186	3.37E-07	0.0014	12.994
rs6675668	T	G	0.4899	-0.1651	0.0189	2.16E-18	0.0042	38.2937
rs4555772	A	G	0.4545	0.0888	0.0192	3.93E-06	0.0011	10.617

Table S3. 5 valid IVs used for MR analysis of MUFA 16:1n7 on ADHD

SNP	Effect allele	Other allele	Effect allele frequency	Beta	SE	*P* value	R2	F
rs102275	T	C	0.3636	-0.0238	0.0033	6.60E-13	0.0027	24.13164
rs7597155	A	G	0.4697	0.0149	0.0032	4.58E-06	0.001211	10.8112
rs780093	T	C	0.4091	0.0201	0.0033	9.80E-10	0.002012	17.96862
rs603424	A	G	0.202	-0.0326	0.0042	5.69E-15	0.002178	19.46124
rs3750720	T	C	0.202	0.0224	0.004	1.58E-08	0.001134	10.11942

Table S4. 3 valid IVs used for MR analysis of MUFA 18:1n9 on ADHD

SNP	Effect allele	Other allele	Effect allele frequency	Beta	SE	*P* value	R2	F
rs102275	T	C	0.6716	-0.23	0.0194	2.19E-32	0.007296	65.51171
rs3134950	A	C	0.6272	-0.0945	0.0194	1.14E-06	0.001296	11.56655
rs4731889	A	G	0.4515	-0.0856	0.0187	4.59E-06	0.001161	10.35708

Table S5. 2 valid IVs used for MR analysis of n-3 ALA on ADHD

SNP	Effect allele	Other allele	Effect allele frequency	Beta	SE	*P* value	R2	F
rs174547	T	C	0.3586	-0.0159	9.00E-04	3.47E-64	0.016194	145.9053
rs367543	T	C	0.4848	0.0052	0.0011	1.29E-06	0.001259	11.17478

Table S6. 1 valid IVs used for MR analysis of n-3 EPA on ADHD

SNP	Effect allele	Other allele	Effect allele frequency	Beta	SE	*P* value	R2	F
rs174538	A	G	0.3384	-0.0834	0.0052	5.37E-58	0.012991	116.6708

Table S7. 5 valid IVs used for MR analysis of n-3 DPA on ADHD

SNP	Effect allele	Other allele	Effect allele frequency	Beta	SE	*P* value	R2	F
rs780094	T	C	0.4091	0.0167	0.0029	9.04E-09	0.001808	16.05826
rs3734398	T	C	0.4394	-0.0404	0.0029	9.61E-44	0.010784	96.63196
rs12587311	T	C	0.5	-0.0142	0.0028	5.31E-07	0.00145	12.87547
rs7435	A	G	0.3232	-0.0173	0.0033	2.40E-07	0.001356	12.03698
rs174547	T	C	0.3586	0.0746	0.0028	3.79E-154	0.03683	338.9456

Table S8. 5 valid IVs used for MR analysis of n-6 Adrenic A on ADHD

SNP	Effect allele	Other allele	Effect allele frequency	Beta	SE	*P* value	R2	F
rs174550	T	C	0.3586	0.0484	0.0019	3.98E-140	0.034585	309.1279
rs3134950	A	C	0.492	-0.0097	0.0021	5.72E-06	0.001204	10.39933
rs721653	T	C	0.4495	0.009	0.002	4.74E-06	0.001161	10.03104
rs1962772	T	C	0.3485	0.01	0.002	8.96E-07	0.001315	11.3647
rs10761785	T	G	0.4899	0.0107	0.0021	6.51E-07	0.001503	12.99195

Table S9. 12 valid IVs used for MR analysis of n-6 DGLA on ADHD

SNP	Effect allele	Other allele	Effect allele frequency	Beta	SE	*P* value	R2	F
rs10805631	A	G	0.0012	1.1081	0.1305	2.04E-17	0.001215	10.5009
rs174555	T	C	0.7101	-0.3891	0.0141	4.57E-168	0.001621	14.00676
rs2493965	T	C	6.00E-04	0.7588	0.1152	4.54E-11	0.002222	19.21514
rs2906312	A	C	0.0131	1.1924	0.1801	3.57E-11	0.001443	12.4714
rs4761413	A	G	0.9995	-1.4325	0.1137	2.04E-36	0.00117	10.11137
rs4985155	A	G	0.6477	0.222	0.0128	1.56E-67	0.015606	136.7956
rs540613	T	C	0.1782	-0.1222	0.0208	4.35E-09	0.037267	334.0283
rs6083308	T	G	0.0175	1.0582	0.0639	1.12E-61	0.002717	23.51171
rs6085153	T	G	0.9864	-1.0157	0.1802	1.74E-08	0.003149	27.25925
rs771009	A	G	0.0197	1.0569	0.1425	1.20E-13	0.001184	10.22602
rs825338	A	G	8.00E-04	-0.7058	0.0636	1.33E-28	0.00334	28.91556
rs9514206	T	G	0.0109	1.2127	0.195	4.99E-10	0.011835	103.3512

Table S10. 48 valid IVs used for MR analysis of n-6 GLA on ADHD

SNP	Effect allele	Other allele	Effect allele frequency	Beta	SE	*P* value	R2	F
rs13210378	A	C	0.0017	-0.0647	0.0086	7.11E-14	0.001686	14.57264
rs11610576	T	C	0.0068	0.1413	0.0132	1.45E-26	0.001273	11.00032
rs11248254	A	G	0.0066	0.1211	0.0142	1.85E-17	0.001181	10.19974
rs7726400	T	C	0.006	0.1549	0.0138	2.12E-29	0.006488	56.34644
rs2669956	A	G	0.025	0.1746	0.0273	1.66E-10	0.001499	12.95764
rs259624	A	G	0.0236	0.0445	0.0057	7.85E-15	0.003114	26.9588
rs6589953	A	G	0.0136	0.1347	0.0101	1.86E-40	0.00253	21.88637
rs4497385	T	G	0.9881	-0.1345	0.0116	4.05E-31	0.003318	28.72725
rs12904618	A	G	0.0138	0.134	0.0105	3.05E-37	0.008117	70.61164
rs2121853	T	C	0.0138	0.1159	0.0107	4.16E-27	0.006796	59.04527
rs2377006	A	G	0.0228	0.1229	0.0081	1.34E-52	0.001822	15.74728
rs7546429	A	G	0.9824	0.0786	0.0044	4.77E-72	0.002173	18.78857
rs17441050	A	G	0.0261	0.1218	0.0071	1.59E-65	0.002004	17.3244
rs17159989	A	G	0.9872	-0.1174	0.0087	8.70E-42	0.00124	10.71139
rs4985155	A	G	0.6469	0.0062	9.00E-04	1.97E-12	0.002462	21.29728
rs4891073	T	C	0.9949	-0.161	0.0152	4.02E-26	0.003522	30.50134
rs11542415	T	C	0.0206	0.1234	0.0086	3.28E-47	0.001629	14.08061
rs10127697	A	G	0.9947	-0.1683	0.0153	4.60E-28	0.002879	24.9188

rs4760416	A	C	0.0087	0.0967	0.0136	1.11E-12	0.001958	16.92651
rs16843534	A	G	0.024	0.0453	0.003	2.68E-50	0.001303	11.25747
rs661706	A	G	0.0036	0.1651	0.0148	5.97E-29	0.00659	57.24395
rs7441263	A	G	0.0057	0.1547	0.0127	3.67E-34	0.002984	25.82498
rs8085143	T	C	0.987	-0.1417	0.0099	2.42E-46	0.003526	30.53191
rs12635046	T	C	0.9937	-0.1461	0.0146	1.94E-23	0.004075	35.31083
rs10800248	A	G	0.9854	-0.112	0.0104	4.19E-27	0.004491	38.92829
rs6704119	T	G	0.0143	0.1051	0.011	1.76E-21	0.00528	45.80143
rs6954039	A	G	0.0145	0.1353	0.0096	4.75E-45	0.009336	81.31592
rs6763739	A	G	0.0073	0.0828	0.0146	1.45E-08	0.001754	15.15961
rs8018207	T	C	0.0074	0.1491	0.0136	4.21E-28	0.00171	14.77752
rs4639338	T	C	0.006	0.1547	0.0136	8.68E-30	0.001162	10.04174
rs1506573	A	C	0.0179	0.0398	0.0061	7.21E-11	0.00153	13.22028
rs4560444	A	C	0.0097	0.0812	0.0083	8.02E-23	0.004186	36.27267
rs3936022	T	C	0.0201	0.1207	0.0084	3.18E-47	0.010104	88.07877
rs12196577	T	C	0.0045	0.1702	0.0156	7.51E-28	0.002279	19.7136
rs9264921	T	C	0.0048	0.1544	0.0128	1.90E-33	0.004795	41.57665
rs174547	T	C	0.6744	0.0156	9.00E-04	2.29E-72	0.016013	140.4247
rs5995311	A	G	0.9618	-0.0669	0.0059	9.37E-30	0.001294	11.17962
rs13126378	A	C	0.0189	0.055	0.0085	1.28E-10	0.001443	12.47089

rs12783205	T	C	0.9793	-0.1074	0.0081	7.65E-40	0.001769	15.29411
rs11849227	A	C	0.0056	0.1467	0.0148	3.05E-23	0.002512	21.73323
rs6083308	T	G	0.017	0.0308	0.0047	3.64E-11	0.001853	16.02221
rs4236845	T	G	0.0049	0.1564	0.0152	1.11E-24	0.006118	53.11379
rs6062730	T	C	0.9937	-0.1135	0.0154	1.76E-13	0.002437	21.08324
rs12334645	T	C	0.9995	-0.1837	0.0126	8.13E-48	0.001215	10.49363
rs1148061	T	C	0.0059	0.1521	0.014	1.97E-27	0.004952	42.9403
rs9347288	T	G	0.9925	-0.0943	0.0162	5.62E-09	0.001218	10.51942
rs10441549	A	G	0.0097	0.1122	0.0141	1.41E-15	0.001886	16.30652
rs4924269	A	G	0.997	-0.1595	0.0138	4.63E-31	0.0048	41.62195

Table S11. 13 valid IVs used for MR analysis of ADHD on SFA, MUFA, n-3 DPA, n-3 EPA, n-6DGLA, n-6GLA

SNP	Effect allele	Other allele	Effect allele frequency	Beta	SE	*P* value	R2	F
rs4916723	A	C	0.4747	0.011	9.48E-15	-0.0853	0.000133	29.99076
rs2582895	C	A	0.4091	0.0096	4.09E-14	0.072497	0.000122	27.5756
rs9969232	G	A	0.3485	0.01	9.98E-12	-0.0683	9.39E-05	21.18502
rs6082363	T	C	0.2828	0.0101	4.38E-12	0.0703	8.71E-05	19.65404
rs704061	T	C	0.4091	0.0094	2.30E-09	-0.0559	7.58E-05	17.10138
rs10875612	C	T	0.4798	0.0093	5.62E-09	-0.0543	7.54E-05	17.0172
rs1162202	C	T	0.3586	0.0102	1.92E-09	0.061396	7.39E-05	16.66781
rs4925811	T	G	0.4343	0.0101	8.30E-09	-0.058	7.18E-05	16.20488
rs17718444	C	T	0.3333	0.0103	2.87E-09	0.061199	6.96E-05	15.69027
rs7844069	T	G	0.3535	0.0095	6.74E-09	0.055198	6.84E-05	15.43177
rs7506904	G	A	0.3687	0.0098	1.24E-08	-0.0559	6.72E-05	15.14943
rs11255890	C	A	0.298	0.0097	4.14E-08	0.053	5.54E-05	12.49158
rs1438898	A	C	0.2374	0.0108	4.88E-09	0.0629	5.45E-05	12.28221

Table S12. 12 valid IVs used for MR analysis of ADHD on n-3ALA

SNP	Effect allele	Other allele	Effect allele frequency	Beta	SE	*P* value	R2	F
rs4916723	A	C	0.4747	0.011	9.48E-15	-0.0853	0.000133	29.99076
rs9969232	G	A	0.3485	0.01	9.98E-12	-0.0683	9.39E-05	21.18502
rs6082363	T	C	0.2828	0.0101	4.38E-12	0.0703	8.71E-05	19.65404
rs704061	T	C	0.4091	0.0094	2.30E-09	-0.0559	7.58E-05	17.10138
rs10875612	C	T	0.4798	0.0093	5.62E-09	-0.0543	7.54E-05	17.0172
rs1162202	C	T	0.3586	0.0102	1.92E-09	0.061396	7.39E-05	16.66781
rs4925811	T	G	0.4343	0.0101	8.30E-09	-0.058	7.18E-05	16.20488
rs17718444	C	T	0.3333	0.0103	2.87E-09	0.061199	6.96E-05	15.69027
rs7844069	T	G	0.3535	0.0095	6.74E-09	0.055198	6.84E-05	15.43177
rs7506904	G	A	0.3687	0.0098	1.24E-08	-0.0559	6.72E-05	15.14943
rs11255890	C	A	0.298	0.0097	4.14E-08	0.053	5.54E-05	12.49158
rs1438898	A	C	0.2374	0.0108	4.88E-09	0.0629	5.45E-05	12.28221

Table S13. 11 valid IVs used for MR analysis of ADHD on n-6 Adrenic acid

SNP	Effect allele	Other allele	Effect allele frequency	Beta	SE	*P* value	R2	F
rs4916723	A	C	0.4747	0.011	9.48E-15	-0.0853	0.000133	29.99076
rs6082363	T	C	0.2828	0.0101	4.38E-12	0.0703	8.71E-05	19.65404
rs704061	T	C	0.4091	0.0094	2.30E-09	-0.0559	7.58E-05	17.10138
rs10875612	C	T	0.4798	0.0093	5.62E-09	-0.0543	7.54E-05	17.0172
rs1162202	C	T	0.3586	0.0102	1.92E-09	0.061396	7.39E-05	16.66781
rs4925811	T	G	0.4343	0.0101	8.30E-09	-0.058	7.18E-05	16.20488
rs17718444	C	T	0.3333	0.0103	2.87E-09	0.061199	6.96E-05	15.69027
rs7844069	T	G	0.3535	0.0095	6.74E-09	0.055198	6.84E-05	15.43177
rs7506904	G	A	0.3687	0.0098	1.24E-08	-0.0559	6.72E-05	15.14943
rs1438898	A	C	0.2374	0.0108	4.88E-09	0.0629	5.45E-05	12.28221
rs2582895	C	A	0.4091	0.0096	4.09E-14	0.072497	0.000122	27.5756
